# Anti-Inflammatory and Anticancer Effects of Microalgal Carotenoids

**DOI:** 10.3390/md19100531

**Published:** 2021-09-23

**Authors:** Javier Ávila-Román, Sara García-Gil, Azahara Rodríguez-Luna, Virginia Motilva, Elena Talero

**Affiliations:** 1 Department of Biochemistry and Biotechnology, Universitat Rovira i Virgili, 43007 Tarragona, Spain; 2 Department of Pharmacology, Universidad de Sevilla, 41012 Seville, Spain; sargargil@alum.us.es (S.G.-G.); arodriguez53@us.es (A.R.-L.); motilva@us.es (V.M.)

**Keywords:** microalgae, carotenoids, inflammation, cancer, oxidative stress

## Abstract

Acute inflammation is a key component of the immune system’s response to pathogens, toxic agents, or tissue injury, involving the stimulation of defense mechanisms aimed to removing pathogenic factors and restoring tissue homeostasis. However, uncontrolled acute inflammatory response may lead to chronic inflammation, which is involved in the development of many diseases, including cancer. Nowadays, the need to find new potential therapeutic compounds has raised the worldwide scientific interest to study the marine environment. Specifically, microalgae are considered rich sources of bioactive molecules, such as carotenoids, which are natural isoprenoid pigments with important beneficial effects for health due to their biological activities. Carotenoids are essential nutrients for mammals, but they are unable to synthesize them; instead, a dietary intake of these compounds is required. Carotenoids are classified as carotenes (hydrocarbon carotenoids), such as α- and β-carotene, and xanthophylls (oxygenate derivatives) including zeaxanthin, astaxanthin, fucoxanthin, lutein, α- and β-cryptoxanthin, and canthaxanthin. This review summarizes the present up-to-date knowledge of the anti-inflammatory and anticancer activities of microalgal carotenoids both in vitro and in vivo, as well as the latest status of human studies for their potential use in prevention and treatment of inflammatory diseases and cancer.

## 1. Introduction

Microalgae are a vast group of prokaryotic and eukaryotic, mainly photoautotrophic, microorganisms that can be found individually or forming colonies. Moreover, these photosynthetic microorganisms make up the major group of living organisms in terms of species diversity on Earth, having colonized every type of ecological niche in both marine and terrestrial waters [1]. Currently, 50,000 species of microalgae have been described, but the number of new species is increasing yearly, being estimated up to 800,000. In this regard, although only a few of these aquatic microorganisms are able to grow in large-scale settings, microalgae have become an economically promising feedstock for bulk chemicals [2,3]. Moreover, the emergence of biotechnology in the 1960s led to the development of new laboratory and industrial methodologies to grow different species of microalgae. Since then, the worldwide research trends in the microalgal field have increased. In the last 20 years, a multitude of scientific publications have emerged around these aquatic microorganisms since they are a tremendously important source of bioactive molecules, being more diverse than those found in the terrestrial environment.

Firstly, microalga studies showed their potential to be considered by the biodiesel/bioethanol industry due to their high lipid content [4,5], besides promoting an ecological and socio-economic impact [6,7,8]. Although microalgae have been less studied than macroalgae, their advantages are associated with simple requirements, rapid generation times, and a higher capacity to modulate their metabolism in response to changing environmental conditions. Currently, microalgae remain attractive for the biodiesel industry but also for other sectors such as food, pharmaceuticals, or cosmetics. In this regard, many important drugs have traditionally been provided by terrestrial plants, fungi, and bacteria, but microalgae have become a sustainable resource for these biocomponents. Indeed, there is a current need to find new potential chemical structures for therapeutic use. Additionally, microalgae have raised the worldwide scientific interest since their capacity to synthetize new molecular structures according to seawater composition is widely known [9].

Many studies support microalgae as excellent sources of metabolites such as lipids, carbohydrates, proteins, phenolic compounds, vitamins, and carotenoids, which play physiological roles for themselves and their environment, with real applications in pharmaceutical and nutraceutical industries [10,11,12]. In this regard, only a few of these compounds, such as n-3 polyunsaturated fatty acids (PUFAs), phycobilins (phycoerythrin and phycocyanin), and carotenoids, including β-carotene, astaxanthin (ATX), zeaxanthin (ZX), and lutein (LUT), have been produced at an industrial scale. However, their low production yield in native microalgae and the difficulty in isolating by economically feasible means may be considered a production problem [2]. Nowadays, biotechnology considers microalgae as producers for a wide range of novel high-value products that have good market opportunities. However, the main challenges to obtain potential microalgal components are the high cost of operation, infrastructure and maintenance, selection of strains, dewatering, and commercial-scale harvesting. The manufacture and commercialization of microalgal products depend on market and financial affairs, among others. Furthermore, the study of their actual potential is limited by the lack of reliable statistical data of the microalga market. For this reason, the current scientific efforts are focused on basic technologies controlling several abiotic conditions to produce microalgal biomass, including different production methods such as open water, greenhouse ponds, and closed photobioreactors. Additionally, chemicals or certain culture conditions such as ultrasonic use by sonication [13,14,15] and genomic technologies [16] are currently being used in microalga cultivation to obtain high-value-added products [17]. These conditions are aimed in many cases at the food sector as nutritional supplements for vegetarian type diets but also as nutraceuticals. Hence, long-term research is needed to develop systems to create sustainable microalga-based products, since sustainability is a key concern, especially in today’s industrial environment. In this way, a multitude of recent international patent licenses [18] are focused on the optimization of microalga growth conditions as well as the system-level optimal yield to produce different bioproducts such as lipids for fuel, proteins for animal feed, or recombinant proteins for purposes of basic research, as well as biotechnological or dermatological/cosmeceutical use [19,20,21,22,23].

Carotenoids, which are one of the most abundant components in microalgae, have shown significant therapeutic potential due to their biological activities. In this context, the advances in biotechnology of microalgae have led to development of methods to increase their production. For example, the outdoor cultivation of *Muriellopsis* sp. (Chlorophyta) has been developed in order to produce high LUT and low metal content, to provide a product with antioxidant properties that may be used for animal feed and human consumption as a dietetic ingredient [24]. More recently, a method was carried out to efficiently extract eicosapaentanoic acid (EPA) and fucoxanthin (FX) from the microalga *Phaeodactylum tricornutum* (Bacillariophyta) [25]. Furthermore, in the last few years, a multitude of studies have shown the industry and academic interest in the potential of carotenoids from microalgae in different industrial sectors. In this regard, a variety of patents and scientific publications in which microalgae, or part of them, are used as functional food or nutraceuticals providing therapeutical potential have been developed. Recently, a patent has been licensed for a microalga-derived carotenoid mixture, which contains diatoxanthin from the microalga Euglena (Euglenozoa) as the main component, besides ZX and alloxanthin. This diatoxanthin-rich product prevents diabetes by suppressing the increment in blood glucose through ingestion along with a high-glycemic index food [26]. In addition, *Chlorella sorokiniana* (Chlorophyta), a microalga rich in glutathione, α-tocopherol, and carotenoids, was reported to have beneficial effects in counteracting oxidative stress preserving mitochondrial liver function in an experimental model of hyperthyroidism in rats [27]. Additionally, anti-inflammatory, antioxidant, and anticancer properties of microalgal carotenoids have been widely demonstrated in different experimental models, but to date there are only a few studies in humans.

The present review summarizes the major findings on microalgal carotenoids with a potential role in inflammation, oxidative stress, and cancer since carotenoids are one of the most abundant compounds in microalgae and they can represent an important commercial outlet.

## 2. Microalgal Carotenoids

Carotenoids are tetraterpenes obtained from dimerization of geranylgeranyl pyrophosphate in photosynthetic organisms such as plants, including macro- and microalgae, bacteria, some fungi, or some invertebrates [28]. They make up the most abundant lipid-soluble pigments in nature, being responsible for the white, yellow, orange, or red range of colors. There are two types of carotenoids: carotenes, which are hydrocarbon carotenoids such as α- and β-carotene, and xanthophylls, which are oxygenate derivatives, including ZX, ATX, FX, LUT, α- and β-cryptoxanthin (BCX), and canthaxanthin (CX). Carotenoids are essential nutrients for mammals, since they are unable to synthesize them. For this reason, a dietary intake of these compounds is required. The major dietary sources of carotenoids are fruits and vegetables, legumes, cereals, egg yolk, and mammals’ milk, as well as micro- and macroalgae [29]. 

Currently, lycopene, β-carotene, CX, ZX, ATX, and LUT are the main carotenoids produced on a large scale for food products, animal feeds, cosmetics, and pharmaceutical sectors. Their increasing commercial applications have led to a growing market demand of these bioactives. Thus, microalgae have emerged as a rich biosustainable source of carotenoids, with *Arthrospira* (formerly *Spirulina*) (Cyanobacteria), *Chlorella*, *Dunaliella*, and *Haematococcus* (Chlorophyta) being the most common producers of β-carotene, LUT, ATX, FX, ZX, and violaxanthin, among others [30].

### 2.1. β-Carotene

**Figure 1:**
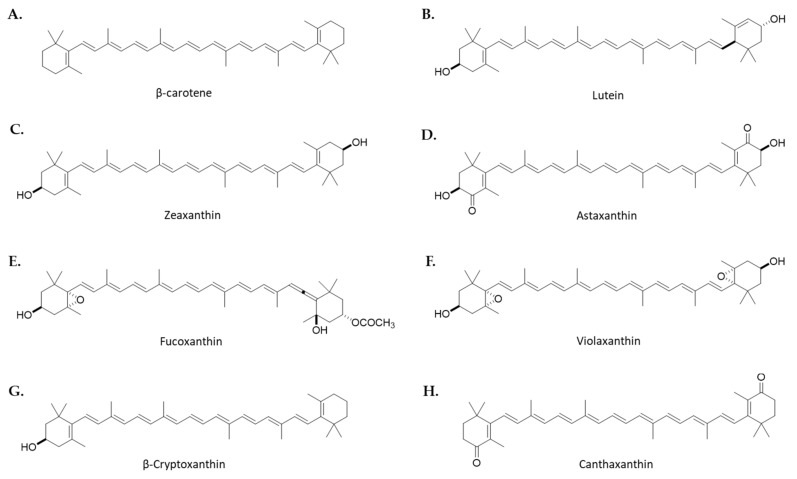
Chemical structures of the main functional carotenoids found in microalgae. Carotenes: β-Carotene (**A**) and xanthophylls: Lutein (**B**), Zeaxanthin (**C**), Astaxanthin (**D**), Fucoxanthin (**E**), Violaxanthin (**F**), β-Cryptoxanthin (**G**) and Canthaxanthin (**H**).

β,β-carotene, or more commonly named β-carotene (Figure 1A), is the most well-known carotenoid found in many fruits and vegetables [29]. This tetraterpenoid is a vitamin A precursor when consumed and digested. Currently, β-carotene is used as a natural colorant and antioxidant in the food industry [31]. The main microalgal source of β-carotene for the market is *Dunaliella salina* (Chlorophyta), which is able to accumulate up to 8% of dry weight [32]. In addition, the microalgae *Arthrospira platensis* (formerly *Spirulina platensis*) (Cyanobacteria) [33], *Chlamydomonas reinhardtii* (Chlorophyta) [34], *Isochrysis galbana* (Haptophyta), *Phaeodactylum tricornutum* (Bacillariophyta), and *Tetraselmis suecica* (Chlorophyta) have also shown high levels of this carotenoid in large-scale systems [35].

### 2.2. Lutein 

3*R*,3′*R*,6′*R*-βε-carotene-3,3′-diol or LUT (Figure 1B) is a natural carotenoid synthetized in plants as well as algae. It is an orange-yellow xanthophyll widely used as a feed additive and a food coloration agent in industry [36]. Despite being present in a multitude of vegetables and fruits, its low content has led to the search for new sources of this carotenoid such as in microalgae [37]. In this regard, LUT is accumulated on a large scale in several species of *Chlorella* such as *C. sorokiniana*, *Chromochloris* *zoofingiensis* (formerly *Chlorella zoofingiensis*), and *Auxenochlorella* *prothecoides* (formerly *Chlorella protothecoides*) [38], as well as in *Dunaliella salina* [39], the strain *Chlamydomonas* sp. JSC4 [40], and *Tetraselmis suecica* [41].

### 2.3. Zeaxanthin

β,β-carotene-3,3′-diol or ZX (Figure 1C) is a yellow-orange xanthophyll found mainly in dark green leafy vegetables and egg yolks. It has been reported that, like LUT, ZX is accumulated in the central retina and has photoprotective effects against damage by intense light. Regarding microalgae, this xanthophyll has been obtained from the cyanobacteria *Synechocystis* sp. and *Microcystis aeruginosa* [42], as well as the microalgae *Nannochloropsis oculata* (Ochrophyta, Eustigmatophyceae) [43], *Chloroidium saccharophilum* (formerly *Chlorella saccharophila*) [44], and *Dunaliella* sp. [45], and red algae such as *Porphyridium purpureum* (formerly *Porphyridium cruentum*) (Rhodophyta) [35], *Phaeodactylum tricornutum* (Bacillariophyta) [46], or *Heterosigma akashiwo* (Ochrophyta, Raphidophyceae) [47].

### 2.4. Astaxanthin

3,3′-dihydroxy-β,β′-carotene-4,4′-dione or ATX (Figure 1D) is a xantophyll mainly found in microalgae, marine invertebrates, some fishes like salmon and trout, and even in the feathers of some birds, contributing to their red-orange pigmentation [48]. However, the main source of AXT is *Haematococcus lacustris* (formerly *Haematococcus pluvialis*) (Chlorophyta)*,* whose content may represent up to 3% of dry weight [49], but this xantophyll can also be found in other microalgae such a *Chromochloris zofingiensis* [50], *Chlorococcum* sp. [51], *Dunaliella salina*, *Tetraselmis suecica* [41], *Scenedesmus quadricauda* PUMCC 4.1.40. (Chlorophyta) [52], and *Asterarcys quadricellulare* PUMCC 5.1.1 (Chlorophyta) [53].

### 2.5. Fucoxanthin

(3S,3′S,5R,5′R,6S,6′R,8′R)-3,5′-dihydroxy-8-oxo-6′,7′-didehydro-5,5′,6,6′,7,8-hexahydro-5,6-epoxy-β,β-caroten-3′-yl acetate, also named FX (Figure 1E), is an orange-colored xanthophyll mainly found in marine environments. This carotenoid is present in a variety of macroalgae, but also in a multitude of species of microalgae such as *Isochrysis* sp. (Haptophyta), *Odontella aurita* [54,55], *Nitzschia laevis* (formerly *Nitzschia amabilis*) [56], and *Chaetoceros neogracili* (formerly *Chaetoceros gracilis*) (Bacillariophyta), the coccolithophore *Pleurochrysis carterae* (Haptophyta, Coccolithophyceae) [57], *Phaeodactylum tricornutum* (Bacillariophyta) [46], and the microalga strain *Pavlova* sp. OPMS 30543 (Haprophyta) [58]. 

### 2.6. Violaxanthin 

5,6,5′,6′-diepoxy-5,6,5′,6′-tetrahydro-β,β-carotene-3,3′-diol, also called violaxanthin (Figure 1F), is a natural orange xanthophyll, which may enzymatically be transformed into ZX when the light energy absorbed by plants exceeds the photosynthesis capacity [59]. It is a pigment found in different plants as well as macro- and microalgae such as *Nannochloropsis oceanica* (Ochrophyta, Eustigmatophyceae) [60], *Jaagichlorella luteoviridis* (formerly *Chlorella luteoviridis*) [61], the strain *Tetraselmis striata* CTP4 (Chlorophyta) [62], and Eustigmatophyte strains such as *Chlorobotrys gloeothece*, *Chlorobotrys regularis*, and *Munda aquilonaris* (formerly *Characiopsis aquilonaris*) [63].

### 2.7. β-Cryptoxanthin

(1R)-3,5,5-trimethyl-4-[(1E,3E,5E,7E,9E,11E,13E,15E,17E)-3,7,12,16-tetramethyl-18-(2,6,6-trimethylcyclohexen-1-yl)octadeca-1,3,5,7,9,11,13,15,17-nonaenyl]cyclohex-3-en-1-ol, also called BCX (Figure 1G), is a natural orange xanthophyll mainly found in fruits of plants, including in orange rind, papaya, or apples, besides egg yolk and butter. This carotenoid is also found, but in a lower concentration than other carotenoids, in different species of microalgae such as *Phaeodactylum tricornutum* (Bacillariophyta) [64], *Auxenochlorella pyrenoidosa* (formerly *Chlorella pyrenoidosa*) (Chlorophyta) [65], and *Porphyridium purpureum* (Rhodophyta) [66,67].

### 2.8. Canthaxanthin

β,β-carotene-4,4’-dione or CX (Figure 1H) is a red-orange xanthophyl widely used as a cosmetic and food colorant as well as in poultry as a feed additive. This carotenoid was firstly isolated from the edible mushroom *Cantharellus cinnabarinus*. Moreover, this pigment is present in bacteria, algae, crustacea, some fungi, and various species of fish including carp and golden mullet [68]. Regarding microalgae, this xanthophyll has been found in *Haematococcus lacustris* [69], *Chromochloris zoofingiensis* [70], *Chlorococcum* sp. [51], *Dunaliella salina* [71], *Chlorella vulgaris* (Chlorophyta) [72], *Scenedesmus quadricauda* PUMCC 4.1.40. [52], *Asterarcys quadricellulare* PUMCC 5.1.1 [53], *Picochlorum* sp. SBL2. [73], and *Dactylococcus dissociatus* MT1 (Chlorophyta) [74].

## 3. Inflammation and Cancer

Acute inflammation is a key component of the response of the immune system to injury and infection that involves the stimulation of defense systems against foreign components and organisms, and the healing and/or repair of damaged tissue. This process is recognized by some cardinal signs, including heat, redness, pain, or swelling. It is characterized by the activation of immune cells, synthesis of proinflammatory mediators, is usually localized and self-limited, and normally returns to homeostasis [75]. Acute inflammation requires suppression of proinflammatory mediators and induction of anti-inflammatory/proresolution mediators as well as the disappearance of leukocytes from the damage area, and the restoration of tissue functionality [76]. However, if the acute inflammatory process is excessive and is not resolved, it may lead to tissue damage, resulting in chronic inflammation, and ultimately fibrosis, with loss of tissue functionality. Consequently, the failure of the resolution of inflammation is strongly associated with the development of many chronic disease states of complex evolution: arthritis, neurodegenerative diseases, metabolic syndrome and associated pathologies, allergy, and periodontal diseases, as well as tumoral processes, among others [77,78,79]. It has been reported that an adequate diet, a healthy lifestyle, or the establishment of certain preventive strategies, including drugs, nutraceuticals, or components of functional foods, may contribute to the control of inflammatory processes. This section summarizes the main mediators and cells involved in acute and chronic inflammatory responses, as well as describes the link between inflammation and cancer and the main molecular pathways implicated in these processes.

The defense systems of the body are mediated by sequential and coordinated responses called innate and adaptive immunity. The innate immune system is the first line of defense against microbes; it is mediated by cellular elements, including macrophages, neutrophils, dendritic cells, natural cytolytic lymphocytes, or mast cells, as well as by biochemical mechanisms involving agglutinins, the complement system, and many types of lectins, which circulate and provide rapid responses [80]. Macrophages play a pivotal role in all phases of inflammation: in the initiation, help to neutralize and remove pathogens and damaged cells through phagocytosis, and later lead to the termination of inflammation by tissue repair and remodeling responses [81]. Based on responses to different in vitro stimuli appears the macrophage polarization concept of M1/M2 differentiation [82]. M1 macrophages are induced by proinflammatory factors, such as lipopolysaccharide (LPS), cytokines through granulocyte–macrophage colony-stimulating factor, and tumor necrosis factor-α (TNF-α), among others. Later, interleukins (IL), such as IL-1β and IL-6, reactive oxygen species (ROS), and nitric oxide (NO) are released, acting as inducers of a polarized Th1 response. M2 macrophages present a characteristic phagocytic ability of scavenging molecules, as well as produce suppressive mediators, including mannose or galactose receptors and polyamines [83]; they are activated by exposure to Th2-related cytokines (IL-13, IL-4), or anti-inflammatory mediators, including IL-10 and transforming growth factor beta (TGF-β). Accumulating data indicate that M2 macrophages play an important role in microorganism clearance, tissue repair, and inflammation resolution. Nevertheless, some evidence has also shown that M2 macrophages may enhance tumor growth depending on the microenvironmental conditions of this cell population [84]. 

Adaptative immunity is a response that increases in magnitude and capabilities with each successive exposure to an antigenic stimulus; it is mediated by lymphocytes T and B (cellular immunity and humoral immunity, respectively) and their products. Several types of T cells are detected in the blood, at different stages: effector T lymphocytes can differentiate into T helper (Th) and cytotoxic effector lymphocytes (Tc), which act against cells infected by cytoplasmic intracellular pathogens. Th lymphocytes are differentiated into Th1, which are involved in the elimination of intracellular pathogens (viruses) or phagocytable extracellular organisms (bacteria and fungi), and into Th2, which characteristically act against helminths. In addition, Th cells can differentiate into Th17, T follicular helper cells (Tfh), and T regulatory (Treg) lymphocytes, which exert their activity against commensal bacteria. Regarding B lymphocytes, unmatured cells migrate from bone marrow to spleen and are transformed into B T1 and B T2 lymphocytes; B T2 could be transformed into follicular B cells depending on the signals received through their receptors. In any case, B lymphocytes are T cell-dependent antigen-presenting cells [85].

From a different point of view but complementary to the previous classification, the activation of immune cells regulates two basic effector systems aimed to eliminate potential offending agents: phagocytosis (cellular response) and cytotoxicity [86,87]. Phagocytosis is an effective mechanism of elimination of infectious agents. Although most immune cells are capable of phagocytosis, the most characteristic phagocytes are macrophages and polymorphonuclear neutrophils, which provide a powerful oxidative system and a wide variety of proteolytic enzymes to degrade the phagocytosed material. On the other hand, cytotoxicity is cell-mediated toxicity, and an alternative defense mechanism when phagocytosis cannot resolve the problem: tumor cells, response to viruses, infections by intracellular or large pathogens. These functions are performed by different cell types: (1) eosinophilic and basophilic polymorphonuclear cells that actively participate in the defense against helminths and protozoa by using a receptor for immunoglobulin IgE, which recognizes the pathogen. These cells produce substances with high cytotoxic activity (neurotoxin, cationic protein, or histamine) capable of blocking or killing microorganisms much larger than them. Mast cells also perform this function, as well as participate in the activation of the inflammatory reaction and in allergic processes [88]. (2) Natural cytotoxic cells (NK) that are especially active against tumor cells and cells infected by viruses. These cells are of lymphoid lineage, but do not possess a variable antigen receptor like lymphocytes. They recognize their targets through non-polymorphic receptors, or by using receptors for immunoglobulin–Fc fragment, a process known as antibody-dependent cell cytotoxicity. NK cells kill their targets by activating apoptosis programs [89]. (3) T-CD8+, or cytotoxic lymphocytes (T-CTL): when a T-CTL lymphocyte recognizes an antigen–major histocompatibility complex (Ag-MHC), it kills the cell that presents it in a similar way to the NK cell, secreting cytotoxic factors (perforins and granzymes), or interacting with membrane proteins of the target cell. Regarding CD8+ cells, they attack virus-infected cells, where they activate pathways of apoptosis (TNFR1 or Fas, among others). (4) T-CD4 + or Th lymphocytes: although their cytotoxic capacity is much lower than that of T-CTL, and their main function is the activation of other cell types of the immunity response, Th lymphocytes can kill other cells by secreting granzymes or by expressing proapoptotic ligands, including Fas-L or TNF-related apoptosis-inducing ligand, which activate apoptosis programs. (5) Finally, the complement system, which is particularly capable of opsonizing particles to be removed by phagocytes but can also damage membranes and cause cell necrosis [86].

As mentioned above, after the active phase of inflammation, coordinated resolution responses are initiated to prevent chronic inflammation establishment and restore homeostasis [76]. During the initial phase of the acute inflammatory response, the well-known proinflammatory mediators comprising prostaglandins (PGs) and leukotrienes are synthesized from arachidonic acid (ARA) by cyclooxygenases (COXs) and lipoxygenases. Later, in the resolution phase of inflammation, another pathway involving ARA metabolization, via cytochrome P450, is initiated, leading to the production of epoxyeicosatrienoic acids (EETs). These partially oxidized lipidic compounds, oxylipins, may participate in the activation of anti-inflammatory processes and the clearance of cellular debris as well as inhibit numerous proinflammatory cytokines [90]. Additionally, EET, and other epoxy fatty acids, stimulate the production of specialized proresolving mediators (SPMs), such as lipoxins, by shifting ARA metabolism, to support inflammation resolution [91]. In addition to omega-6 arachidonic acid-derived lipoxins, n-3 PUFA-derived SPMs are synthesized from EPA and docosahexaenoic acid and encompass resolvins, protectins, and maresins [76,92]. These lipid autacoids are involved in down-regulation of proinflammatory cytokines/chemokines, inhibition of neutrophil infiltration, and induction of macrophage phagocytosis [93]. Dietary sources of PUFA include fish and algae, and more recently, microalgae [94].

It has been reported that chronic non-resolving inflammation increases the risk of developing cancer. Epidemiological data have evidenced that more than 20% of detected tumors have in their origin, or in their evolution, an important inflammatory component [95]. Inflammation-associated cancer is a long-term process that requires the transformation of normal cells to tumor cells through premalignant lesions. In the inflammation–cancer connection, extrinsic and intrinsic pathways are involved; the extrinsic pathway comprises microbial infections, such as *Helicobacter pylori* and its relationship with gastric cancer, tobacco and lung cancer development, or ultraviolet exposure and its association with skin tumors. Intrinsic factors include mutations in oncogenes and suppressor/repair genes and epigenetic defects, as well as modifications in the immune system [28,96].

Nevertheless, it has also been described that in a previously detected tumor, not linked to a previous inflammatory process, inflammation is present in the surrounding area of the tumor, promoting cancer progression to achieve the malignant phenotype, tissue remodeling, metastasis, and angiogenesis, or the suppression of immune response [97]. Regarding microenvironmental components, it has been reported that macrophages are the most abundant cells in tumor environments and their function in cancer is contradictory. In some types of cancer, these cells have a crucial role in cancer progression and evasion of immune response, which has been correlated to poor prognosis. However, in some gastrointestinal cancers, a large number of macrophages has been related to good prognosis [98]. These findings may be explained by the presence of different macrophage populations in tumor tissues and suggest that macrophage assessment could be used as an innovative prognostic marker.

Given the tumor-promoting effects of macrophages, the development of compounds to target these cells may be a promising strategy for cancer treatment. In this line, different approaches are being considered to inhibit their recruitment, such as inhibition of chemoattractants (C-C chemokine receptor type 2/CCL2 signaling) [99], reduction in macrophages number with bisphosphonates, and inhibition of differentiation and survival (colony stimulating factor 1 (CSF-1)/CSF-1R axis) [100]. However, these types of strategies that focus on general selection have shown limited clinical success [101]. Interestingly, new approaches are being directed to reprogramming macrophages towards an anticancer phenotype. In this line, it has been reported that CD40 agonist antibodies activate antitumor macrophages [102] and other antibodies inhibit the CD47 surface molecule in tumor cells, leading to macrophage-mediated tumor cell phagocytosis [103]. Ongoing studies will let to know the diversity of macrophages in cancer tissues and their clinical interest for cancer prognostic and treatment.

From a molecular and intracellular point of view, during the inflammatory process, a coordinated activation of several signaling pathways is triggered, including phosphatidylinositol 3-kinase/protein kinase B (PI3K/Akt), mitogen-activated protein kinase (MAPK), Janus kinase/signal transduction and activator of transcription (JAK/STAT), or the key transcriptional element nuclear factor-kappa B (NF-κB) that interacts with different nuclear or cytoplasmic elements, including PPAR-γ, which is capable of inhibiting NF-κB activation and the consequent production of numerous cytokines [104,105,106]. The activation through the innate immune system occurs by pattern recognition receptors (PRRs) and NOD-like receptors (NLRs). Some of these receptors are associated with a multiprotein complex, called the inflammasome, with NOD-LRR and pyrin domain-containing 3 (NLRP3) being the best characterized and involved in the activation of caspase-1 and proteolytic maturation of IL-1β and IL-18 [107]. It has been reported that ROS, produced primarily at the mitochondrial level, are involved in NLRP3 activation [108,109]. Furthermore, exposure to ROS can also activate nuclear factor erythroid 2-related factor 2 (Nrf2), which migrates into the nucleus and induces the expression of genes with antioxidant response element-like sequences in their promoter, such as heme oxygenase-1 (HO-1), peroxiredoxins, and glutamate-cysteine ligase [110,111]. Nrf2 protects normal cells against ROS-induced DNA damage as well as malignant cells against chemotherapy [112]. Nrf2 also stimulates several oncogenes unconnected to antioxidant activity, including matrix metalloproteinase-9 (MMP-9), TNF-α, and vascular endothelial growth factor A (VEGF-A) [113]. Additionally, the aryl hydrocarbon receptor (AHR) is a ubiquitously expressed ligand-activated transcription factor with remarkable physiological roles; it is a key component that can integrate infective or environmental signals into innate and adaptive responses. AHR activity seems to regulate barrier organs, such as the skin, lung, or gut. The liver is exposed to gut-derived alimentary or microbial AHR ligands and, additionally, generates AHR ligands, including metabolic enzymes, such as cytochrome P450, which produces toxic metabolites and increases ROS production [114]. In contrast, AHR ligands from intestinal microbiota are involved in the maintenance of epithelial integrity as well as the generation of the anti-inflammatory IL-22 [115].

On the other hand, necroptosis has been described as programmed necrotic cell death induced by cytokines, Toll-like receptors (TLR), or ROS. After a necroptotic stimulus, the receptor-interacting protein kinase 1 (RIP1)/RIP3 complex phosphorylates and activates the mixed lineage kinase domain-like protein (MLKL), which oligomerizes and translocates to the plasma membrane, forming pores and leading to cell lysis [116]. Additionally, it is interesting to highlight the sirtuin (SIRT) family in the inflammation context. Many of them are histone deacetylases involved in cellular pathways related to the structure and function of tissues, and with capacity to control processes, including inflammation or cancer. Between them, the SIRT1 isoform has a special role in ROS-induced cell death, and SIRT6 has an interesting function in cancer and autophagy. Moreover, SIRT3 shows a potential therapeutic role in different pathologies, including cardiovascular diseases, where a SIRT3 deficiency has been associated with necroptosis, and NLRP3 activation in a diabetic cardiomyopathy [117].

Regarding the role of ROS in the inflammatory response, it has been reported that minimal ROS concentrations may be essential in many intracellular signal processes connected with cell proliferation, apoptosis, or defense against microorganisms. However, high doses or inadequate removal of ROS generate oxidative stress, which cause macromolecular damage and metabolic dysfunctions [118]. Lipid peroxidation is a serious consequence of oxidative stress since the derived products, epoxides, can interact with nucleophilic structures of the cell or with nucleic acids and cause structural damage and mutations. Consequently, an adequate equilibrium between antioxidants and oxidants to maintain cellular homeostasis is necessary [119]. In aerobic organisms, there are a variety of antioxidant, enzymatic, and non-enzymatic systems with protective properties; enzymes include glutathione peroxidase, superoxide dismutase (SOD), and catalase, which are present in various cell sites, such as the cytosol, endoplasmic reticulum, peroxisomes, and mitochondria. This latter organelle is able to generate almost 90% of ROS, mainly through coenzyme Q [120]. In addition, there are substances capable of neutralizing ROS, such as alpha-tocopherol (vitamin E), ascorbic acid (vitamin C), vitamin A, glutathione (GSH), flavonoids, phenolic acids, and carotenes.

**Figure 2:**
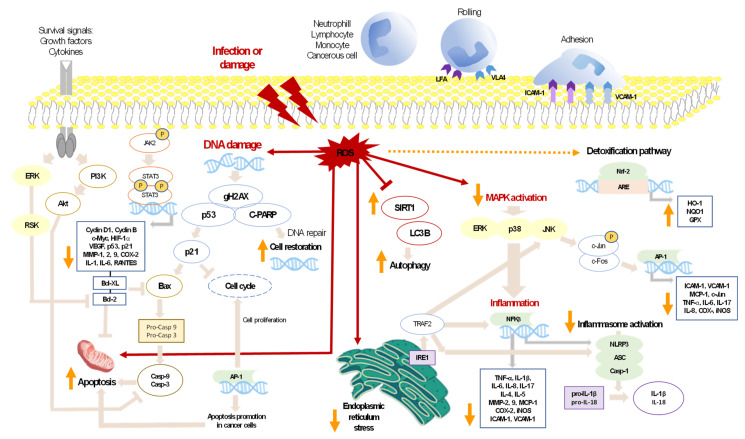
Carotenoids’ interaction on major signaling pathways implicated in inflammation or cancer. The figure shows the bioactivity of the carotenoids for different type of cells. Red arrows show the effect of the presence of ROS on several activities in the cell; dashed orange arrow refers to the detoxification pathway that is triggered when ROS are produced; pink arrows show the interconnections of different mediators; orange arrows refer to the bioactivities produced by the different microalgal carotenoids.

As regards cancer, it is known that malignant cells can maintain elevated intracellular ROS levels due to different causes, including mitochondrial damage, rapid metabolism, lipid peroxidation, or metal ion formation, such as copper and iron, as well as reduction in endogenous antioxidants [121]. In cancer cells, the role of ROS is controversial since they have been shown to have both pro- and antitumorigenic functions, depending on the concentrations. In this line, moderate ROS levels can induce cell survival, angiogenesis, and metastasis through activation of the MAPK pathway, which in turn stimulates NF-κB and the subsequent up-regulation of MMPs and VEGF [118]. Nevertheless, regarding its antitumorigenic role, high intracellular ROS levels can induce apoptosis of cancer cells by activation of the proapoptotic proteins Bax, p21, and p27, among others, and a decrease in the antiapoptotic Bcl-2 and Bcl-xL [121]. Therefore, these proapoptotic properties of ROS can serve as a crucial therapeutic strategy to destroy tumor cells. In this line, it is interesting to highlight the role of carotenoids in cancer since these compounds can serve as pro-oxidants in cancerous cells, leading to ROS-induced apoptosis. Furthermore, when they are administered with ROS-stimulating cytotoxic drugs, carotenoids can decrease the dangerous effects of these drugs on normal cells by their antioxidant properties, as well as increase cytotoxicity of drugs towards cancer cells by a pro-oxidant mechanism. Therefore, this synergistic effect of carotenoids with anticancer drugs may be an innovative strategy for cancer treatment [121,122]. Figure 2 shows a diagram of the main targets and signaling pathways in which microalgal carotenoids have shown a direct or indirect ability to modify different signaling pathways. 

## 4. Anti-Inflammatory Activity of Carotenoids 

Section 4 and Section 5 summarize the recent up-to-date studies (since 2010 up to June 2021) reporting the anti-inflammatory and anticancer activities of microalgal carotenoids both in vitro and in vivo, as well as the latest status of human studies for their potential use in the prevention and treatment of different inflammatory diseases and cancer. In addition, the molecular mechanisms underlying these effects are described. The most relevant anti-inflammatory and anticancer activities of carotenoids, as well as the main microalgal sources, are summarized in Table 1.

### 4.1. β-Carotene

#### 4.1.1. In Vitro Studies

Different preclinical in vitro studies have evidenced that β-carotene can prevent and reduce diabetes, which is a chronic low-grade inflammatory disease associated with common complications. In this respect, this compound was evaluated in human endothelial cells isolated from umbilical cord veins (HUVECs) of women suffering from gestational diabetes. The results evidenced that β-carotene prevented vascular inflammation and reduced the nitro-oxidative state induced by TNF-α in HUVECs. These effects were related to an attenuation of vascular cell adhesion molecule 1 and intercellular adhesion molecule 1 (ICAM-1) expression, reduction in NF-κB activation, and suppression of peroxynitrite levels. These findings suggest that a carotenoid-rich diet could play an important role in the prevention of cardiovascular complications of diabetes [123]. Similar findings were obtained in TNF-α-stimulated HUVECs of healthy women after treatment with β-carotene [124]. It has been reported that oxidative stress produced in adipose tissue results in dysregulated production of proinflammatory adipokines by adipocytes, which is related to the pathogenesis of diabetes and obesity. β-Carotene attenuated oxidative stress-induced inflammation via a decrease in the adipokines monocyte chemoattractant protein-1 (MCP-1) and RANTES and an increase in adiponectin in 3T3-L1 adipocytes. The mechanisms underlying these effects were linked to the inhibition of the activation of NF-κB, activator protein-1 (AP-1), and signal transducer and activator of transcription 3 (STAT3) transcription factors [125]. In the same line, the cardioprotective role of a low dose of β-carotene in the prevention of ROS-induced atherosclerosis has been reported in cardiomyoblasts through up-regulation of Nrf2, activation of autophagy, and inhibition of NF-κB and apoptosis [126]. 

In addition, it has been demonstrated that β-carotene suppressed NLRP3 inflammasome activation in mouse bone marrow macrophages [127] as well as inhibited JAK2/STAT3 and c-Jun N-terminal kinase (JNK)/p38 MAPK signaling pathways in LPS-stimulated macrophages [128]. Similarly, this compound suppressed the pseudorabies virus-induced inflammatory response, which mimics human herpes simplex virus inflammation, in RAW 264.7 macrophages, via reductions in NF-κB and MAPK activation [129].

#### 4.1.2. In Vivo Studies

A number of in vivo models have evidenced the anti-inflammatory effects of β-carotene. Regarding gastrointestinal disorders, oral treatment with this carotenoid at the doses of 5, 10, and 20 mg/kg for 28 days suppressed dextran sodium sulfate (DSS)-induced experimental colitis in mice. Its anti-inflammatory actions were related to a decrease in the transcription factors NF-κB and STAT3 and the subsequent release of IL-17, IL-6, TNF-α, and COX-2. Moreover, β-carotene exerted an antioxidant activity through an increase in Nrf2 and NADPH:quinone oxidoreductase-1 in the colon tissue [130]. Likewise, the attenuations of NF-κB and STAT3 pathways as well as autophagy inhibition were reported after oral administration of this carotenoid (50mg/kg) in a rat model of LPS-induced intestinal inflammation [131]. In addition, it has been reported that intake of β-carotene (40 and 80 mg/kg) for two weeks inhibited NF-κB pathway activation in a model of weaning-induced intestinal inflammation. The authors proposed a new anti-inflammatory mechanism for this carotenoid involving the modulation of microbiota imbalance as a consequence of weaning in piglets [132]. Regarding liver diseases, β-carotene exhibited a hepatoprotective effect in chemically induced hepatic fibrosis by down-regulating NF-κB and its target gene inducible nitric oxide synthase (iNOS) [133]. In the same line, this carotenoid, administered at a dose of 70 mg/kg every other day or combined with rosuvastatin, attenuated hepatic steatosis and the inflammatory response as well as enhanced the lipid profile in a model of non-alcoholic fatty liver induced by a high-fat diet in rats [134]. 

In relation to cardiovascular disorders, the role of a powder of the microalga *Dunaliella bardawil*, containing 6% β-carotene isomers, was examined in a model of atherosclerosis in apolipoprotein E (apo E)-deficient mice, and fed with a vitamin A-deficient diet. These findings evidenced the formation of atheromas due to lack of vitamin A; nevertheless, β-carotene supplementation decreased levels of plasma cholesterol and prevented atherogenesis [135]. Apo E-/-mice were also used for investigating the actions of dietary β-carotene (800 mg/kg of feed, for 150 days) on angiotensin II-induced chronic renal damage. The results reported a protective effect of this carotenoid by down-regulating the expression of proinflammatory genes related to kidney diseases, including renin 1 and peroxisome proliferator-activated receptor gamma (PPAR-γ) [136].

The beneficial role of β-carotene against skin inflammation has been demonstrated in different animal models. Oral administration of this carotenoid at 0.6 mg/day for 4 weeks attenuated skin inflammatory response in a model of low zinc/magnesium diet-induced atopic dermatitis (AD) in hairless mice. These effects were associated with a down-regulation of the cytokines IL-6, IL-1β, IL-4, and IL-5, a suppression of MMP-9 activity, and an up-regulation of filaggrin levels, a protein involved in skin barrier function [137]. Likewise, the anti-inflammatory activity of β-carotene administered orally (20 mg/kg) for 8 weeks was also reported in a mouse model of oxazolone-induced AD [138]. Furthermore, β-carotene and LUT were evaluated in a mouse model of acute neurogenic inflammation in the ear induced by capsaicin or mustard oil. These carotenoids administered topically at the dose of 100 mg/kg attenuated edema formation; nevertheless, a reduction in myeloperoxidase (MPO) activity and neutrophilic infiltration in the mouse ear was only demonstrated after LUT treatment [139]. 

In relation to central nervous system disorders, the neuroprotective role of this carotenoid was evaluated for the first time in a rat model of acute spinal cord injury. β-Carotene administered intraperitoneally at different doses (10, 20, 40, and 80 mg/kg) suppressed NF-κB pathway activation and exerted a marked antioxidative effect by decreasing ROS, NO, and malondialdehyde (MDA) levels and up-regulating SOD, Nrf2, and HO-1 [140]. In addition, β-carotene has been demonstrated to have protective effects in other inflammatory diseases such gouty arthritis or asthma. In this line, β-carotene administered orally (30 mg/kg) inhibited NLRP3 inflammasome activation in a model of gouty arthritis in mice, as well as suppressed levels of IL-1β in synovial fluid cells isolated from gout patients [127]. Oral treatment with this carotenoid at 30 mg/kg demonstrated a therapeutic effect in a rat model of ovalbumin-induced asthma via reduction in the proinflammatory cytokines IL-β, IL-6, and TNF-α and an increase in the anti-inflammatory cytokines IL-4 and IL-13 [141].

#### 4.1.3. Human Studies

Regarding clinical studies, a randomized, double-blind, and placebo-controlled clinical trial evaluated the role of *Lactobacillus brevis* KB290 and β-carotene in diarrhea-predominant irritable bowel syndrome-like symptoms in healthy people. The intake of this combination for 12 weeks improved the abdominal pain, reduced stool frequency, and decreased colon inflammation through up-regulation of the cytokine IL-10 [142]. Likewise, a double-blind controlled crossover clinical trial in type 2 diabetes mellitus (T2DM) patients demonstrated that supplementation with a β-carotene-fortified symbiotic food (containing *Lactobacillus sporogenes* as probiotic, 0.1 g inulin as prebiotic, and 0.05 g β-carotene) for 6 weeks enhanced insulin metabolism and lipid profile as well as augmented the antioxidant GSH plasma levels [143]. Another study investigated the effects of β-carotene at the doses of 30 and 90 mg/day for 90 days on wrinkles, elasticity, and ultraviolet (UV)-induced DNA damage in healthy females over the age of 50 years. Interestingly, only the lowest dose was effective in preventing and repairing skin photoaging [144]. These data are consistent with previous studies demonstrating the pro-oxidant effects of β-carotene at high doses as it can produce radical ions that themselves may contribute to cell injury [145].

Finally, previous studies have reported that reduced levels of β-carotene can be detected in patients with different inflammatory disorders, including non-alcoholic fatty liver disease [146], chronic obstructive pulmonary disease [147], acute myocardial infarction [148], infection by *H. pylori* [149], and advanced coronary artery disease [150]. These findings support the protective effects of β-carotene through inhibition of the inflammatory processes.

### 4.2. Lutein

#### 4.2.1. In Vitro Studies

The beneficial effects of LUT in ocular disorders have been demonstrated in numerous in vitro studies. Along this line, LUT exhibited a protective role in human retinal pigment epithelial cells (ARPE-19 cells) exposed to different stimuli implicated in age-related macular degeneration pathogenesis (AMD), a severe disease that causes vision loss. The mechanisms underlying these actions were associated with an inhibition of apoptosis, VEGF levels, and oxidative stress markers, as well as prevention of autophagy flux alteration [151]. Similarly, a LUT nanoemulsion improved penetration into ARPE-19 cells and protected cells from H_2_O_2_-induced damage [152]. It has been reported that retinal photo-oxidative damage may lead to inflammation of eyes and AMD-associated lesions. A previous study reported a reduction in proteasome activity in ARPE-19 cells exposed to blue light and that LUT and ZX were able to reverse this effect and regulate inflammation-related genes, such as MCP-1 and IL-8 [153].

Retinal ischemia/reperfusion injury occurs in some eye diseases including glaucoma and diabetic retinopathy. The protective effects of LUT have been reported in a rat Műller cell line exposed to cobalt (II) chloride, a model that mimics the hypoxic/ischemic state. This carotenoid exerted anti-inflammatory effects by reducing NF-κB, IL-1β, and COX-2 levels [154] as well as inhibited apoptosis and autophagy in glial cells [155]. It has been reported that hyperosmoticity of tears induces inflammation and ocular surface damage, playing a main role in dry eye development. In this line, LUT has been shown to be a potential agent for the treatment of dry eye since it suppressed the hyperosmoticity-induced increase in IL-6 through inhibition of NF-*κ*B pathway activation in human corneal epithelial cells [156].

Furthermore, LUT protected a human keratinocyte cell line and primary human keratinocytes from foreskins against UVB-induced damage through an increase in cell viability and proliferation, and reduction in apoptosis [157]. Similarly, LUT pretreatment for 48 h before UVA irradiation preserved tissue architecture in a model of three-dimensional human skin equivalent [158]. The photoprotective effects of this carotenoid were also related to the inhibition of MMP-9 expression and ROS production in UV-irradiated HaCaT [159]. Other papers reported the antioxidant effects of LUT via up-regulation of the Nrf2/HO-1 pathway and its anti-inflammatory actions through inhibition of NF-κB activity in monosodium iodoacetate-induced osteoarthritis in primary chondrocyte cells [160] as well as in LPS-activated microglial cells [161]. In addition, this compound reduced LPS-induced production of TNF-α, IL-6, and IL-1β in peripheral blood mononuclear cells from patients with stable angina [162]. Another action mechanism involved in the anti-inflammatory properties of LUT was related to suppression of the transcription factor AP-1 in LPS-activated macrophages [159]. The antioxidant and anti-inflammatory effects of LUT and its combination with six anthocyanidin glucosides were also evaluated chemically and in Caco-2 cells. LUT alone showed better results than the mixture with the other compounds, demonstrating antioxidant activity through inhibition of liposome peroxidation and anti-inflammatory effects via suppression of the in vitro lipoxygenase-1 activity and reduction in IL-8 and NO levels in Caco-2 cells [163]. 

#### 4.2.2. In Vivo Studies

Like in vitro studies, the protective effects of LUT in eye disorders, such as AMD, diabetic retinopathy, cataract, uveitis, and dry eye syndrome have been previously reported in a number of animal studies. In this respect, LUT and ZX have been evaluated on high-fat diet-induced retinal inflammation in rats since a high-fat intake has been associated with a high incidence of AMD. Data reported that the mix of both carotenoids (100 mg/kg) enhanced metabolic and lipid profile, as well as reduced oxidative stress in the retina by increasing the Nrf2/HO-1 pathway [164]. Light exposure has been reported to be another risk factor for AMD development since it increases the stress in the retinal pigment epithelium. In this line, a LUT-rich marigold extract, composed of 92% LUT and 8% ZX (100 mg/kg), protected the retina from oxidative stress and inflammation in a model of photostressed retina in mice [165]. Regarding diabetic retinopathy, chronic LUT administration (4.2 and 8.4 mg/kg) in the retina of Ins2^Akita/+^ mice, a genetic model of type 1 diabetes, suppressed microglia activation, which is involved in retinal inflammation, and preserved retinal activity [166]. Likewise, LUT supplementation of 0.1% (wt/wt) was reported to have antioxidative effects in the retina in streptozotocin-induced diabetic mice via down-regulation of ROS-mediated extracellular signal-regulated kinase (ERK) activation [167]. In the same experimental model, administration of 0.5 mg/kg LUT or 0.6 and 3 mg/kg ATX exerted antioxidant and anti-inflammatory effects via inhibition of the NF-κB pathway [168]. Furthermore, intraperitoneal administration of micelles containing LUT (1.3 mmol/kg) in combination with three unsaturated fatty acids protected against cataract formation induced by sodium selenite in rat pups. The mechanisms involved in these actions were related to an increase in antioxidant enzymes activity and down-regulation of proinflammatory markers, such as phospholipase A2 (PLA2), COX-2, iNOS, and NF-κB expression [169], as well as regulation of the chaperone function of lens crystallin [170]. The protective effect of LUT at the doses of 125 and 500 mg/kg has also been demonstrated in LPS-induced uveitis in mice through its antioxidant properties, including reduction in NO and MDA levels and an increase in SOD and glutathione peroxidase activities [171]. In the same model, LUT was reported to protect against uveitis via reduction in IL-8 production in uveal melanocytes accompanied by inhibition of JNK1/2 and NF-κB signaling pathways [172]. Furthermore, a recent study has reported the antioxidative and anti-inflammatory effect of a formulation containing LUT/ZX, curcumin, and vitamin D3 in a rat model of benzalkonium chloride-induced dry eye syndrome [173]. 

Regarding cardiovascular diseases, the preventive effects of chronic administration of LUT (25, 50, and 100 mg/kg) on atherosclerosis have been reported in ApoE-deficient mice fed a high-fat diet via an increase in PPAR-α, a marker related to lipid metabolism [174]. Likewise, dietary LUT (0.01 g/100 g diet) improved the lipid profile and reduced oxidative stress and cytokine production in aortas of guinea pigs fed a hypercholesterolemic diet [175]. Later, these authors showed the protective effect of this carotenoid against a high-fat diet-induced hepatic injury by inhibiting NF-κB activity [176].

In relation to the potential role of this corotenoid for pain treatment, this carotenoid has been recently investigated in acute trigeminal inflammatory pain induced by mustard oil injection and chronic trigeminal pain following complete Freund’s adjuvant administration into rat whisker pads. The results in the acute model demonstrated that intraperitoneal administration of LUT (10 mg/kg) suppressed edema thickness and sensitization of nociceptive processing in spinal trigeminal nucleus caudalis (SpVc) and upper cervical (C1) dorsal horn neurons [177]. Similarly, in the chronic model, the carotenoid was able to reduce the hyperalgesia and neuronal hyperexcitability via COX-2 inhibition [178]. Furthermore, LUT attenuated mustard oil-induced acute neurogenic inflammation via suppression of the activation of transient receptor potential ankyrin 1 (TRPA1) on capsaicin- sensitive sensory nerves [139]. This compound has also been reported to have protective effects against thermal injury in remote organs in rats. Oral administration of this compound at the dose of 250 mg/kg for three days attenuated liver and kidney dysfunction and oxidative damage. Moreover, this carotenoid evidenced anti-inflammatory and antiapoptotic properties by reducing TNF-α and caspase-3 expression, respectively, in the liver, kidneys, and lungs [179]. Regarding central nervous system disorders, LUT at the doses of 80 and 160 mg/kg demonstrated anti-inflammatory and antioxidative actions in a model of severe traumatic brain injury via down-regulation of NF-κB and ICAM-1 expression, and up-regulation of Nrf2 and endothelin-1 levels [180]. The antioxidant and anti-inflammatory actions of LUT have been described in other experimental models, such as osteoporosis in ovariectomized rats [181], alcohol-induced hepatic damage [182], and ischemia/reperfusion injury in skeletal muscle [183].

#### 4.2.3. Human Studies

The effects of LUT in AMD have been previously investigated in a variety of clinical studies. One of the largest was the Age-related Eye Disease Study 2 (AREDS2), a double-blind, randomized trial in people at risk of developing late AMD. The results of this study, which evaluated the effects of a formulation of vitamins and zinc, plus LUT/ZX (10mg/2mg), suggest a reduced risk of developing advanced AMD with the consumption of LUT/ZX [184]. These findings were confirmed in a post hoc study evaluating participants enrolled in AREDS 1 and AREDS2 with no late AMD [185]. Likewise, the protective effects of this carotenoid against the development and progression of AMD have been evidenced in other clinical trials by increasing sensitivity of the retina, macular pigment optical density, and visual performance [186,187,188]. Nevertheless, other studies that evaluated the effects of co-administration of LUT and PUFA reported protective actions of this combination in some studies [189] and non-significant effects in others [190]. 

Regarding the photoprotective effects of LUT, a randomized, controlled, double-blind clinical trial in people exposed to UVB/A demonstrated that capsules of LUT (10 mg, twice daily) decreased the skin expression of HO-1, MMP-1, and ICAM-1 [191]. Moreover, oral supplementation with omega-6 and omega-3 fatty acids, ZX, LUT, and vitamin D attenuated sunburn risk in patients with Fitzpatrick skin phototypes I, II, or III [192]. Finally, a recent study confirmed the photoprotective and antiphotoaging effects of a nutritional intervention with different antioxidants, including LUT (3 mg/day), in healthy volunteers [193].

### 4.3. Zeaxanthin

#### 4.3.1. In Vitro Studies

This carotenoid has been shown to have in vitro anti-inflammatory effects in LPS/H_2_O_2_-stimulated human adipose-derived mesenchymal stem cells by reduction in ROS production via down-regulation of the protein kinase C/MAPK/ERK pathway [194]. In addition, ZX prevented oxidative stress in ARPE-19 cells due to PI3K/Akt activation as well induction of phase II enzyme expression via Nrf2 activation [195]. 

#### 4.3.2. In Vivo Studies

The protective role of ZX in ocular diseases has been previously demonstrated in animal models including AMD. In this line, this carotenoid induced an antioxidative response in retinal pigment epithelium, protecting its structure and function in a genetic model of oxidative stress-mediated retinal degeneration in mice [196]. Similarly, this compound attenuated intense light-induced retinal damage by activating Nrf2/HO-1 pathways and suppressing NF-κB expression [197]. Likewise, the neuroprotective effects of LUT/ZX isomers via up-regulation of Nrf2 and down-regulation of NF-κB have been recently reported in a mouse model of traumatic brain injury [198]. On the other hand, ZX was effective in reducing colon inflammation acetic acid-induced ulcerative colitis through an increase in antioxidant defense mechanisms and attenuation of NF-κB levels and the consequent iNOS and COX-2 inhibition [199]. Furthermore, the anti-inflammatory activity of ZX has been evidenced in a model of paw edema in mice [200], as well as in a model of alcoholic fatty liver in rats [201]. This carotenoid also ameliorated diabetes-induced neuroinflammation, improving anxiety and depression [202]. 

#### 4.3.3. Human Studies

As mentioned in the section on LUT, numerous clinical trials have investigated the effects of a combination of LUT and ZX in ocular disorders. In this regard, supplementation with these carotenoids reduced the risk of developing AMD [184,185,203]. Nevertheless, other studies did not report significant changes after LUT and ZX treatment for the prevention of eye diseases or improvement of macular pigments [204]. In relation to dry eye syndrome, a randomized, double-blind, clinical trial reported that oral supplementation with LUT, ZX, curcumin, and vitamin D3 for 8 weeks enhanced dry eye symptoms and attenuated eye inflammation by reducing MMP-9 levels in tears [205].

### 4.4. Astaxanthin

#### 4.4.1. In Vitro Studies

ATX has been shown to have in vitro anti-inflammatory effects in THP-1 macrophages through inhibition of NF-κB activation with the subsequent down-regulation of the proinflammatory markers IL-1β, IL-6, TNF-α, and MMP-2 and 9 [206]. In the same line, this carotenoid suppressed the MAPK signaling pathway, up-regulated the Nrf2 pathway, and increased SIRT-1 activity in ethanol or LPS-induced macrophages from several sources [207,208,209]. In addition, ATX microparticles protected macrophages against radiation-induced damage via suppression of transforming growth factor beta [210]. On the other hand, the neuroprotective role of ATX in LPS-activated BV2 cells has been reported in microglia-mediated inflammation following Alzheimer’s disease through inhibition of MAPK and NF-κB pathway activation [211,212], as well as in particulate matter-stimulated microglial cells [213]. In addition, ATX inactivated STAT3 transcription factor, which led to inhibition of β-secretase activity with the subsequent prevention of amyloid beta accumulation [214]. ATX has also been shown to have antiarthritic properties via reduction of NLRP3 inflammasome stimulation in monosodium urate crystal-activated murine macrophages [215]. Furthermore, ATX protected human primary keratinocytes and HaCaT keratinocytes against UVB-induced damage through reduction of the proinflammatory cytokines IL-8, TNF-α, and IL-1β and the enzymes iNOS and COX-2 [216]. Likewise, the beneficial role of this carotenoid in dry eye treatment was confirmed in human corneal epithelial cells via reduction in TNF-α and IL-1β levels [217]. Finally, the anti-inflammatory and antioxidant effects of this carotenoid have been demonstrated in other in vitro models, including bovine endometritis [218], gastric inflammation by *H. pylori* [219], and osteoporosis [220].

#### 4.4.2. In Vivo Studies

A variety of animal studies have revealed the protective role of ATX against liver inflammation and its progression to cirrhosis and cancer. The mechanisms underlying the anti-inflammatory effects of this carotenoid in the model of non-alcoholic fatty liver were associated with a suppression of endoplasmic reticulum stress and NF-κB [221], a reduction in lipogenic regulator genes [222], and PPAR-α activation [223]. Additionally, the hepatoprotective effects of ATX in liver injury were due to suppression of STAT3 activity in ethanol-induced hepatic damage [224], modulation of gut microbiota [225], inhibition of MAPK pathway activation in acetaminophen-induced hepatic injury [226], and suppression of NF-κB and autophagy in carbon tetrachloride-induced hepatic fibrosis [227] or arsenic-stimulated liver damage [228]. Likewise, dietary ATX (1mg/kg) alleviated high-fructose diet-induced liver inflammation via up-regulation of SIRT-1 and inhibition of NF-κB [229]. Another paper demonstrated that ATX liposomes attenuated LPS-induced acute liver injury in rats, reporting a higher antioxidant and anti-inflammatory activity than free ATX due to an enhancement of its oral bioavailability [230]. In the same line, treatment with ATX (5, 10 and 20 mg/kg) dose-dependently protected against burn-induced acute kidney inflammation through suppression of the TLR4/NF-κB pathway and an increase in HO-1 levels [231]. 

In relation to cardiovascular diseases, it has been recently described that ATX protected mouse heart against LPS-induced cardiac dysfunction by down-regulating MAPK and PI3K/Akt pathways with the consequent apoptosis inhibition [232]. In addition, several animal studies demonstrated the beneficial role of ATX in diabetes mellitus and metabolic syndrome since this carotenoid enhanced the lipid profile and glucose tolerance as well as reduced insulin resistance in a model of chemically induced diabetes [233] and gestational diabetes [234]. Another paper evidenced that PEGylated ATX had a higher antidiabetic effect than free ATX due to an enhancement in oral bioavailability [235]. Additionally, this carotenoid ameliorated diabetic retinopathy in a rat model of streptozotocin-induced diabetes [168,236]. Regarding diabetes-induced brain damage, ATX improved cognitive function through inhibition of NOS activity and up-regulation of the PI3K/Akt pathway [237], as well as activation of the Nrf2/HO-1 pathway in the cerebral cortex and hippocampus [238]. 

ATX has also demonstrated anti-inflammatory effects in central nervous disorders, such as depression; in this line, this compound alleviated depressive-like symptoms in a mouse model of LPS-induced inflammation via attenuation of NF-κB activation and the subsequent suppression of COX-2 and iNOS in the hippocampus and prefrontal cortex [239]. In the same model, a recent study reported that oral treatment with an ATX emulsion to increase its bioavailability improved cognitive function and exhibited anti-inflammatory activity by down-regulating inflammation-related proteins such as COX-2, iNOS, TNF-α, IL-6, and IL-1β and increasing IL-10 levels [240]. Furthermore, ATX was effective in attenuating status epilepticus-induced neuroinflammation in rats by suppressing extracellular ATP levels and the consequent P2X7R inhibition, a microglial receptor involved in inflammation [241]. The neuroprotective effects of this compound were also evidenced in a model of subarachnoid haemorrhage via inhibition of MMP-9 levels and activity [242] and up-regulation of SIRT1 expression [243]. In addition, ATX reduced neuroinflammation in other animal models, such as chronic neuropathic pain [244], spinal cord injury [245,246], Alzheimer’s disease [247], and acute cerebral infarction [248].

Regarding the potential role of ATX for arthritis treatment, this carotenoid protected cartilage against destruction surgically induced by destabilization of the medial meniscus, through Nrf2 activation [249]. In addition, this carotenoid exhibited antiarthritis properties by attenuating chronic inflammatory pain and suppressing proinflammatory and oxidative stress markers in a rat model of arthritis by complete Freund’s adjuvant [250], as well as in monosodium iodoacetate-induced osteoarthritis [251]. ATX also attenuated inflammation in a model of gouty arthritis in rats [215] and in different animal models of gastrointestinal inflammation. In this regard, it has been recently demonstrated that dietary ATX (0.005%) ameliorated oxidative stress, interferon gamma (IFN-γ) levels, and the oncogenes c-myc and cyclin D1 in a mouse model of *H. pylori*-associated gastritis, suggesting the chemopreventive role of this carotenoid in *H. pylori*-induced carcinogenesis [252]. Additionally, ATX administered orally (100 mg/kg) attenuated ochratoxin A-induced cecum inflammation due to suppression of TLR4 and its downstream protein Myd88, as well as inhibition of NF-κB and the subsequent release of TNF-α and IFN-γ [253]. Similarly, ATX supplementation revealed a protective role in DSS-induced ulcerative colitis in mice through down-regulation of NF-κB-induced COX-2 and iNOS expression [254]. Similar findings were reported when ATX was administered to obese mice, suppressing the development of azoxymethane-induced colonic premalignant lesions [255]. Additionally, this carotenoid improved acute pancreatitis in mice via suppression of JAK/STAT3 activity [256].

The beneficial role of ATX in pulmonary disorders has also been reported in different in vivo models. At this respect, this compound exhibited antiasthmatic effects in ovalbumin-induced asthma in mice due to modulation of Th1 and Th2 cytokine profiles [257]. Furthermore, ATX inhibited inflammatory and oxidative response in acute lung injury via attenuation of oxidative/nitrosative stress markers, apoptosis, and NF-κB expression [258] as well as an increase in the Nrf2/HO-1 signaling pathway [259]. As regards skin diseases, it has been reported that this carotenoid administered topically on the ear or back skin of mice alleviated hyperkeratosis and inflammatory response in a model of phthalic anhydride-induced atopic dermatitis. These actions were related to a down-regulation of NF-κB and its proinflammatory target genes iNOS and COX-2 [260,261]. In the same model, ATX-loaded liposomes were more effective than free ATX in alleviating skin inflammation due to inhibition of oxidative stress and STAT3 and NF-κB signaling pathways as well as a reduction of IgE, a marker of allergic inflammation [262]. Likewise, oral treatment with ATX enhanced atopic dermatitis-induced pruritus and inflammation, evidenced by an inhibition of proinflammatory cytokines and L-histidine decarboxylase levels [263]. Moreover, ATX protected mouse skin against burn injury as well as corneal epithelium against UV-induced keratitis by suppressing proinflammatory and oxidative markers and apoptosis [264,265]. On the other hand, ATX has been shown to have anti-inflammatory effects in a mouse model of hyperosmoticity-induced dry eye due to suppression of TNF-α and IL-1β, as well as down-regulation of high-mobility group box 1, a proinflammatory marker involved in ocular damage [217]. 

#### 4.4.3. Human Studies

Regarding human studies, the photoprotective and antiaging effects of ATX have been demonstrated in a randomized and double-blind study in healthy women exposed to UVB and receiving ATX capsules at 6 or 12 mg/day for 16 weeks. At the end of the study, the carotenoid was effective in attenuating wrinkle formation and improving skin elasticity [266]. Similar results were detected in another clinical trial in participants treated with ATX capsules at 4 mg for 9 weeks [267]. Additionally, an ATX supplement (6 mg/day) for 12 weeks increased cognitive function in patients with mild cognitive impairment [268], and this treatment for 4 weeks alleviated mental and physical fatigue in healthy volunteers [269]. Furthermore, administration of ATX at 8 mg/day for 8 weeks improved the lipid profile and reduced blood pressure in patients with T2DM [270]. Likewise, the beneficial effects of the same dose of ATX in T2DM have been recently reported in a randomized, double-masked clinical trial through reduction in IL-6 and MDA levels as well as down-regulation of microRNA 146a, a proinflammatory marker whose deregulation has been implicated in diabetes pathogenesis and complications [271]. 

### 4.5. Fucoxanthin

#### 4.5.1. In Vitro Studies

The carotenoid FX has been shown to have marked anti-inflammatory effects in different in vitro experimental models. In this line, FX suppressed COX-2 and iNOS expression and the consequent production of PGE_2_ and NO, respectively, as well as reduced TNF-α, IL-1β, and IL-6 levels via inhibition of NF-κB and MAPK pathways in LPS-stimulated RAW 264.7 macrophages [272,273]. A recent study reported that this carotenoid attenuated the palmitate-induced inflammatory response in RAW 264.7 macrophages by improving lipid metabolism and mitochondrial dysfunction. Additionally, this compound blocked the expression gene of M1 markers (IL-6, IL-1β, TNF-α, and Nlrp3) and up-regulated the expression of the M2 marker Tgfβ1, thus suppressing macrophage-induced inflammation [274]. Another study by our group confirmed the anti-inflammatory activity of FX due to a reduction in TNF-α levels in LPS-activated THP-1 macrophages and IL-6 and IL-8 production in TNF-α-stimulated HaCaT keratinocytes, an in vitro model of psoriasis [275].

In relation to neurodegenerative diseases, FX has been demonstrated to have neuroprotective effects in amyloid-β_42_-stimulated BV2 microglia cells [276], as well as in LPS-activated BV2 cells via inhibition of Akt/NF-κB and MAPK/AP-1 pathways and activation of the Nrf2/HO-1 pathway [277]. Likewise, the antifibrotic effect of FX has also been reported in TGF-β1-stimulated human pulmonary fibroblasts via suppression of MAPK, PI3K/Akt, and Smad2/Smad3 pathways [278]. On the other hand, our group has previously shown that FX protected HaCaT cells against UVB irradiation via attenuation of ROS and IL-6 production [275]. Interestingly, the combination of FX and the polyphenol rosmarinic acid down-regulated inflammasome-related proteins such as NLRP3, ASC, and caspase-1 and up-regulated the Nrf2/HO-1 pathway in UVB-irradiated HaCaT keratinocytes [279]. In the same line, a sunscreen containing FX 0.5 (*w/v*) revealed photoprotective properties in UVA-stimulated reconstructed human skin (RHS) via reduction in ROS production [280]. These authors also reported that this carotenoid administered topically in RHS attenuated ethanol-induced skin inflammation through an increase in filaggrin expression [281]. As regards ocular diseases, FX protected ARPE-19 cells against high glucose-induced diabetes retinopathy in ARPE-19 cells via up-regulation of Nrf2 and reduction in apoptosis [282].

Furthermore, the potential therapeutic effect of FX has been reported in LPS-stimulated Caco-2 cells, an in vitro intestinal inflammation model. This carotenoid improved the intestinal epithelial barrier and reduced IL-1β and TNF-α levels and increased the anti-inflammatory cytokine IL-10 [283]. In relation to metabolic disorders, FX inhibited lipid accumulation and ROS production by modulating adipogenic and lipogenic mediators and increasing antioxidant enzymes in adipocytes, demonstrating interesting antiobesity properties [284,285,286]. According with these findings, FX stimulated lipolysis and supressed lipogenesis in oleic acid-induced hepatocytes, a fatty liver cell model, through activation of the SIRT1/AMP-activated protein kinase (AMPK) pathway [287]. In the same line, antiobesity activity has also been reported after fucoxanthinol treatment, a metabolite of FX, in TNF-α-stimulated adipocytes by reducing the levels of adipocytokines, such as IL-6 and MCP-1, and in palmitic acid-stimulated RAW264.7 cells by inhibiting TNF-α production [286]. These effects were confirmed in a model of low-grade chronic inflammation, consisting of a co-culture of adipocytes and macrophages, demonstrating that this compound ameliorated inflammation in adipose tissue [284].

#### 4.5.2. In Vivo Studies

The anti-inflammatory effects of FX have been demonstrated in a variety of animal models. In terms of skin disorders, a study by our group in the 12-O-tetradecanoylphorbol-13-acetate (TPA) model, which mimics psoriatic markers in mouse dorsal skin, evidenced that topical administration of an FX cream improved hyperplasia via suppression of MPO activity and COX-2 expression. Additionally, this preparation protected mouse skin against UVB-induced acute erythema due to inhibition of COX-2 and iNOS expression and up-regulation of the Nrf2/HO-1 pathway [275]. Furthermore, FX-containing Vaseline improved AD skin symptoms in the Nc/Nga mouse model through an increase in regulatory innate lymphoid cell-released IL-2 and IL-10 [288]. This carotenoid (4 and 8mg/kg) also suppressed inflammation in the mouse model of carrageenan-induced paw edema due to inhibition of MAPK, NF-κB, and protein kinase B/Akt pathways [289]. Regarding colon inflammation, treatment with FX at 50 and 100 mg/kg ameliorated DSS-induced acute colitis in mice by down-regulation of the NF-κB/COX-2/PGE2 pathway [290]. Similar results were reported after FX administration in a rat model of carrageenan/kaolin-induced arthritis [291]. According to these findings, this carotenoid (200 mg/kg) improved LPS-induced depressive and anxiety-like behaviors via suppression of NF-κB and its proinflammatory target genes iNOS, COX-2, IL-1β, IL-6, and TNF-α, as well as activation of AMPK [292]. In addition, FX treatment demonstrated antifibrotic actions in bleomycin-induced pulmonary fibrosis in mice [293], as well as antiasthmatic effects in an ovalbumin-induced asthma mouse model [294,295].

The therapeutic effects of FX in metabolic diseases have been demonstrated in different animal models of obesity. In this respect, oral administration of FX (0.2, 0.4, and 0.6 %) was effective in reducing inflammation through reduction in IL-1β, TNF-α, iNOS, and COX-2 in a model of high-fat diet-induced obesity [296]. Later, this effect was confirmed in the same model after administration of FX at the dose of 1 mg/kg, showing that this carotenoid improved the lipid profile and insulin resistance and decreased blood pressure. Furthermore, FX up-regulated the anti-inflammatory cytokine adiponectin and inhibited leptin expression, a hormone associated with obesity [297,298]. In the same model, FX demonstrated antiobesity properties via modulation of gut microbiota composition [297,298] and stimulation of the Nrf2/NQO1 pathway [299]. Likewise, FX supplementation (0.1 and 0.2%) prevented obesity development and reduced hyperglycemia in diabetic/obese KK-Ay mice, by supressing MCP-1 and TNF-α, which are involved in insulin resistance [286]. Moreover, an extract from *Laminaria japonica* with a high FX content enhanced insulin sensitivity and reduced lipidic peroxidation in a model of streptozotocin- and nicotinamide-induced diabetes [300]. In relation to hepatic disorders, the protective effect of dietary FX (0.2%) has been reported in a mouse model of non-alcoholic fatty liver induced by a high-fat diet via suppression of hepatic fat accumulation and MCP-1 expression [301]. In the same line, FX treatment (10, 20 or 40mg/kg) protected against alcohol-induced liver damage via up-regulation of Nrf2 and suppression of the TLR4-mediated NF-κB pathway [302]. 

#### 4.5.3. Human Studies

Regarding human studies, a randomized controlled clinical trial has recently reported the protective effect of a combination of fucoidan, a polysaccharide mainly derived from brown seaweed (825 mg), and FX (825 mg), twice a day for 24 weeks in non-alcoholic fatty liver disease patients. The results demonstrated that this treatment improved the lipid profile and reduced hepatic steatosis and inflammation by inhibiting plasma levels of IL-6 and IFN-γ [303].

### 4.6. β-Cryptoxanthin

#### 4.6.1. In Vivo Studies

The beneficial role of BCX has been reported in different animal studies. In this line, this carotenoid administered orally (2 and 4 mg/kg) protected the retina against light-induced damage through an increase in antioxidant status as well as a reduction in NF-κB levels and the subsequent production of IL-1β and IL-6 [304]. As regards metabolic disorders, the antiobesity properties of dietary BCX for 12 weeks have been reported in a mouse model of high-fat diet-induced insulin resistance. The mechanisms underlying this effect were associated with a down-regulation of NF-κB expression and up-regulation of the Nrf2/HO-1 pathway [305], as well as modulation of the M1/M2 status, resulting in an increase in the M2 macrophage population [306]. Likewise, the cardioprotective effect of this carotenoid has been recently reported in a rat model of ischemia/reperfusion-induced myocardial injury by down-regulating the NF-κB pathway [307]. In addition, BCX attenuated the development of surgically induced osteoarthritis by inhibiting proinflammatory cytokine levels [308] as well as ameliorated cigarette smoke-induced lung inflammatory response and squamous metaplasia via reduction in the NF-κB/TNF-α pathway [309].

#### 4.6.2. Human Studies

Regarding human studies, a randomized, double-masked, and placebo-controlled clinical trial enrolling subjects suffering non-alcoholic fatty liver disease demonstrated that a BCX capsule for 12 weeks attenuated oxidative stress and inflammatory processes via reduction in MDA and IL-6 serum levels, respectively [310].

**Table 1:** Microalgal carotenoids and their described activities in inflammation and cancer.

Carotenoid	Source	Bioactivity	References
β-Carotene	*Dunaliella salina* *Chlamydomonas reinhardtii* *Isochrysis galbana* *Tetraselmis suecica*	**Inflammation**	
		Colitis	[130,131,132]
		Hepatic fibrosis	[133]
		Non-alcoholic fatty liver	[134]
		Atherosclerosis	[135,136]
		Atopic dermatitis	[137,138]
		Neurogenic inflammation	[139]
		Acute spinal cord injury	[140]
		Arthritis	[127]
		Asthma	[141]
		Irritable bowel syndrome	[142]
		Type 2 diabetes mellitus	[143]
		Skin photoaging	[144,145]
		**Cancer**	
		Colon cancer	[311,312]
		Liver cancer	[313,314]
		Gastric cancer	[315,316]
		Esophageal squamous cell	[317,318]
		carcinoma	
		Prostate cancer	[319]
		Neuroblastoma	[320]
		Breast cancer	[321,322,323]
		Pancreatic cancer	[324]
		Non-Hodgkin lymphoma	[325]
Lutein	*Chlorella sorokiniana* *Chromochloris zoofingiensis* *Auxenochlorella protothecoides* *Dunaliella salina* *Chlamydomonas* sp. *Tetraselmis suecica*	**Inflammation**	
		Age-related macular	[165,184,185,186,187,188]
		degeneration	
		Diabetic retinopathy	[166,167,168]
		Uveitis	[171,172]
		Dry eye syndrome	[173]
		Atherosclerosis	[174,175]
		Hepatic injury	[176]
		Pain	[139,177,178,179]
		Osteoporosis	[181]
		Alcohol-induced hepatic	[182]
		damage	
		Ischemia/Reperfusion	[183]
		Photoprotective/	[191,192,193]
		Antiaging effects	
		**Cancer**	
		Colon cancer	[326,327]
		Hepatocellular carcinoma	[328]
		Breast cancer	[329,330]
		Bladder cancer	[331]
		Renal cell carcinoma	[332]
		Neck cancer	[333]
		Non-Hodgkin lymphoma	[325]
		Pharyngeal cancer	[334]
		Esophageal cancer	[318]
		Pancreatic cancer	[335]
Zeaxanthin	*Synechocystis* sp. *Microcystis aeruginosa* *Nannochloropsis oculata* *Chloroidium saccharophilum* *Dunaliella* sp. *Porphyridium purpureum* *Heterosigma akashiwo*	**Inflammation**	
		Age-related macular	[184,185,196,336]
		degeneration	
		Traumatic brain injury	[198]
		Colitis	[199]
		Edema	[200]
		Alcoholic fatty liver	[201]
		Depression/Anxiety	[202]
		Eye dry syndrome	[205]
		**Cancer**	
		Uveal melanoma	[337]
		Pancreatic cancer	[338]
		Ovarian cancer	[339]
		Bladder cancer	[331]
		Breast cancer	[330]
		Non-Hodgkin lymphoma	[325]
		Pharyngeal cancer	[334]
		Esophageal cancer	[318]
		Colon cancer	[340]
		Pancreatic cancer	[335]
Astaxanthin	*Haematococcus lacustris* *Chromochloris zofingiensis* *Chlorococcum* sp. *Dunaliella salina* *Tetraselmis suecica*	**Inflammation**	
		Non-alcoholic fatty liver	[221,222,223]
		Liver inflammation	[224,225,226,227,228,229,230]
		Kidney inflammation	[231]
		Cardiac dysfunction	[232]
		Diabetes mellitus	[233,234,235,270,271]
		Diabetes-related disorders	[168,236,237,238]
		Depression	[239,240,341]
		Epilepsy-induced	[241,242,243]
		neuroinflammation	
		Acute cerebral infarction	[248]
		Arthritis	[215,249,250,251,342]
		Colitis	[254,255]
		Asthma	[257]
		Acute lung injury	[258,259,343]
		Contact dermatitis	[344]
		Atopic dermatitis	[260,261,262,263]
		Dry eye	[217]
		Photoprotective/	[266]
		Antiaging effects	
		Cognitive function	[268]
		**Cancer**	
		Hepatocellular carcinoma	[345,346,347]
		Mammary tumor	[348]
		Colon cancer	[349]
		Esophageal cancer	[350]
		Oral cancer	[351,352]
		Prostate cancer	[353]
		Lung metastatic melanoma	[354]
Fucoxanthin	*Isochrysis* sp. *Odontella aurita* *Chaetoceros neogracilis* *Chrysotila carterae Phaeodactylum* *tricornutum* *Pavlova* sp.	**Inflammation**	
		Psoriasis/Acute erythema	[275]
		Atopic dermatitis	[288]
		Edema	[289]
		Colitis	[290]
		Arthritis	[291]
		Depression/Anxiety	[292]
		Lung injury	[278,293]
		Asthma	[294,295]
		Obesity	[296,297,298,299]
		Diabetes	[300]
		Non-alcoholic fatty liver	[301,302,303]
		**Cancer**	
		Colon cancer	[355,356,357,358,359]
		Lung cancer	[360,361,362]
		Hepatocellular carcinoma	[363]
		Glioblastoma	[364]
		Cervical cancer	[365]
		Melanoma	[366]
		Sarcoma	[367]
β-Cryptoxanthin	*Phaeodactylum tricornutum* *Auxenochlorella pyrenoidosa* *Porphyridium purpureum*	**Inflammation**	
		Obesity	[305,306]
		Ischemia/Reperfusion	[307]
		Osteoarthritis	[308]
		Lung inflammation	[309]
		Non-alcoholic fatty liver	[310]
		**Cancer**	
		Gastric cancer	[368,369]
		Hepatocellular carcinoma	[370]
		Lung cancer	[371,372,373]
		Non-Hodgkin lymphoma	[374]
		Colon cancer	[375]
		Head/Neck cancer	[333]
		Breast cancer	[376]
		Renal cell carcinoma	[377]

## 5. Anticancer Activity of Carotenoids

### 5.1. β-Carotene

#### 5.1.1. In Vitro Studies

Numerous in vitro studies have reported the anticancer activity of β-carotene in gastrointestinal cancers. In this line, this carotenoid inhibited the cell growth of the colorectal cancer cells HT-29 [378] and Caco-2 [379]. In addition, β-carotene exhibited anticancer properties via suppression of M2 macrophage polarization, which has a main role in promoting tumor progression and metastasis, as well as reduction in the migration and invasion of HCT116 colon cancer cells [311]. Another paper demonstrated that the molecular mechanisms underlying the anti-colon cancer effects of β-carotene were related to regulation of epigenetic modifications, including an increase in histone acetylation and reduction in DNA methylation [340]. 

Moreover, β-carotene was reported to act as a proapoptotic agent in gastric cancer cells through reduction in the expression and activity of Ku proteins, which are involved in the repair process of damaged DNA [380]. Furthermore, this carotenoid inhibited proliferation of *H. pylori*-infected gastric adenocarcinoma cells through suppression of NF-κB activation, which in turn down-regulated tumor necrosis factor receptor-associated factor 1 (TRAF1) and TRAF2 expression [381], as well as inhibition of β-catenin signaling and oncogene expression [382]. As regards esophagus cancer, β-carotene has been reported to suppress the growth of a human esophageal squamous cell carcinoma cell line and induce apoptosis via down-regulation of NF-κB/Akt pathway activation and caveolin-1 protein expression [383]. Later, these authors demonstrated a greater antiproliferative effect of β-carotene when it was combined with 5-fluorouracil [317]. Other mechanisms underlying the anticancer effects of this carotenoid in esophageal squamous carcinoma cells include up-regulation of PPAR-γ and down-regulation of cyclin D1 and COX-2 expression [384]. Likewise, β-carotene, in combination with α-carotene, demonstrated a strong antiproliferative activity as well as a reduction in DNA synthesis in esophageal cancer cells [385]. In relation to hepatic cancer, a mixture of different carotenoids, including α- and β-carotene, lycopene, LUT, and BCX, evidenced a higher antimetastatic activity than individual carotenoids in human hepatocarcinoma SK-Hep-1 cells [386]. In addition, β-carotene at a plasma peak concentration exhibited genotoxic and cytotoxic antitumor activity in HepG2 cells [387]. In this cell line, *Dunaliella salina* (as *Dunaliella bardawil*) (Chlorophyta) biomass-loaded nanoparticles, whose majority components are β-carotene, LUT, ZX, CX, phytoene, and phytofluene, were effective in inhibiting cell proliferation and inducing apoptosis [388]. 

The antiproliferative and proapoptotic actions of β-carotene have also been reported in human cervical cancer cells, hepatoma cells, and breast cancer cells, via inhibition of human calcium/calmodulin-dependent protein kinase IV activity [389], as well as in adrenocorticotropic hormone-secreting pituitary adenoma AtT-20 cells [390]. This carotenoid also suppressed cell proliferation in leukemia K562 cells through an increase in PPAR-γ expression [391], as well as increased the growth inhibitory effect of the anticancer drug trichostatin A in the lung carcinoma cell line A549 [392]. β-Carotene has also been shown to have an antiproliferative effect in human breast adenocarcinoma cells via induction of apoptosis and cell cycle arrest [393], as well as suppression of PI3K/Akt and ERK signaling pathways [394]. Similarly, the combination of a low-dose doxorrubicin treatment with several carotenoids, such as β-carotene, LUT, ATX, or FX, was reported to have a cell growth inhibitory effect and a proapoptotic effect in breast cancer cells [395]. Similar results were reported after treatment with β-carotene-loaded solid lipid nanoparticles [396]. 

#### 5.1.2. In Vivo Studies

In relation to in vivo studies, β-carotene at the doses of 5 and 15 mg/kg twice weekly for 11 weeks was demonstrated to be effective in the reduction of tumor growth in a model of colitis-associated colon cancer in mice via suppression of M2 macrophage polarization [311]. Similarly, this carotenoid administered orally (20, 40, and 60 mg/kg) for 30 days decreased tumor weight and size in a rat model of H22 cell-induced liver cancer [313]. In addition, the chemopreventive role of β-carotene in gastric cancer was demonstrated in a model of tobacco smoke-exposed mice. This carotenoid prevented epithelial–mesenchymal transition, which is involved in the gastric cancer development, through inhibition of Notch pathway activation [315]. Another paper evidenced that β-carotene in combination with 5-fluorouracil suppressed tumor growth and induced apoptosis in a mouse model of Eca109 cells (an esophageal squamous cell carcinoma cell line) [317]. Similarly, this carotenoid administered at the dose of 16 mg/kg twice a week for 7 weeks showed antiproliferative effects in a xenograft model of prostate cancer [319].

As regards extracranial solid tumors, oral pretreatment with β-carotene reduced tumor growth in a neuroblastoma model, as well as induced cell differentiation and inhibited cancer cell stemness via down-regulation of different cancer stem cell markers [320]. In the same model, these authors confirmed the anticarcinogenic effects of this carotenoid on the murine liver microenvironment of a metastatic neuroblastoma through suppression of proliferation and angiogenesis, as well as inhibition of apoptosis by up-regulating of Bcl-2 and down-regulating Bax protein [397]. Finally, β-carotene-loaded lipid polymer hybrid or zein nanoparticles were shown to reduce tumor growth in a model of chemically induced breast cancer in rats and this effect was enhanced when the carotenoid was co-administered with methotrexate [321,322].

#### 5.1.3. Human Studies

Previous human studies have reported that reduced levels of β-carotene can be detected in patients with different cancers, including oral cancer [398], breast cancer [399], prostate cancer [400], pancreatic cancer [338], and malignant pleural mesothelioma [401]. Moreover, numerous epidemiological studies have indicated that dietary intakes of β-carotene, obtained from fruits and vegetables, may reduce cancer mortality [402] and protect against the development of some gastrointestinal cancers, such as esophageal cancer [318], gastric cancer [316], colon cancer [312], pancreatic cancer, and hepatocellular carcinoma [314,324]. Likewise, consumption of this carotenoid exerted a chemopreventive effect against the development of breast cancer [323,403], lung cancer [404], head and neck cancer [333], and non-Hodgkin lymphoma [325]. However, other human studies have reported contradictory results since β-carotene supplementation was associated with higher risk of developing cancer, such as breast cancer [405] and lung cancer in smokers [406]. These effects may be explained due to the antioxidant properties of this carotenoid, which would lead to a reduction in ROS production with the consequent apoptosis inhibition. In conclusion, further studies for β-carotene are needed to assess this potential association.

### 5.2. Lutein

#### 5.2.1. In Vitro Studies

Several in vitro studies have reported the anticancer properties of LUT in breast cancer. In this regard, LUT inhibited cell growth and induced apoptosis in two breast cancer lines, the non-invasive MCF-7 and invasive MDA-MB-231 cells. The mechanisms underlying these effects were associated with an inhibition of the transcription factor Nrf2 and its target genes SOD-2 and HO-1, as well as a down-regulation of cell survival markers such as pAkt, pERK, and NF-κB [407]. Other mechanisms involved in the anti-breast cancer effects of this carotenoid include inhibition of glycolysis [408], suppression of cell cycle progression, stimulation of p53 signaling, and an increase in cellular heat shock protein 60 expression [409]. Moreover, LUT inhibited cell invasion and migration under hypoxic conditions through down-regulation of the transcription factor hairy and enhancer of split-1 (HES1) in MCF-7 and MDA-MB-231 cells [410]. In the same cell lines, the epoxide form of LUT exhibited higher cytotoxic and proapoptotic activity than LUT [411]. Likewise, LUT-loaded nanoparticles exhibited an antiproliferative effect in MCF-7 cells [412].

The antiproliferative and proapoptotic actions of LUT have also been described in other cancer cell lines, including sarcoma S180 cells [413], colon adenocarcinoma cells [414], prostate cancer (PC-3) cells [415], A549 lung cancer cells [416], and lymphoid leukaemia cell lines [417].

#### 5.2.2. In Vivo Studies

As regards preclinical animal studies, the chemoprotective effect of dietary LUT (0.002%) administered either 8 weeks before or after the induction of neoplasia was reported in dimethylhydrazine-induced colon cancer. This carotenoid reduced tumor incidence and down-regulated some proteins involved in cell proliferation, such as K-ras, Akt/protein kinase B, and β-catenin [326]. Moreover, LUT (50 and 250 mg/kg) effectively inhibited carcinogenesis in a model of N-nitrosodiethylamine-induced hepatocellular carcinoma in rats via suppression of cytochrome P450 phase I enzyme activity and induction of detoxifying phase II enzymes [328]. More recently, it has been reported that daily administration of LUT (50 mg/kg) for 30 days inhibited tumor growth in a murine breast cancer model induced by injection of 4T1 cells [329]. Similar results were found when this carotenoid (40 mg/kg) was administered to mice inoculated with sarcoma S180 cells; interestingly, the growth inhibitory effect was higher when this carotenoid was co-administered with doxorubicin [413]. 

#### 5.2.3. Human Studies

The protective effects of dietary LUT and ZX in the prevention of cancer have been revealed in human epidemiological studies, which reported that consumption of these carotenoids reduced the risk of different cancers, such as bladder cancer [331], breast cancer [330], renal cell carcinoma [332], head and neck cancer [333], and non-Hodgkin lymphoma [325]. Similarly, it has been reported that intake of LUT and ZX was inversely related with a decreased risk of gastrointestinal cancers, including oral and pharyngeal cancer [334], esophageal cancer [318], colon cancer [327], and pancreatic cancer [335].

### 5.3. Zeaxanthin

#### 5.3.1. In Vitro and Animal Studies

The in vitro anticancer effects of ZX have been recently reported in HT-29 cells [378,414] as well as in several human gastric cancer cells. This carotenoid exhibited cytotoxic effects and induced G2/M cell cycle and apoptosis in gastric cancer cells by up-regulating several proapoptotic factors, such as Bax, and down-regulating some antiapoptotic proteins, such as Bcl-2, among others. Moreover, these authors suggested that LUT-induced ROS production may induce regulation of the MAPK signaling pathway and, consequently, activate apoptosis [418]. A bioguided study of the microalga *Cyanophora paradoxa* (Glaucophyta) reported a marked antiproliferative activity of different fractions rich in ZX and BCX in A-2058 melanoma cells [419]. Other papers evidenced the potential of ZX as an antimelanoma agent since this carotenoid induced apoptosis of human uveal melanoma cells [420], as well as suppressed platelet-derived growth factor and melanoma cell-induced fibroblast migration [421]. A preclinical study in mice reported that intravitreal injection of ZX markedly supressed the tumor growth and invasion in a model of human uveal melanoma induced by injection of C918 cells [337]. 

#### 5.3.2. Human Studies

As regards human studies, in the section on LUT the chemopreventive effects of intake of LUT and ZX in the development of many tumors have already been mentioned. In addition, other studies have described an inverse association between low plasma levels of ZX and increased risk of pancreatic cancer [338] and ovarian cancer [339]. 

### 5.4. Astaxanthin

#### 5.4.1. In Vitro Studies

Several studies have reported the anticancer activity of this red pigment carotenoid. In this regard, a study evaluated the role of ATX on pontin, a conserved ATPase of the AAA+ (ATPases associated with various cellular activities) superfamily overexpressed in many cancers. This carotenoid modulated the expression of pontin, which led to a reduction in the proliferation and migration of breast cancer cells when compared to normal breast cells [422]. Recently, the role of ATX has been reported as a novel metastasis inhibitor on the human breast cell line T47D through activation of different tumor metastasis suppressors such as maspin, Kai1, breast cancer metastasis suppressor 1, and mitogen-activated protein kinase kinase 4 [423]. In addition, the cytotoxic effect of ATX against ovarian carcinoma cells via promotion of apoptosis and inactivation of the NF-κB signaling pathway has recently been reported [424]. 

ATX has also shown antiproliferative effects in leukemia K562 cells by PPAR-γ inhibition [425]. In addition, this compound may induce G0/G1 or G2/M cell cycle arrest, modulate epigenetic alterations (e.g., cell cycle regulator genes or growth factors), and inhibit angiogenesis and metastasis in different cancer cell lines including glioblastoma [426,427,428]. These mechanisms were also observed in murine hepatoma cells H22 [429] and in several human adenocarcinoma gastric cell lines such as AGS, KATO-III, MKN-45, and SNU-1 [430]. Additionally, this carotenoid induced mitochondrial membrane damage, decreasing its transmembrane potential and the function of electron transport, which promoted the expression of proapoptotic proteins in rat hepatocellular carcinoma cells [431]. Furthermore, ATX evidenced protective effects against the gastric disease associated with *H. pylori* infection by promoting autophagy through AMPK pathway activation and reducing the oxidative stress in the gastric adenocarcinoma cell line AGS [432]. 

Regarding colon cancer, ATX has been reported to inhibit cancer cell growth not only by arresting cell cycle progression but also by promoting apoptosis via an increase in caspase 3 expression in colon cancer cells [433]. Additionally, this carotenoid was able to promote the expression of Bax, p53, p21, and p27 and the phosphorylation of p38, JNK, and ERK1/2. Moreover, cyclin D1 and Bcl-2 expression and Akt phosphorylation were found to be significantly decreased by ATX treatment, suggesting a protective role against colon cancer cells [434], MCF-7 breast cancer cells [435], and glioblastoma [426]. It is worth highlighting that the three stereoisomers of ATX (*S*, *R*, and a mixture of *S*:meso:*R*) exhibited antiproliferative activity in HCT116 and HT29 colon cancer cells via apoptosis induction and cell cycle arrest; however, terminal ring structures were not involved in these antitumor effects since no significant differences were detected between the three stereoisomers [436]. Concerning skin cancer, ATX has been shown to decrease tyrosinase activity on human dermal fibroblasts, which can lead to a malignant transformation of normal melanocytes and promote skin cancer [437]. 

#### 5.4.2. In Vivo Studies

Previous in vivo studies have reported the anticancer activity of ATX in gastrointestinal cancers. In this line, the chemoprotective effect of ATX administered orally (15 mg/kg) for 16 weeks was reported in dimethylhydrazine-induced colon cancer in rats through apoptosis induction via down-regulation of ERK-2, NF-κB, and COX-2 [349]. Similar results were demonstrated after ATX treatment (200 ppm in the diet) in the experimental model of colitis-associated colon cancer induced by azoxymethane (AOM)/DSS in mice [254]. Likewise, dietary intake of ATX at the same dose supressed AOM-induced colonic premalignant lesion development in mice via attenuation of oxidative stress markers and inactivation of NF-κB [255]. Regarding oral cancer, ATX effectively inhibited carcinogenesis in 7,12-dimethylbenz[a]anthracene (DMBA)-induced buccal pouch cancer in hamsters via down-regulation of NF-κB and Wnt/β-catenin signaling pathways. In addition, this carotenoid induced caspase-mediated mitochondrial apoptosis through attenuation of the antiapoptotic Bcl-2, p-Bad, and surviving expression and up-regulation of the proapoptotic proteins Bax and Bad [351]. In the same model, an ATX-enriched diet (15 mg/kg) suppressed tumor progression via inhibition of the JAK/STAT3 signaling pathway and its downstream targets cyclin D1, MMP-2 and -9, and VEGF, preventing cell proliferation and invasion and, consequently, regulating tumor microvascular density [352]. In addition, ATX supplementation at the dose of 25 mg/kg effectively suppressed tumorigenesis in a rat model of N-nitrosomethylbenzylamine-induced esophageal cancer by down-regulating NF-κB and its target gene COX-2 [350]. Likewise, the chemopreventive effects of dietary ATX (200 ppm) were also reported in diethylnitrosamine (DEN)-induced hepatic cancer in obese mice via attenuation of oxidative stress and an increase in serum adiponectin levels [347].

Regarding skin cancer, the chemopreventive role of ATX (200 μg/kg) was demonstrated in a rat model of UV-DMBA-induced skin tumorigenesis through inhibition of tyrosinase activity and modulation of oxidative stress [438]. In the same line, nitroastaxanthin, the main reaction product of ATX with peroxynitrite, reduced the number of papillomas in a two-stage carcinogensis model on mouse skin initiated by DMBA and promoted by TPA [439]. Moreover, an oral nanoemulsion containing 15 mg/kg of ATX has been found to suppress lung metastatic melanoma by apoptosis activation via down-regulation of Bcl-2, ERK, and NF-κB in B16F10 cell-injected mice [354]. Likewise, in a xenograft model induced by human mammary tumor cells, a diet containing 0.005% ATX for 8 weeks reduced tumor growth and regulated immune response when this carotenoid was administered before tumor initiation, increasing NK cell populations and plasma IFN-γ levels. However, mice fed ATX after tumor initiation exhibited a faster tumor growth and increased plasma levels of IL-6 and TNF-α, showing the importance of a good antioxidant status prior to tumor initiation [348]. Additionally, this carotenoid administered orally at the dose of 100 mg/kg suppressed tumor growth and induced apoptosis via caspase-3 activation in a xenograft model of prostate cancer in nude mice [353]. 

#### 5.4.3. Human Studies

ATX is considered as a phytonutrient with strong anti-inflammatory and antioxidant activity. Moreover, the European Food Safety Authority (EFSA) recently reported that the intake of 8 mg ATX per day is safe [440], although no toxic effect has been shown with an exceeded EFSA dose recommendation [437]. Nevertheless, although further clinical studies are needed to complete the anticancer activity, ATX supplementation in the human diet has been shown to regulate inflammatory activity [441], enhance the immune response [442], reduce the risk of cardiovascular disease [443], promote eye health, and improve cognitive function [444]. 

A common metabolic alteration in the tumor microenvironment is lipid accumulation, which is associated with immune dysfunction [445]. In this line, the most studied ATX-mediated pathways in humans are the low-density lipoprotein peroxidation and blood lipid profiles, which increase atherosclerosis risk [446]. Moreover, the relation between abnormal lipid metabolism and liver cancer has been demonstrated. In this regard, and in line with animal experimentation, ATX could be a good candidate for hepatocellular carcinoma, although further clinical data are necessary [345].

### 5.5. Fucoxanthin

#### 5.5.1. In Vitro Studies

Previous in vitro studies have reported the anticancer activity of FX in gastrointestinal cancers. In this line, FX has been shown to have growth-inhibitory effects on gastric adenocarcinoma cells by suppression of cyclin B1 and myeloid cell leukemia 1 protein via the JAK/STAT signaling pathway [447,448]. Additionally, the anticancer actions of this carotenoid were associated with autophagy and apoptosis induction through an increase in beclin-1, microtubule-associated protein 1 light chain 3, and cleaved caspase-3, and a reduction in Bcl-2 in gastric cancer cells [449]. Similarly, the cytotoxic activity of FX via up-regulation of autophagy and apoptosis was reported in B666-1 nasopharyngeal cancer cells [450]. Regarding colon cancer, FX exhibited cytotoxic effects in HCT116 and HT29 cells, demonstrating a higher cytotoxicity when the carotenoid was combined with 5-fluorouracil [451]. In addition, FX demonstrated anticancer properties by reducing beta-glucuronidase activity in DLD-1 colorectal cancer cells [452]. Interestingly, fucoxanthinol evidenced a more potent proapoptotic effect than FX in HCT116 cells via suppression of NF-κB activation [453]. Additionally, other studies using FX nanogels or nanoparticles to increase its bioavailability reported that these formulations exhibited a greater pro-oxidative activity than free FX, stimulating ROS-triggered apoptosis in Caco-2 cells [454,455]. In relation to hepatic cancer, FX in combination with cisplatin evidenced a higher antiproliferative activity than treatment with cisplatin alone in human hepatoma HepG2 cells through down-regulation of NF-κB expression as well as an increase in the Bax/Bcl-2 ratio [456]. In the same cell line, an FX-rich fraction from the microalga *Chaetoceros calcitrans* (Bacillariophyta) demonstrated proapoptotic effects via inhibition of antioxidant gene expression and MAPK signaling [457]. 

On the other hand, FX and its metabolite fucoxanthinol inhibited viability in two breast cancer lines, the non-invasive MCF-7 and the invasive MDA-MB-231 cells, by inducing apoptosis. These effects were more prominent with fucoxanthinol and correlated with a suppression of NF-κB pathway activation [458]. Moreover, FX reduced migration and invasion of MDA-MB-231 cells as well as inhibited tumor-induced lymphangiogenesis in human lymphatic endothelial cells [459]. In cervical tumors, FX was reported to have cytotoxic activity in the human cervical cancer cell line HeLa through suppression of the Akt/mechanistic target of rapamycin (mTOR) pathway and the subsequent autophagy induction [460]. Additionally, the mechanisms underlying the proapoptotic effects of FX in HeLa cells were associated with a down-regulation of PI3K/Akt, NF-κB, and the oncogene histone cluster 1 H3 family member [365,461,462]. 

Regarding lung cancer, FX exhibited growth inhibitory effects in several lung carcinoma cell lines by up-regulation of the proapoptotic genes PUMA (p53 up-regulated modulator of apoptosis) and Fas, as well as suppression of Bcl-2 levels [361]. Moreover, this carotenoid induced apoptosis in the human bladder cancer T24 cells via attenuation of mortalin expression, which is considered as an antiapoptotic factor that binds to p53, thus inhibiting its apoptotic activity [463]. Similarly, FX suppressed the mortalin–p53 interaction, leading to p53 nuclear translocation and activation in different cancer cells [464]. This carotenoid and its deacetylated product, fucoxanthinol, also exhibited antiosteosarcoma activity via attenuation of migration and invasion and activation of apoptosis in different osteosarcoma cell lines. The mechanisms underlying these effects may be related to down-regulation of Akt and AP-1 pathways [465]. In relation to skin, the anticancer effects of FX were demonstrated in mouse melanoma B16F10 cells, via cell cycle arrest in the G0/G1 phase and apoptosis induction [366] as well as metastasis inhibition [466]. Furthermore, FX and ATX supressed TPA-induced neoplastic transformation of mouse skin JB6 P+ cells, an in vitro model for tumor promotion, via activation of the Nrf2 pathway [467]. 

As regards central nervous system tumors, FX has been reported to inhibit cell proliferation, invasion, and angiogenesis as well as induced ROS-triggered apoptosis in several glioblastoma cells [468,469]. The molecular antitumorigenic mechanisms of FX involved suppression of PI3K/Akt/mTOR and p38 signaling pathways as well as modulation of the MAPK pathway [364,470]. In relation to B cell malignancies, FX and fucoxanthinol exhibited antiproliferative and proapoptotic effects in Burkitt’s and Hodgkin’s lymphoma cell lines through NF-κB activation with the consequent down-regulation of antiapoptotic proteins (Bcl-2 and X-linked inhibitor of apoptosis protein), and cell cycle regulatory proteins (cyclins D1 and D2) [471]. Similar results were described after treatment of primary effusion lymphoma cells with FX and its metabolite; in addition, their antineoplastic actions were associated with suppression of PI3K/Akt and AP-1 activation [472]. Additionally, the proapoptotic activity of FX was demonstrated in HL-60 leukemia cells due to its pro-oxidative effects and the subsequent down-regulation of the Bcl-xL signalling pathway [473]. Moreover, the antileukemia activity of FX was confirmed in two cancer cell lines representative of advanced stages of chronic myelogenous leukemia [474].

#### 5.5.2. In Vivo Studies

Previous in vivo studies have demonstrated the protective effect of FX in colorectal carcinogenesis. In this respect, FX at the dose of 30 mg/kg for 8 weeks was effective in supressing adenocarcinoma incidence and development of the tumor microenvironment in a model of inflammation-associated colorectal cancer by AOM/DSS [355]. In the same model, these authors demonstrated that FX treatment reduced salivary glycine content over time, suggesting that it may be a good predictor for cancer chemopreventive actions of FX [356]. Additionally, the mechanisms involved in the anti-colon cancer effects of this carotenoid were related to modulation of gut microbiota [357], as well as an induction of anoikis (detachment-induced cell death) though down-regulation of integrin signaling-related proteins [358]. In addition, dietary FX for 5 weeks inhibited colon carcinogenesis in DSS-treated Apc^Min/+^ mice, a model of human familial adenomatous polyposis, by down-regulating cyclin D1 levels [359]. 

Regarding lung cancer, the chemopreventive role of FX was demonstrated in a mouse model of benzo(A)pyrene-induced lung cancer through apoptosis induction by enhanced caspase 9 and 3 levels and reduced expression of Bcl2 protein [360]. Additionally, FX administration at the dose of 50 mg/kg for 5 weeks attenuated A549 tumor xenograft growth in nude mice via apoptosis induction [361]. Furthermore, a recent study demonstrated the antimetastatic activity of FX in a lung metastatic tumor model in A549-bearing mice [362].

On the other hand, FX administered orally for 15 weeks (50 mg/kg) effectively inhibited carcinogenesis in a model of DEN-induced hepatocellular carcinoma in rats via an increase in the endogenous antioxidant defence system [363]. In xenograft models, this carotenoid administered at the dose of 200 mg/kg for 28 days showed antiproliferative and proapoptotic effects as well as reduced invasion and migration in a xenograft of glioblastoma through suppression of PI3K/Akt/mTOR and p38 pathways [364]. In addition, oral administration of FX (10 and 20 mg/kg) for 5 weeks effectively inhibited tumor growth in a cervical cancer xenograft model in nude mice [365]. Similarly, intraperitoneal administration of this carotenoid suppressed melanoma tumor mass in B16F10 cell-injected mice [366]. Additionally, FX exhibited antitumor growth and proapoptotic effects in mice bearing sarcoma 180 xenografts through suppression of STAT3/epidermal growth factor receptor signaling [367].

### 5.6. β-Cryptoxanthin

#### 5.6.1. In Vitro Studies

Several in vitro studies evidenced the antiproliferative, antimigratory, and antiapoptotic effects of BCX in different gastric cancer cells [368,369]. Likewise, this carotenoid inhibited cell viability and induced apoptosis in HCT116 colon cancer cells [475], as well as supressed the migration and invasion of lung cancer cells [372].

#### 5.6.2. In Vivo Studies

Animal studies have demonstrated the chemopreventive effects of BCX in different gastrointestinal cancers. In this regard, oral administration of BCX (5 and 10 mg/kg) for 20 days in a gastric cancer xenograft model in nude mice effectively inhibited tumor growth and angiogenesis and induced apoptosis [369]. Similarly, this carotenoid in combination with the chemotherapeutic drug oxaliplatin exhibited antitumor growth effects on nude mice bearing HCT116 xenografts [475]. Another paper demonstrated that dietary BCX for 24 weeks suppressed the progression of chemically and highly refined carbohydrate diet-induced hepatocellular carcinoma in mice. The mechanisms underlying this effect involved an increase in p53 acetylation, with the subsequent induction of apoptosis and the reduction in HIF-1α and its down-stream targets, MMP-2 and MMP-9 [370]. 

As regards lung cancer, it has been demonstrated that dietary BCX (10 and 20 mg/kg diet) reduced tumor size and multiplicity in a chemically induced lung cancer model via up-regulation of the tumor suppressors SIRT-1, p53, and retinoic acid receptor-β [371]. Later, these authors reported that pre-treatment with BCX supplementation (1 and 10 mg/kg diet) supressed tumor promotion in a model of a nicotine-derived carcinogen-induced lung tumorigenesis through down-regulation of nicotinic acetylcholine receptor α7, highly involved in lung cancer development [372].

#### 5.6.3. Human Studies

Finally, several human studies have described that high serum BCX levels were associated with reduced risk of non-Hodgkin lymphoma [374], colon cancer [375], head and neck cancer [333], breast cancer [376], renal cell carcinoma [377], and lung cancer death in current smokers [373].

## 6. Conclusions

Microalgae have widely drawn scientists’ attention since they are a rich source of bioactive compounds. Their basic and cheap growth requirements make them attractive to be used on a large scale by pharmaceutical, food, and cosmetic industries for health promotion. Carotenoids are one of the most abundant components in microalgae and have been shown to have significant beneficial effects for health. There are two types of carotenoids: carotenes (hydrocabon carotenoids) and xanthophylls (oxygenate derivatives, including ZX, ATX, FX, LUT, α- and BCX, and CX). A multitude of in vitro and in vivo studies and some human studies have evidenced the anti-inflammatory, antioxidant, and antitumor activities of microalgal carotenoids. In this regard, they have been reported to have beneficial effects on many inflammatory diseases, including colitis, non-alcoholic fatty liver, type 2 diabetes mellitus, asthma, arthritis, AMD, AD, and psoriasis, among others. Furthermore, they have been demonstrated to exhibit chemopreventive effects in numerous types of cancer, such as gastric, colon, liver, pancreas, skin, lung, glioblastoma, breast, and prostate. However, further studies, including clinical trials, are required to better evaluate the efficacy and safety of carotenoids and establish recommendations for optimal doses to be used in the prevention and treatment of different inflammatory disorders and cancer.

## Data Availability

Not applicable.

## References

[1] Irigoien X., Hulsman J., Harris R.P. (2004). Global biodiversity patterns of marine phytoplankton and zooplankton. Nature.

[2] Norsker N.H., Barbosa M.J., Vermuë M.H., Wijffels R.H. (2011). Microalgal production—A close look at the economics. Biotechnol. Adv..

[3] Acién F.G., Fernández J.M., Magán J.J., Molina E. (2012). Production cost of a real microalgae production plant and strategies to reduce it. Biotechnol. Adv..

[4] Posten C., Schaub G. (2009). Microalgae and terrestrial biomass as source for fuels—A process view. J. Biotechnol..

[5] Zhu L.D., Li Z.H., Hiltunen E. (2016). Strategies for lipid production improvement in microalgae as a biodiesel feedstock. Biomed Res. Int..

[6] Smith V.H., Sturm B.S.M., deNoyelles F.J., Billings S.A. (2010). The ecology of algal biodiesel production. Trends Ecol. Evol..

[7] Popp J., Harangi-Rákos M., Gabnai Z., Balogh P., Antal G., Bai A. (2016). Biofuels and their co-products as livestock feed: Global economic and environmental implications. Molecules.

[8] Rumin J., Nicolau E., de Oliveira R.G., Fuentes-Grünewald C., Picot L. (2020). Analysis of scientific research driving microalgae market opportunities in Europe. Mar. Drugs.

[9] Raff J.D., Njegic B., Chang W.L., Gordon M.S., Dabdub D., Gerber R.B., Finlayson-Pitts B.J. (2009). Chlorine activation indoors and outdoors via surface-mediated reactions of nitrogen oxides with hydrogen chloride. Proc. Natl. Acad. Sci. USA.

[10] González Y., Torres-Mendoza D., Jones G.E., Fernandez P.L. (2015). Marine diterpenoids as potential anti-inflammatory agents. Mediat. Inflamm..

[11] Eseberri I., Gómez-Zorita S., Trepiana J., González-Arceo M., Aguirre L., Milton-Laskibar I., González M., Fernández-Quintela A., Portillo M.P. (2020). Anti-obesity effects of microalgae. Int. J. Mol. Sci..

[12] Lauritano C., Helland K., Riccio G., Andersen J.H., Ianora A., Hansen E.H. (2020). Lysophosphatidylcholines and chlorophyll-derived molecules from the diatom *Cylindrotheca closterium* with anti-inflammatory activity. Mar. Drugs.

[13] Markou G., Iconomou D., Sotiroudis T., Israilides C., Muylaert K. (2015). Exploration of using stripped ammonia and ash from poultry litter for the cultivation of the cyanobacterium *Arthrospira platensis* and the green microalga *Chlorella vulgaris*. Bioresour. Technol..

[14] Yu X., Chen L., Zhang W. (2015). Chemicals to enhance microalgal growth and accumulation of high-value bioproducts. Front. Microbiol..

[15] Singh N., Roy K., Goyal A., Moholkar V.S. (2019). Investigations in ultrasonic enhancement of β-carotene production by isolated microalgal strain *Tetradesmus obliquus* SGM19. Ultrason. Sonochem..

[16] Ahmad I., Sharma A.K., Daniell H., Kumar S. (2015). Altered lipid composition and enhanced lipid production in green microalga by introduction of brassica diacylglycerol acyltransferase 2. Plant Biotechnol. J..

[17] Venkata Mohan S., Hemalatha M., Chakraborty D., Chatterjee S., Ranadheer P., Kona R. (2020). Algal biorefinery models with self-sustainable closed loop approach: Trends and prospective for blue-bioeconomy. Bioresour. Technol..

[18] PatentScope Database World Intelectual Propiety Organization. https://patentscope.wipo.int/search/es/search.jsf.

[19] Magri M. (2020). Una Nueva Microalga Chlorella Para la Producción de Aceite Vegetal Para Biodiésel y Unidades de Energía de Cogeneración. Spanish Patent.

[20] Fernández Acién G.F., Fernández Sevilla J.M., Molina Grima E., Gómez Serrano C. (2017). Sistema de Eliminación de Metales Pesados en Aguas Mediante Microalgas. Spanish Patent.

[21] Frazao de Andrade A., Figueiredo Porto A.L., De Araujo Viana Marques D., De Lima Filho J.L., Madruga Lima Ribeiro M.H., Nunes Herculano P., Pedrosa Bezerra R., Goncalves De Melo R., Pedrosa Brandão Costa R.M., Da Silva V.A. (2020). Formulação Tópica em Gel Com Atividade Cicatrizante Contendo Extrato de Microalga. British Patent.

[22] Leclere-Bienfait S., Bredif S. (2020). Extract of Chlamydomonas Acidophila, Method for Preparing Same and Cosmetic Compositions and Dermatological Compositions Comprising Same. French Patent.

[23] Herrera Valencia V.A., Peraza Echeverría S., Beltrán Aguilar A.G. (2020). Inducible Crgpdh3 Promoter of Chlamydomonas Reinhardtii and the Ese Thereof for the Expression of Recombinant Proteins. Mexican Patent.

[24] Riquelme Salamanca C.E., Silva Aciares F.R., Gonzalez Cortes L.A., Marticorena de la Rosa P.A. (2019). Método de Cultivo al Exterior u “Outdoor” de la Microalga Muriellopsis sp. para Producir Biomasa Con Alto Contenido en Luteína y Bajo Contenido en Metales Que Tiene Buenas Propiedades Antioxidantes y Util para Preparar Alimento Animal o de Consumo Humano. Chile Patent.

[25] Yueming L., Jianchun X., Lina X., Xiuluan X., Bingzheng X. (2020). Method for Comprehensively Extracting EPA and Fucoxanthin from Phaeodactylum Tricornutum. Chinese Patent.

[26] Nakashima A., Suzuki K., Sugawara T., Manabe Y. (2020). Agent for Suppressing Increment of Blood Glucose Level, Diabetes Preventing Agent, and Food Composition. Japanese Patent.

[27] Napolitano G., Fasciolo G., Salbitani G., Venditti P. (2020). *Chlorella sorokiniana* dietary supplementation increases antioxidant capacities and reduces ros release in mitochondria of hyperthyroid rat liver. Antioxidants.

[28] Talero E., García-Mauriño S., Ávila-Román J., Rodríguez-Luna A., Alcaide A., Motilva V. (2015). Bioactive compounds isolated from microalgae in chronic inflammation and cancer. Mar. Drugs.

[29] Meléndez-Martínez A.J., Stinco C.M., Mapelli-Brahm P. (2019). Skin carotenoids in public health and nutricosmetics: The emerging roles and applications of the UV radiation-absorbing colourless carotenoids phytoene and phytofluene. Nutrients.

[30] Foong L.C., Loh C.W.L., Ng H.S., Lan J.C.W. (2021). Recent development in the production strategies of microbial carotenoids. World J. Microbiol. Biotechnol..

[31] Silva S.C., Ferreira I.C., Dias M., Barreiro M.F. (2020). Microalgae-derived pigments: A 10-year bibliometric review and industry and market trend analysis. Molecules.

[32] Han S.-I., Kim S., Lee C., Choi Y.E. (2019). Blue-red LED wavelength shifting strategy for enhancing beta-carotene production from halotolerant microalga, *Dunaliella salina*. J. Microbiol..

[33] Hassaan M.S., Mohammady E.Y., Soaudy M.R., Sabae S.A., Mahmoud A.M.A., El-Haroun E.R. (2021). Comparative study on the effect of dietary β-carotene and phycocyanin extracted from *Spirulina platensis* on immune-oxidative stress biomarkers, genes expression and intestinal enzymes, serum biochemical in *Nile tilapia*, *Oreochromis niloticus*. Fish Shellfish Immunol..

[34] Rathod J.P., Vira C., Lali A.M., Prakash G. (2020). Metabolic engineering of *Chlamydomonas reinhardtii* for enhanced β-carotene and lutein production. Appl. Biochem. Biotechnol..

[35] Di Lena G., Casini I., Lucarini M., Lombardi-Boccia G. (2019). Carotenoid profiling of five microalgae species from large-scale production. Food Res. Int..

[36] Low K.L., Idris A., Mohd Yusof N. (2020). Novel protocol optimized for microalgae lutein used as food additives. Food Chem..

[37] Jalali Jivan M., Abbasi S. (2019). Nano based lutein extraction from marigold petals: Optimization using different surfactants and co-surfactants. Heliyon.

[38] Wang X., Zhang M.M., Sun Z., Liu S.F., Qin Z.H., Mou J.H., Zhou Z.G., Lin C.S.K. (2020). Sustainable lipid and lutein production from Chlorella mixotrophic fermentation by food waste hydrolysate. J. Hazard. Mater..

[39] Saha S.K., Kazipet N., Murray P. (2018). The carotenogenic Dunaliella salina CCAP 19/20 produces enhanced levels of carotenoid under specific nutrients limitation. Biomed. Res. Int..

[40] Xie Y., Lu K., Zhao X., Ma R., Chen J., Ho S.H. (2019). Manipulating nutritional conditions and salinity-gradient stress for enhanced lutein production in marine microalga *Chlamydomonas* sp.. Biotechnol. J..

[41] Ahmed F., Fanning K., Netzel M., Schenk P.M. (2015). Induced carotenoid accumulation in *Dunaliella salina* and *Tetraselmis suecica* by plant hormones and UV-C radiation. Appl. Microbiol. Biotechnol..

[42] Wojtasiewicz B., Stoń-Egiert J. (2016). Bio-optical characterization of selected cyanobacteria strains present in marine and freshwater ecosystems. J. Appl. Phycol..

[43] Lee M.-Y., Min B.-S., Chang C.-S., Jin E. (2006). Isolation and characterization of a xanthophyll aberrant mutant of the green alga *Nannochloropsis oculata*. Mar. Biotechnol..

[44] Singh D., Puri M., Wilkens S., Mathur A.S., Tuli D.K., Barrow C.J. (2013). Characterization of a new zeaxanthin producing strain of *Chlorella saccharophila* isolated from New Zealand marine waters. Bioresour. Technol..

[45] El-Baz F.K., Hussein R.A., Saleh D.O., Jaleel G.A.R.A. (2019). Zeaxanthin isolated from *Dunaliella salina* microalgae ameliorates age associated cardiac dysfunction in rats through stimulation of retinoid receptors. Mar. Drugs.

[46] Manfellotto F., Stella G.R., Ferrante M.I., Falciatore A., Brunet C. (2020). Engineering the unicellular alga *Phaeodactylum tricornutum* for enhancing carotenoid production. Antioxidants.

[47] Sun K.M., Gao C., Zhang J., Tang X., Wang Z., Zhang X., Li Y. (2020). Rapid formation of antheraxanthin and zeaxanthin in seconds in microalgae and its relation to non-photochemical quenching. Photosynth. Res..

[48] Johnson E.A., An G.H. (1991). Astaxanthin from microbial sources. Crit. Rev. Biotechnol..

[49] Mularczyk M., Michalak I., Marycz K. (2020). Astaxanthin and other nutrients from *Haematococcus pluvialis*—Multifunctional applications. Mar. Drugs.

[50] Mao X., Lao Y., Sun H., Li X., Yu J., Chen F. (2020). Time-resolved transcriptome analysis during transitions of sulfur nutritional status provides insight into triacylglycerol (TAG) and astaxanthin accumulation in the green alga *Chromochloris zofingiensis*. Biotechnol. Biofuels.

[51] Janchot K., Rauytanapanit M., Honda M., Hibino T., Sirisattha S., Praneenararat T., Kageyama H., Waditee-Sirisattha R. (2019). Effects of potassium chloride-induced stress on the carotenoids canthaxanthin, astaxanthin, and lipid accumulations in the green Chlorococcal microalga strain TISTR 9500. J. Eukaryot. Microbiol..

[52] Rajput A., Singh D.P., Khattar J.S., Swatch G.K., Singh Y. (2021). Evaluation of growth and carotenoid production by a green microalga *Scenedesmus quadricauda* PUMCC 4.1.40. under optimized culture conditions. J. Basic Microbiol..

[53] Singh D.P., Khattar J.S., Rajput A., Chaudhary R., Singh R. (2019). High production of carotenoids by the green microalga *Asterarcys quadricellulare* PUMCC 5.1.1 under optimized culture conditions. PLoS ONE.

[54] Méresse S., Fodil M., Fleury F., Chénais B. (2020). Fucoxanthin, a marine-derived carotenoid from brown seaweeds and microalgae: A promising bioactive compound for cancer therapy. Int. J. Mol. Sci..

[55] Bustamam M.S.A., Pantami H.A., Azizan A., Shaari K., Min C.C., Abas F., Nagao N., Maulidiani M., Banerjee S., Sulaiman F. (2021). Complementary analytical platforms of NMR spectroscopy and LCMS analysis in the metabolite profiling *of Isochrysis galbana*. Mar. Drugs.

[56] Lu X., Sun H., Zhao W., Cheng K.W., Chen F., Liu B. (2018). A hetero-photoautotrophic two-stage cultivation process for production of fucoxanthin by the marine diatom *Nitzschia laevis*. Mar. Drugs.

[57] Dogdu Okcu G., Eustance E., Lai Y.J.S., Rittmann B.E. (2021). Evaluation of co-culturing a diatom and a coccolithophore using different silicate concentrations. Sci. Total Environ..

[58] Kanamoto A., Kato Y., Yoshida E., Hasunuma T., Kondo A. (2021). Development of a method for fucoxanthin production using the Haptophyte marine microalga *Pavlova* sp. OPMS 30543. Mar. Biotechnol..

[59] Havaux M., Niyogi K.K. (1999). The violaxanthin cycle protects plants from photooxidative damage by more than one mechanism. Proc. Natl. Acad. Sci. USA.

[60] Park S.B., Yun J.H., Ryu A.J., Yun J., Kim J.W., Lee S., Choi S., Cho D.H., Choi D.Y., Lee Y.J. (2021). Development of a novel *Nannochloropsis* strain with enhanced violaxanthin yield for large-scale production. Microb. Cell Fact..

[61] Ahmad N., Mounsef J.R., Lteif R. (2021). A simple and fast experimental protocol for the extraction of xanthophylls from microalga *Chlorella luteoviridis*. Prep. Biochem. Biotechnol..

[62] Schüler L.M., Bombo G., Duarte P., Santos T.F., Maia I.B., Pinheiro F., Marques J., Jacinto R., Schulze P.S.C., Pereira H. (2021). Carotenoid biosynthetic gene expression, pigment and n-3 fatty acid contents in carotenoid-rich *Tetraselmis striata* CTP4 strains under heat stress combined with high light. Bioresour. Technol..

[63] Martins C.B., Ferreira O., Rosado T., Gallardo E., Silvestre S., Santos L.M.A. (2021). Eustigmatophyte strains with potential interest in cancer prevention and treatment: Partial chemical characterization and evaluation of cytotoxic and antioxidant activity. Biotechnol. Lett..

[64] Lohr M., Wilhelm C. (2001). Xanthophyll synthesis in diatoms: Quantification of putative intermediates and comparison of pigment conversion kinetics with rate constants derived from a model. Planta.

[65] Inbaraj B.S., Chien J.T., Chen B.H. (2006). Improved high performance liquid chromatographic method for determination of carotenoids in the microalga *Chlorella pyrenoidosa*. J. Chromatogr. A.

[66] Markina Z.V., Orlova T.Y., Vasyanovich Y.A., Vardavas A.I., Stivaktakis P.D., Vardavas C.I., Kokkinakis M.N., Rezaee R., Ozcagli E., Golokhvast K.S. (2021). *Porphyridium purpureum* microalga physiological and ultrastructural changes under copper intoxication. Toxicol. Rep..

[67] Juin C., Bonnet A., Nicolau E., Bérard J.B., Devillers R., Thiéry V., Cadoret J.P., Picot L. (2015). UPLC-MSE profiling of phytoplankton metabolites: Application to the identification of pigments and structural analysis of metabolites in *Porphyridium purpureum*. Mar. Drugs.

[68] Rebelo B.A., Farrona S., Ventura M.R., Abranches R. (2020). Canthaxanthin, a red-hot carotenoid: Applications, synthesis, and biosynthetic evolution. Plants.

[69] Lotan T., Hirschberg J. (1995). Cloning and expression in *Escherichia coli* of the gene encoding beta-C-4-oxygenase, that converts beta-carotene to the ketocarotenoid canthaxanthin in *Haematococcus pluvialis*. FEBS Lett..

[70] Hua-Bin L., Fan K.W., Chen F. (2006). Isolation and purification of canthaxanthin from the microalga *Chlorella zofingiensis* by high-speed counter-current chromatography. J. Sep. Sci..

[71] Anila N., Simon D.P., Chandrashekar A., Ravishankar G.A., Sarada R. (2016). Metabolic engineering of *Dunaliella salina* for production of ketocarotenoids. Photosynth. Res..

[72] Kumar T.S., Josephine A., Sreelatha T., Azger Dusthackeer V.N., Mahizhaveni B., Dharani G., Kirubagaran R., Raja Kumar S. (2020). Fatty acids-carotenoid complex: An effective anti-TB agent from the chlorella growth factor-extracted spent biomass of *Chlorella vulgaris*. J. Ethnopharmacol..

[73] Pereira H., Custódio L., Rodrigues M.J., De Sousa C.B., Oliveira M., Barreira L., Neng N.D.R., Nogueira J.M.F., Alrokayan S.A., Mouffouk F. (2015). Biological activities and chemical composition of methanolic extracts of selected autochthonous microalgae strains from the Red Sea. Mar. Drugs.

[74] Grama B.S., Chader S., Khelifi D., Agathos S.N., Jeffryes C. (2014). Induction of canthaxanthin production in a *Dactylococcus* microalga isolated from the Algerian Sahara. Bioresour. Technol..

[75] Germolec D.R., Shipkowski K.A., Frawley R.P., Evans E. (2018). Markers of Inflammation. Methods Mol. Biol..

[76] Panigrahy D., Gilligan M.M., Serhan C.N., Kashfi K. (2021). Resolution of inflammation: An organizing principle in biology and medicine. Pharmacol. Ther..

[77] Chiurchiù V., Leuti A., Maccarrone M. (2018). Bioactive lipids and chronic inflammation: Managing the fire within. Front. Immunol..

[78] Doyle R., Sadlier D.M., Godson C. (2018). Pro-resolving lipid mediators: Agents of anti-ageing?. Semin. Immunol..

[79] Fullerton J.N., Gilroy D.W. (2016). Resolution of inflammation: A new therapeutic frontier. Nat. Rev. Drug Discov..

[80] Sun L., Wang X., Saredy J., Yuan Z., Yang X., Wang H.L. (2020). Innate-adaptive immunity interplay and redox regulation in immune response. Redox Biol..

[81] Mu X., Li Y., Fan G.-C. (2021). Tissue-resident macrophages in the control of infection and resolution of inflammation. Shock.

[82] Mills C.D., Kincaid K., Alt J.M., Heilman M.J., Hill A.M. (2000). M-1/M-2 macrophages and the Th1/Th2 paradigm. J. Immunol..

[83] Sica A., Mantovani A. (2012). Macrophage plasticity and polarization: In vivo veritas. J. Clin. Invest..

[84] Italiani P., Boraschi D. (2014). From monocytes to M1/M2 macrophages: Phenotypical vs. functional differentiation. Front. Immunol..

[85] Adams N.M., Grassmann S., Sun J.C. (2020). Clonal expansion of innate and adaptive lymphocytes. Nat. Rev. Immunol..

[86] Golstein P., Griffiths G.M. (2018). An early history of T cell-mediated cytotoxicity. Nat. Rev. Immunol..

[87] Kourtzelis I., Hajishengallis G., Chavakis T. (2020). Phagocytosis of apoptotic cells in resolution of inflammation. Front. Immunol..

[88] Obata-Ninomiya K., Domeier P.P., Ziegler S.F. (2020). Basophils and eosinophils in nematode infections. Front. Immunol..

[89] Xia M., Wang B., Wang Z., Zhang X., Wang X. (2021). Epigenetic regulation of NK cell-mediated antitumor immunity. Front. Immunol..

[90] Gilroy D.W., Edin M.L., Maeyer R.P.H.D., Bystrom J., Newson J., Lih F.B., Stables M., Zeldin D.C., Bishop-Bailey D. (2016). CYP450-derived oxylipins mediate inflammatory resolution. Proc. Natl. Acad. Sci. USA.

[91] Jaén R.I., Sánchez-García S., Fernández-Velasco M., Boscá L., Prieto P. (2021). Resolution-based therapies: The potential of lipoxins to treat human diseases. Front. Immunol..

[92] Kwon Y. (2020). Immuno-resolving ability of resolvins, protectins, and maresins derived from omega-3 fatty acids in metabolic syndrome. Mol. Nutr. Food Res..

[93] Serhan C.N., Levy B.D. (2018). Resolvins in inflammation: Emergence of the pro-resolving superfamily of mediators. J. Clin. Invest..

[94] Gupta J., Gupta R. (2020). Nutraceutical status and scientific strategies for enhancing production of omega-3 fatty acids from microalgae and their role in healthcare. Curr. Pharm. Biotechnol..

[95] Balkwill F.R., Mantovani A. (2012). Cancer-related inflammation: Common themes and therapeutic opportunities. Semin. Cancer Biol..

[96] Coussens L.M., Werb Z. (2002). Inflammation and cancer. Nature.

[97] Gómez-Valenzuela F., Escobar E., Pérez-Tomás R., Montecinos V.P. (2021). The inflammatory profile of the tumor microenvironment, orchestrated by cyclooxygenase-2, promotes epithelial-mesenchymal transition. Front. Oncol..

[98] Cortese N., Carriero R., Laghi L., Mantovani A., Marchesi F. (2020). Prognostic significance of tumor-associated macrophages: Past, present and future. Semin. Immunol..

[99] Nywening T.M., Wang-Gillam A., Sanford D.E., Belt B.A., Panni R.Z., Cusworth B.M., Toriola A.T., Nieman R.K., Worley L.A., Yano M. (2016). Targeting tumour-associated macrophages with CCR2 inhibition in combination with FOLFIRINOX in patients with borderline resectable and locally advanced pancreatic cancer: A single-centre, open-label, dose-finding, non-randomised, phase 1b trial. Lancet. Oncol..

[100] Cannarile M.A., Weisser M., Jacob W., Jegg A.M., Ries C.H., Rüttinger D. (2017). Colony-stimulating factor 1 receptor (CSF1R) inhibitors in cancer therapy. J. Immunother. Cancer.

[101] Cortese N., Donadon M., Rigamonti A., Marchesi F. (2019). Macrophages at the crossroads of anticancer strategies. Front. Biosci. Landmark.

[102] Vonderheide R.H. (2020). CD40 agonist antibodies in cancer immunotherapy. Annu. Rev. Med..

[103] Advani R., Flinn I., Popplewell L., Forero A., Bartlett N.L., Ghosh N., Kline J., Roschewski M., LaCasce A., Collins G.P. (2018). CD47 blockade by Hu5F9-G4 and rituximab in Non-Hodgkin’s lymphoma. N. Engl. J. Med..

[104] Pan X., Zhang K., Shen C., Wang X., Wang L., Huang Y.Y. (2020). Astaxanthin promotes M2 macrophages and attenuates cardiac remodeling after myocardial infarction by suppression inflammation in rats. Chin. Med. J..

[105] Zbakh H., Zubía E., de Los Reyes C., Calderón-Montaño J.M., Motilva V. (2020). Anticancer activities of meroterpenoids isolated from the brown alga *Cystoseira usneoides* against the human colon cancer cells HT-29. Foods.

[106] Ávila-Román J., Talero E., de los Reyes C., García-Mauriño S., Motilva V. (2018). Microalgae-derived oxylipins decrease inflammatory mediators by regulating the subcellular location of NFκB and PPAR-γ. Pharmacol. Res..

[107] Wang L., Hauenstein A.V. (2020). The NLRP3 inflammasome: Mechanism of action, role in disease and therapies. Mol. Asp. Med..

[108] Ramos-Tovar E., Muriel P. (2020). Molecular mechanisms that link oxidative stress, inflammation, and fibrosis in the liver. Antioxidants.

[109] Holley C.L., Schroder K. (2020). The rOX-stars of inflammation: Links between the inflammasome and mitochondrial meltdown. Clin. Transl. Immunol..

[110] Farkhondeh T., Pourbagher-Shahri A.M., Azimi-Nezhad M., Forouzanfar F., Brockmueller A., Ashrafizadeh M., Talebi M., Shakibaei M., Samarghandian S. (2021). Roles of Nrf2 in gastric cancer: Targeting for therapeutic strategies. Molecules.

[111] Loboda A., Damulewicz M., Pyza E., Jozkowicz A., Dulak J. (2016). Role of Nrf2/HO-1 system in development, oxidative stress response and diseases: An evolutionarily conserved mechanism. Cell. Mol. Life Sci..

[112] Mirzaei S., Mohammadi A.T., Gholami M.H., Hashemi F., Zarrabi A., Zabolian A., Hushmandi K., Makvandi P., Samec M., Liskova A. (2021). Nrf2 signaling pathway in cisplatin chemotherapy: Potential involvement in organ protection and chemoresistance. Pharmacol. Res..

[113] Zimta A.A., Cenariu D., Irimie A., Magdo L., Nabavi S.M., Atanasov A.G., Berindan-Neagoe I. (2019). The role of Nrf2 activity in cancer development and progression. Cancers.

[114] Carambia A., Schuran F.A. (2021). The aryl hydrocarbon receptor in liver inflammation. Semin. Immunopathol..

[115] Bock K.W. (2020). Aryl hydrocarbon receptor (AHR) functions: Balancing opposing processes including inflammatory reactions. Biochem. Pharmacol..

[116] Liu L., Tang Z., Zeng Y., Liu Y., Zhou L., Yang S., Wang D. (2021). Role of necroptosis in infection-related, immune-mediated, and autoimmune skin diseases. J. Dermatol..

[117] Song S., Ding Y., Dai G.L., Zhang Y., Xu M.T., Shen J.R., Chen T.T., Chen Y., Meng G.L. (2020). Sirtuin 3 deficiency exacerbates diabetic cardiomyopathy via necroptosis enhancement and NLRP3 activation. Acta Pharmacol. Sin..

[118] Aggarwal V., Tuli H.S., Varol A., Thakral F., Yerer M.B., Sak K., Varol M., Jain A., Khan M.A., Sethi G. (2019). Role of reactive oxygen species in cancer progression: Molecular mechanisms and recent advancements. Biomolecules.

[119] Lee D.Y., Song M.Y., Kim E.H. (2021). Role of oxidative stress and Nrf2/keap1 signaling in colorectal cancer: Mechanisms and therapeutic perspectives with phytochemicals. Antioxidants.

[120] Tarafdar A., Pula G. (2018). The role of NADPH oxidases and oxidative stress in neurodegenerative disorders. Int. J. Mol. Sci..

[121] Shin J., Song M.H., Oh J.W., Keum Y.S., Saini R.K. (2020). Pro-oxidant actions of carotenoids in triggering apoptosis of cancer cells: A review of emerging evidence. Antioxidants.

[122] Black H.S., Boehm F., Edge R., Truscott T.G. (2020). The benefits and risks of certain dietary carotenoids that exhibit both anti-and pro-oxidative mechanisms—A comprehensive review. Antioxidants.

[123] Ucci M., Di Tomo P., Tritschler F., Cordone V.G.P., Lanuti P., Bologna G., Di Silvestre S., Di Pietro N., Pipino C., Mandatori D. (2019). Anti-inflammatory role of carotenoids in endothelial cells derived from umbilical cord of women affected by gestational diabetes mellitus. Oxid. Med. Cell. Longev..

[124] Di Tomo P., Canali R., Ciavardelli D., Di Silvestre S., De Marco A., Giardinelli A., Pipino C., Di Pietro N., Virgili F., Pandolfi A. (2012). β-Carotene and lycopene affect endothelial response to TNF-α reducing nitro-oxidative stress and interaction with monocytes. Mol. Nutr. Food Res..

[125] Cho S.O., Kim M.-H., Kim H. (2018). β-Carotene inhibits activation of NF-κB, activator protein-1, and STAT3 and regulates abnormal expression of some adipokines in 3T3-L1 adipocytes. J. Cancer Prev..

[126] Lesmana R., Felia Yusuf I., Goenawan H., Achadiyani A., Khairani A.F., Nur Fatimah S., Supratman U. (2020). Low dose of β-carotene regulates inflammation, reduces caspase signaling, and correlates with autophagy activation in cardiomyoblast cell lines. Med. Sci. Monit. Basic Res..

[127] Yang G., Lee H.E., Moon S., Ko K.M., Koh J.H., Seok J.K., Min J., Heo T., Kang H.C., Cho Y. (2020). Direct binding to NLRP3 pyrin domain is a novel strategy to prevent NLRP3-driven inflammation and gouty arthritis. Arthritis Rheumatol..

[128] Li R., Hong P., Zheng X. (2019). β-carotene attenuates lipopolysaccharide-induced inflammation via inhibition of the NF-κB, JAK2/STAT3 and JNK/p38 MAPK signaling pathways in macrophages. Anim. Sci. J..

[129] Lin H.W., Chang T.J., Yang D.J., Chen Y.C., Wang M., Chang Y.Y. (2012). Regulation of virus-induced inflammatory response by β-carotene in RAW264.7 cells. Food Chem..

[130] Trivedi P.P., Jena G.B. (2015). Mechanistic insight into beta-carotene-mediated protection against ulcerative colitis-associated local and systemic damage in mice. Eur. J. Nutr..

[131] Yang Y., Li R., Hui J., Li L., Zheng X. (2021). β-Carotene attenuates LPS-induced rat intestinal inflammation via modulating autophagy and regulating the JAK2/STAT3 and JNK/p38 MAPK signaling pathways. J. Food Biochem..

[132] Li R., Li L., Hong P., Lang W., Hui J., Yang Y., Zheng X. (2021). β-Carotene prevents weaning-induced intestinal inflammation by modulating gut microbiota in piglets. Anim. Biosci..

[133] Latief U., Ahmad R. (2020). β-Carotene inhibits NF-κB and restrains diethylnitrosamine-induced hepatic inflammation in Wistar rats. Int. J. Vitam. Nutr. Res..

[134] El-Din S.H.S., El-Lakkany N.M., El-Naggar A.A., Hammam O.A., El-Latif H.A.A., Ain-Shoka A.A., Ebeid F.A. (2015). Effects of rosuvastatin and/or β-carotene on non-alcoholic fatty liver in rats. Res. Pharm. Sci..

[135] Relevy N.Z., Harats D., Harari A., Ben-Amotz A., Bitzur R., Rühl R., Shaish A. (2015). Vitamin A-deficient diet accelerated atherogenesis in apolipoprotein E(−/−) mice and dietary β -carotene prevents this consequence. Biomed Res. Int..

[136] Kaliappan G., Nagarajan P., Moorthy R., Kalai Gana Selvi S., Avinash Raj T., Mahesh Kumar J. (2013). Ang II induce kidney damage by recruiting inflammatory cells and up regulates PPAR gamma and Renin 1 gene: Effect of β carotene on chronic renal damage. J. Thromb. Thrombolysis.

[137] Takahashi N., Kake T., Hasegawa S., Imai M. (2019). Effects of post-administration of β-carotene on diet-induced atopic dermatitis in hairless mice. J. Oleo Sci..

[138] Kake T., Imai M., Takahashi N. (2019). Effects of β-carotene on oxazolone-induced atopic dermatitis in hairless mice. Exp. Dermatol..

[139] Horváth G., Kemény Á., Barthó L., Molnár P., Deli J., Szente L., Bozó T., Pál S., Sándor K., Szőke É. (2015). Effects of some natural carotenoids on TRPA1- and TRPV1-induced neurogenic inflammatory processes *in vivo* in the mouse skin. J. Mol. Neurosci..

[140] Zhou L., Ouyang L., Lin S., Chen S., Liu Y.J., Zhou W., Wang X. (2018). Protective role of β-carotene against oxidative stress and neuroinflammation in a rat model of spinal cord injury. Int. Immunopharmacol..

[141] Zainal Z., Rahim A.A., Khaza’ai H., Chang S.K. (2019). Effects of palm oil tocotrienol-rich fraction (TRF) and carotenes in ovalbumin (OVA)-challenged asthmatic brown Norway rats. Int. J. Mol. Sci..

[142] Fuke N., Aizawa K., Suganuma H., Takagi T., Naito Y. (2017). Effect of combined consumption of *Lactobacillus brevis* KB290 and β-carotene on minor diarrhoea-predominant irritable bowel syndrome-like symptoms in healthy subjects: A randomised, double-blind, placebo-controlled, parallel-group trial. Int. J. Food Sci. Nutr..

[143] Asemi Z., Alizadeh S.A., Ahmad K., Goli M., Esmaillzadeh A. (2016). Effects of beta-carotene fortified synbiotic food on metabolic control of patients with type 2 diabetes mellitus: A double-blind randomized cross-over controlled clinical trial. Clin. Nutr..

[144] Cho S., Lee D.H., Won C.H., Kim S.M., Lee S., Lee M.J., Chung J.H. (2010). Differential effects of low-dose and high-dose beta-carotene supplementation on the signs of photoaging and type I procollagen gene expression in human skin in vivo. Dermatology.

[145] Ribeiro D., Sousa A., Nicola P., Ferreira de Oliveira J.M.P., Rufino A.T., Silva M., Freitas M., Carvalho F., Fernandes E. (2020). β-Carotene and its physiological metabolites: Effects on oxidative status regulation and genotoxicity in in vitro models. Food Chem. Toxicol..

[146] Wang L., Ding C., Zeng F., Zhu H. (2019). Low levels of serum β-carotene and β-carotene/retinol ratio are associated with histological severity in nonalcoholic fatty liver disease patients. Ann. Nutr. Metab..

[147] Chambaneau A., Filaire M., Jubert L., Bremond M., Filaire E. (2016). Nutritional Intake, Physical Activity and Quality of Life in COPD Patients. Int. J. Sports Med..

[148] Freitas F., Brucker N., Durgante J., Bubols G., Bulcão R., Moro A., Charão M., Baierle M., Nascimento S., Gauer B. (2014). Urinary 1-hydroxypyrene is associated with oxidative stress and inflammatory biomarkers in acute myocardial infarction. Int. J. Environ. Res. Public Health.

[149] Epplein M., Signorello L.B., Zheng W., Cai Q., Hargreaves M.K., Michel A., Pawlita M., Fowke J.H., Correa P., Blot W.J. (2011). *Helicobacter pylori* prevalence and circulating micronutrient levels in a low-income United States population. Cancer Prev. Res..

[150] Muzáková V., Kand’ár R., Meloun M., Skalický J., Královec K., Záková P., Vojtísek P. (2010). Inverse correlation between plasma Beta-carotene and interleukin-6 in patients with advanced coronary artery disease. Int. J. Vitam. Nutr. Res..

[151] Munia I., Gafray L., Bringer M.A., Goldschmidt P., Proukhnitzky L., Jacquemot N., Cercy C., Otman K.R.B., Errera M.H., Ranchon-Cole I. (2020). Cytoprotective effects of natural highly bio-available vegetable derivatives on human-derived retinal cells. Nutrients.

[152] Ge Y., Zhang A., Sun R., Xu J., Yin T., He H., Gou J., Kong J., Zhang Y., Tang X. (2020). Penetratin-modified lutein nanoemulsion *in-situ* gel for the treatment of age-related macular degeneration. Expert Opin. Drug Deliv..

[153] Bian Q., Gao S., Zhou J., Qin J., Taylor A., Johnson E.J., Tang G., Sparrow J.R., Gierhart D., Shang F. (2012). Lutein and zeaxanthin supplementation reduces photooxidative damage and modulates the expression of inflammation-related genes in retinal pigment epithelial cells. Free Radic. Biol. Med..

[154] Li S.Y., Fung F.K.C., Fu Z.J., Wong D., Chan H.H.L., Lo A.C.Y. (2012). Anti-inflammatory effects of lutein in retinal ischemic/hypoxic injury: *In vivo* and *in vitro* studies. Investig. Ophthalmol. Vis. Sci..

[155] Fung F.K.C., Law B.Y.K., Lo A.C.Y. (2016). Lutein attenuates both apoptosis and autophagy upon cobalt (II) chloride-induced hypoxia in rat Muller cells. PLoS ONE.

[156] Chao S.C., Nien C.W., Iacob C., Hu D.N., Huang S.C., Lin H.Y. (2016). Effects of lutein on hyperosmoticity-induced upregulation of IL-6 in cultured corneal epithelial cells and its relevant signal pathways. J. Ophthalmol..

[157] Pongcharoen S., Warnnissorn P., Leŗtkajornsin O., Limpeanchob N., Sutheerawattananonda M. (2013). Protective effect of silk lutein on ultraviolet B-irradiated human keratinocytes. Biol. Res..

[158] Chen C.Y.O., Smith A., Liu Y., Du P., Blumberg J.B., Garlick J. (2017). Photoprotection by pistachio bioactives in a 3-dimensional human skin equivalent tissue model. Int. J. Food Sci. Nutr..

[159] Oh J., Kim J.H., Park J.G., Yi Y., Park K.W., Rho H.S., Lee M., Yoo J.W., Kang S., Hong Y.D. (2013). Radical scavenging activity-based and AP-1-targeted anti-inflammatory effects of lutein in macrophage-like and skin keratinocytic cells. Mediat. Inflamm..

[160] Qiao Y.Q., Jiang P.F., Gao Y.Z. (2018). Lutein prevents osteoarthritis through Nrf2 activation and downregulation of inflammation. Arch. Med. Sci..

[161] Wu W., Li Y., Wu Y., Zhang Y., Wang Z., Liu X. (2015). Lutein suppresses inflammatory responses through Nrf2 activation and NF-κB inactivation in lipopolysaccharide-stimulated BV-2 microglia. Mol. Nutr. Food Res..

[162] Chung R.W.S., Leanderson P., Lundberg A.K., Jonasson L. (2017). Lutein exerts anti-inflammatory effects in patients with coronary artery disease. Atherosclerosis.

[163] Phan M.A.T., Bucknall M., Arcot J. (2018). Effect of different anthocyanidin glucosides on lutein uptake by Caco-2 cells, and their combined activities on anti-oxidation and anti-inflammation *in vitro* and *ex vivo*. Molecules.

[164] Tuzcu M., Orhan C., Muz O.E., Sahin N., Juturu V., Sahin K. (2017). Lutein and zeaxanthin isomers modulates lipid metabolism and the inflammatory state of retina in obesity-induced high-fat diet rodent model. BMC Ophthalmol..

[165] Kamoshita M., Toda E., Osada H., Narimatsu T., Kobayashi S., Tsubota K., Ozawa Y. (2016). Lutein acts via multiple antioxidant pathways in the photo-stressed retina. Sci. Rep..

[166] Wang W., Tam K.C., Ng T.C., Goit R.K., Chan K.L.S., Lo A.C.Y. (2020). Long-term lutein administration attenuates retinal inflammation and functional deficits in early diabetic retinopathy using the Ins2 Akita/+ mice. BMJ Open Diabetes Res. Care.

[167] Sasaki M., Ozawa Y., Kurihara T., Kubota S., Yuki K., Noda K., Kobayashi S., Ishida S., Tsubota K. (2010). Neurodegenerative influence of oxidative stress in the retina of a murine model of diabetes. Diabetologia.

[168] Yeh P.T., Huang H.W., Yang C.M., Yang W.S., Yang C.H. (2016). Astaxanthin inhibits expression of retinal oxidative stress and inflammatory mediators in streptozotocin-induced diabetic rats. PLoS ONE.

[169] Padmanabha S., Vallikannan B. (2018). Fatty acids modulate the efficacy of lutein in cataract prevention: Assessment of oxidative and inflammatory parameters in rats. Biochem. Biophys. Res. Commun..

[170] Padmanabha S., Vallikannan B. (2020). Fatty acids influence the efficacy of lutein in the modulation of α-crystallin chaperone function: Evidence from selenite induced cataract rat model. Biochem. Biophys. Res. Commun..

[171] He R.R., Tsoi B., Lan F., Yao N., Yao X.S., Kurihara H. (2011). Antioxidant properties of lutein contribute to the protection against lipopolysaccharide-induced uveitis in mice. Chin. Med..

[172] Chao S.C., Vagaggini T., Nien C.W., Huang S.C., Lin H.Y. (2015). Effects of lutein and zeaxanthin on LPS-induced secretion of IL-8 by uveal melanocytes and relevant signal pathways. J. Ophthalmol..

[173] Muz O.E., Orhan C., Erten F., Tuzcu M., Ozercan I.H., Singh P., Morde A., Padigaru M., Rai D., Sahin K. (2020). A novel integrated active herbal formulation ameliorates dry eye syndrome by inhibiting inflammation and oxidative stress and enhancing glycosylated phosphoproteins in rats. Pharmaceuticals.

[174] Han H., Cui W., Wang L., Xiong Y., Liu L., Sun X., Hao L. (2015). Lutein prevents high fat diet-induced atherosclerosis in ApoE-deficient mice by inhibiting NADPH oxidase and increasing PPAR expression. Lipids.

[175] Kim J.E., Leite J.O., deOgburn R., Smyth J.A., Clark R.M., Fernandez M.L. (2011). A Lutein-enriched diet prevents cholesterol accumulation and decreases oxidized LDL and inflammatory cytokines in the aorta of guinea pigs. J. Nutr..

[176] Kim J.E., Clark R.M., Park Y., Lee J., Fernandez M.L. (2012). Lutein decreases oxidative stress and inflammation in liver and eyes of guinea pigs fed a hypercholesterolemic diet. Nutr. Res. Pract..

[177] Shimazu Y., Kobayashi A., Endo S., Takemura J., Takeda M. (2019). Effect of lutein on the acute inflammation-induced c-Fos expression of rat trigeminal spinal nucleus caudalis and C1 dorsal horn neurons. Eur. J. Oral Sci..

[178] Syoji Y., Kobayashi R., Miyamura N., Hirohara T., Kubota Y., Uotsu N., Yui K., Shimazu Y., Takeda M. (2018). Suppression of hyperexcitability of trigeminal nociceptive neurons associated with inflammatory hyperalgesia following systemic administration of lutein via inhibition of cyclooxygenase-2 cascade signaling. J. Inflamm..

[179] AbuBakr H.O., Aljuaydi S.H., Abou-Zeid S.M., El-Bahrawy A. (2018). Burn-induced multiple organ injury and protective effect of lutein in rats. Inflammation.

[180] Tan D., Yu X., Chen M., Chen J., Xu J. (2017). Lutein protects against severe traumatic brain injury through anti-inflammation and antioxidative effects via ICAM-1/Nrf-2. Mol. Med. Rep..

[181] Li H., Huang C., Zhu J., Gao K., Fang J., Li H. (2018). Lutein suppresses oxidative stress and inflammation by Nrf2 activation in an osteoporosis rat model. Med. Sci. Monit..

[182] Du S.Y., Zhang Y.L., Bai R.X., Ai Z.L., Xie B.S., Yang H.Y. (2015). Lutein prevents alcohol-induced liver disease in rats by modulating oxidative stress and inflammation. Int. J. Clin. Exp. Med..

[183] Cheng F., Zhang Q., Yan F.F., Wan J.F., Lin C.S. (2015). Lutein protects against ischemia/reperfusion injury in rat skeletal muscle by modulating oxidative stress and inflammation. Immunopharmacol. Immunotoxicol..

[184] Clemons T.E., Sangiovanni J.P., Danis R.P., Frederick L., Iii F., Elman M.J., Antoszyk A., Ruby A., Orth D., Bressler S.B. (2014). Secondary analyses of the effects of lutein/zeaxanthin on age-related macular degeneration progression AREDS2 report No. 3. JAMA Ophthalmol..

[185] Agrón E., Mares J., Clemons T.E., Swaroop A., Chew E.Y., Keenan T.D.L. (2020). Dietary nutrient intake and progression to late age-related macular degeneration in the Age-Related Eye Disease Studies 1 and 2. Ophthalmology.

[186] Korobelnik J.F., Rougier M.B., Delyfer M.N., Bron A., Merle B.M.J., Savel H., Chêne G., Delcourt C., Creuzot-Garcher C. (2017). Effect of dietary supplementation with lutein, zeaxanthin, and ω-3 on macular pigment: A randomized clinical trial. JAMA Ophthalmol..

[187] Huang Y.M., Dou H.L., Huang F.F., Xu X.R., Zou Z.Y., Lin X.M. (2015). Effect of supplemental lutein and zeaxanthin on serum, macular pigmentation, and visual performance in patients with early age-related macular degeneration. Biomed. Res. Int..

[188] Huang Y.M., Dou H.L., Huang F.F., Xu X.R., Zou Z.Y., Lu X.R., Lin X.M. (2015). Changes following supplementation with lutein and zeaxanthin in retinal function in eyes with early age-related macular degeneration: A randomised, double-blind, placebo-controlled trial. Br. J. Ophthalmol..

[189] García-Layana A., Recalde S., Alamán A.S., Robredo P.F. (2013). Effects of lutein and docosahexaenoic acid supplementation on macular pigment optical density in a randomized controlled trial. Nutrients.

[190] Wolf-Schnurrbusch U.E.K., Zinkernagel M.S., Munk M.R., Ebneter A., Wolf S. (2015). Oral lutein supplementation enhances macular pigment density and contrast sensitivity but not in combination with polyunsaturated fatty acids. Investig. Ophthalmol. Vis. Sci..

[191] Grether-Beck S., Marini A., Jaenicke T., Stahl W., Krutmann J. (2017). Molecular evidence that oral supplementation with lycopene or lutein protects human skin against ultraviolet radiation: Results from a double-blinded, placebo-controlled, crossover study. Br. J. Dermatol..

[192] Morse N.L., Reid A.J., St-Onge M. (2018). An open-label clinical trial assessing the efficacy and safety of Bend Skincare Anti-Aging Formula on minimal erythema dose in skin. Photodermatol. Photoimmunol. Photomed..

[193] Granger C., Aladren S., Delgado J., Garre A., Trullas C., Gilaberte Y. (2020). Prospective evaluation of the efficacy of a food supplement in increasing photoprotection and improving selective markers related to skin photo-ageing. Dermatol. Ther..

[194] Liu Y., Xiong Y., Xing F., Gao H., Wang X., He L., Ren C., Liu L., So K.F., Xiao J. (2017). Precise regulation of mir-210 is critical for the cellular homeostasis maintenance and transplantation efficacy enhancement of mesenchymal stem cells in acute liver failure therapy. Cell Transpl..

[195] Zou X., Gao J., Zheng Y., Wang X., Chen C., Cao K., Xu J., Li Y., Lu W., Liu J. (2014). Zeaxanthin induces Nrf2-mediated phase II enzymes in protection of cell death. Cell Death Dis..

[196] Biswal M.R., Justis B.D., Han P., Li H., Gierhart D., Dorey C.K., Lewin A.S. (2018). Daily zeaxanthin supplementation prevents atrophy of the retinal pigment epithelium (RPE) in a mouse model of mitochondrial oxidative stress. PLoS ONE.

[197] Sahin K., Akdemir F., Orhan C., Tuzcu M., Gencoglu H., Sahin N., Ozercan I.H., Ali S., Yilmaz I., Juturu V. (2019). (3R, 3’R)-zeaxanthin protects the retina from photo-oxidative damage via modulating the inflammation and visual health molecular markers. Cutan. Ocul. Toxicol..

[198] Gunal M.Y., Sakul A.A., Caglayan A.B., Erten F., Kursun O.E.D., Kilic E., Sahin K. (2021). Protective effect of lutein/zeaxanthin isomers in traumatic brain injury in mice. Neurotox. Res..

[199] El-Akabawy G., El-Sherif N.M. (2019). Zeaxanthin exerts protective effects on acetic acid-induced colitis in rats via modulation of pro-inflammatory cytokines and oxidative stress. Biomed. Pharmacother..

[200] Firdous A.P., Kuttan G., Kuttan R. (2015). Anti-inflammatory potential of carotenoid meso-zeaxanthin and its mode of action. Pharm. Biol..

[201] Gao H., Lv Y., Liu Y., Li J., Wang X., Zhou Z., Tipoe G.L., Ouyang S., Guo Y., Zhang J. (2019). Wolfberry-derived zeaxanthin dipalmitate attenuates ethanol-induced hepatic damage. Mol. Nutr. Food Res..

[202] Zhou X., Gan T., Fang G., Wang S., Mao Y., Ying C. (2018). Zeaxanthin improved diabetes-induced anxiety and depression through inhibiting inflammation in hippocampus. Metab. Brain Dis..

[203] Majeed M., Majeed S., Nagabhushanam K. (2021). An open-label pilot study on Macumax supplementation for dry-type age-related macular degeneration. J. Med. Food..

[204] Azar G., Quaranta-El Maftouhi M., Masella J.-J., Mauget-Faysse M. (2017). Macular pigment density variation after supplementation of lutein and zeaxanthin using the Visucam^®^ 200 pigment module: Impact of age-related macular degeneration and lens status. J. Fr. Ophtalmol..

[205] Radkar P., Lakshmanan P.S., Mary J.J., Chaudhary S., Durairaj S.K. (2021). A Novel multi-ingredient supplement reduces inflammation of the eye and improves production and quality of tears in humans. Ophthalmol. Ther..

[206] Kishimoto Y., Tani M., Uto-Kondo H., Iizuka M., Saita E., Sone H., Kurata H., Kondo K. (2010). Astaxanthin suppresses scavenger receptor expression and matrix metalloproteinase activity in macrophages. Eur. J. Nutr..

[207] Farruggia C., Kim M., Bae M., Lee Y., Pham T.X., Yang Y., Joo M., Park Y., Lee J. (2018). Astaxanthin exerts anti-inflammatory and antioxidant effects in macrophages in NRF2-dependent and independent manners. J. Nutr. Biochem..

[208] Cai X., Chen Y., Xie X., Yao D., Ding C., Chen M. (2019). Astaxanthin prevents against lipopolysaccharide-induced acute lung injury and sepsis via inhibiting activation of MAPK/NF-κB. Am. J. Transl. Res..

[209] Kang H., Lee Y., Bae M., Park Y.-K., Lee J.-Y. (2020). Astaxanthin inhibits alcohol-induced inflammation and oxidative stress in macrophages in a Sirtuin 1-dependent manner. J. Nutr. Biochem..

[210] Binatti E., Zoccatelli G., Zanoni F., Donà G., Mainente F., Chignola R. (2021). Phagocytosis of astaxanthin-loaded microparticles modulates TGFβ production and intracellular ROS levels in J774A.1 macrophages. Mar. Drugs.

[211] Hyang Y., Koh H., Kim D. (2010). Down-regulation of IL-6 production by astaxanthin via ERK-, MSK-, and NF-κB-mediated signals in activated microglia. Int. Immunopharmacol..

[212] Wen X., Xiao L., Zhong Z., Wang L., Li Z., Pan X. (2017). Astaxanthin acts via LRP-1 to inhibit inflammation and reverse lipopolysaccharide-induced M1/M2 polarization of microglial cells. Oncotarget.

[213] Kim R.E., Shin C.Y., Han S.H., Kwon K.J. (2020). Astaxanthin suppresses PM2.5-induced neuroinflammation by regulating Akt phosphorylation in BV-2 microglial cells. Int. J. Mol. Sci..

[214] Han J.H., Lee Y.S., Im J.H., Ham Y.W., Lee H.P., Han S.B. (2019). Astaxanthin ameliorates lipopolysaccharide-induced neuroinflammation, oxidative stress and memory dysfunction through inactivation of the signal transducer and activator of transcription 3 pathway. Mar. Drugs.

[215] Peng Y.J., Lu J.W., Liu F.C., Lee C.H., Lee H.S., Ho Y.J., Hsieh T.H., Wu C.C., Wang C.C. (2020). Astaxanthin attenuates joint inflammation induced by monosodium urate crystals. FASEB J..

[216] Terazawa S., Nakajima H., Shingo M., Niwano T., Imokawa G. (2012). Astaxanthin attenuates the UVB-induced secretion of prostaglandin E 2 and interleukin-8 in human keratinocytes by interrupting MSK1 phosphorylation in a ROS depletion—Independent manner. Exp. Dermatol..

[217] Li H., Li J., Hou C., Li J., Peng H., Wang Q. (2020). The effect of astaxanthin on inflammation in hyperosmolarity of experimental dry eye model *in vitro* and *in vivo*. Exp. Eye Res..

[218] Wan F.C., Zhang C., Jin Q., Wei C., Zhao H.B., Zhang X.L., You W., Liu X.M., Liu G.F., Liu Y.F. (2020). Protective effects of astaxanthin on lipopolysaccharide-induced inflammation in bovine endometrial epithelial cells. Biol. Reprod..

[219] Kim S.H., Lim J.W., Kim H. (2018). Astaxanthin inhibits mitochondrial dysfunction and and interleukin-8 expression in *Helicobacter pylori*-infected gastric epithelial cells. Nutrients.

[220] Hwang Y., Kim K., Kim S., Mun S., Hong S. (2018). Suppression Effect of Astaxanthin on Osteoclast Formation *In Vitro* and Bone Loss *In Vivo*. Int. J. Mol. Sci..

[221] Bhuvaneswari S., Baskaran Y. (2014). Astaxanthin reduces hepatic endoplasmic reticulum stress and nuclear factor-κB-mediated inflammation in high fructose and high fat diet-fed mice. Cell Stress Chaperones.

[222] Ni Y., Nagashimada M., Zhuge F., Zhan L., Nagata N., Tsutsui A., Nakanuma Y., Kaneko S., Ota T. (2015). Astaxanthin prevents and reverses diet-induced insulin resistance and steatohepatitis in mice: A comparison with vitamin E. Sci. Rep..

[223] Jia Y., Wu C., Kim J., Kim B., Lee S. (2016). Astaxanthin reduces hepatic lipid accumulations in high-fat-fed C57BL/6J mice via activation of peroxisome proliferator-activated receptor (PPAR) alpha and inhibition of PPAR gamma and Akt. J. Nutr. Biochem..

[224] Han J.H., Ju J.H., Lee Y.S., Park J.H., Yeo I.J., Park M.H., Roh Y.S. (2018). Astaxanthin alleviated ethanol-induced liver injury by inhibition of oxidative stress and inflammatory responses via blocking of STAT3 activity. Sci. Rep..

[225] Liu H., Liu M., Fu X., Zhang Z., Zhu L., Zheng X., Liu J. (2018). Astaxanthin prevents alcoholic fatty liver disease by modulating mouse gut microbiota. Nutrients.

[226] Zhang J., Zhang S., Bi J., Gu J., Deng Y., Liu C. (2017). Astaxanthin pretreatment attenuates acetaminophen-induced liver injury in mice. Int. Immunopharmacol..

[227] Shen M., Chen K., Lu J., Cheng P., Xu L., Dai W., Wang F., He L., Zhang Y., Chengfen W. (2014). Protective effect of astaxanthin on liver fibrosis through modulation of TGF- β 1 expression and autophagy. Mediat. Inflamm..

[228] Zhang Z., Guo C., Jiang H., Han B., Wang X., Li S., Lv Y., Lv Z., Zhu Y. (2020). Inflammation response after the cessation of chronic arsenic exposure and post-treatment of natural astaxanthin in liver: Potential role of cytokine-mediated cell-cell interactions. Food Funct..

[229] Iskender H., Yenice G., Terim Kapakin K.A., Dokumacioglu E., Sevim C., Hayirli A., Altun S. (2021). Effects of high fructose diet on lipid metabolism and the hepatic NF-κB/SIRT-1 pathway. Biotech. Histochem..

[230] Chiu C., Chang C., Lin S., Chyau C., Peng R. (2016). improved hepatoprotective effect of liposome-encapsulated astaxanthin in lipopolysaccharide-induced acute hepatotoxicity. Int. J. Mol. Sci..

[231] Guo S., Guo L., Fang Q., Yu M., Zhang L., You C., Wang X., Liu Y., Han C. (2021). Astaxanthin protects against early acute kidney injury in severely burned rats by inactivating the TLR4/MyD88/NF-κB axis and upregulating heme oxygenase-1. Sci. Rep..

[232] Xie W.J., Hou G., Wang L., Wang S.S., Xiong X.X. (2020). Astaxanthin suppresses lipopolysaccharide-induced myocardial injury by regulating MAPK and PI3K/AKT/mTOR/GSK3β signaling. Mol. Med. Rep..

[233] Zhuge F., Ni Y., Wan C., Liu F., Fu Z. (2021). Anti-diabetic effects of astaxanthin on an STZ-induced diabetic model in rats. Endocr. J..

[234] Feng W., Wang Y., Guo N., Huang P., Mi Y. (2020). Effects of astaxanthin on inflammation and insulin resistance in a mouse model of gestational diabetes mellitus. Dose Response.

[235] Liu Y., Yang L., Guo Y., Zhang T., Qiao X., Wang J., Xu J., Xue C. (2020). Hydrophilic astaxanthin: PEGylated astaxanthin fights diabetes by enhancing the solubility and oral absorbability. J. Agric. Food Chem..

[236] Janani R., Anitha R.E., Perumal M.K., Divya P., Baskaran V. (2021). Astaxanthin mediated regulation of VEGF through HIF1α and XBP1 signaling pathway: An insight from ARPE-19 cell and streptozotocin mediated diabetic rat model. Exp. Eye Res..

[237] Xu L., Zhu J., Yin W., Ding X. (2015). Astaxanthin improves cognitive deficits from oxidative stress, nitric oxide synthase and inflammation through upregulation of PI3K/Akt in diabetes rat. Int. J. Clin. Exp. Pathol..

[238] Feng Y., Chu A., Luo Q., Wu M., Shi X., Chen Y. (2018). The protective effect of astaxanthin on cognitive function via inhibition of oxidative stress and inflammation in the brains of chronic T2DM rats. Front. Pharmacol..

[239] Jiang X., Chen L., Shen L., Chen Z., Xu L., Zhang J., Yu X. (2016). Trans-astaxanthin attenuates lipopolysaccharide-induced neuroin fl ammation and depressive-like behavior in mice. Brain Res..

[240] Zhao T., Ma D., Mulati A., Zhao B., Liu F., Liu X. (2021). Development of astaxanthin-loaded layer-by-layer emulsions: Physicochemical properties and improvement of LPS-induced neuroinflammation in mice. Food Funct..

[241] Wang M., Deng X., Xie Y., Chen Y. (2020). Astaxanthin attenuates neuroinflammation in status epilepticus rats by regulating the ATP-P2X7R signal. Drug Des. Devel. Ther..

[242] Zhang X., Zhang X., Zhang Q., Wu Q., Li W., Jiang T., Hang C. (2015). Astaxanthin reduces matrix metalloproteinase-9 expression and activity in the brain after experimental subarachnoid hemorrhage in rats. Brain Res..

[243] Zhang X., Lu Y., Wu Q., Dai H., Li W., Lv S., Zhou X., Zhang X., Hang C., Wang J. (2019). Astaxanthin mitigates subarachnoid hemorrhage injury primarily by increasing sirtuin 1 and inhibiting the Toll-like receptor 4 signaling pathway. FASEB J..

[244] Jiang X., Yan Q., Liu F., Jing C., Ding L., Zhang L. (2018). Chronic trans-astaxanthin treatment exerts antihyperalgesic effect and corrects co-morbid depressive like behaviors in mice with chronic pain. Neurosci. Lett..

[245] Fakhri S., Dargahi L., Abbaszadeh F., Jorjani M. (2018). Astaxanthin attenuates neuroinflammation contributed to the neuropathic pain and motor dysfunction following compression spinal cord injury. Brain Res. Bull..

[246] Fu J., Sun H., Wei H., Dong M., Zhang Y., Xu W., Fang Y., Zhao J. (2020). Astaxanthin alleviates spinal cord ischemia- reperfusion injury via activation of PI3K/Akt/GSK-3β pathway in rats. J. Orthop. Surg. Res..

[247] Chen M.H., Wang T.J., Chen L.J., Jiang M.Y., Wang Y.J., Tseng G.F., Chen J.R. (2021). The effects of astaxanthin treatment on a rat model of Alzheimer’s disease. Brain Res. Bull..

[248] Yang B.-B., Zou M., Zhao L., Zhang Y.-K. (2021). Astaxanthin attenuates acute cerebral infarction via Nrf-2/HO-1 pathway in rats. Curr. Res. Transl. Med..

[249] Sun K., Luo J., Jing X., Guo J., Yao X., Hao X., Ye Y., Lin J., Wang G., Guo F. (2019). Astaxanthin protects against osteoarthritis via Nrf2: A guardian of cartilage homeostasis. Aging.

[250] Kumar A., Dhaliwal N., Dhaliwal J., Dharavath R.N., Chopra K. (2020). Astaxanthin attenuates oxidative stress and inflammatory responses in complete Freund-adjuvant-induced arthritis in rats. Pharmacol. Rep..

[251] Park M.H., Jung J.C., Hill S., Cartwright E., Dohnalek M.H., Yu M., Jun H.J., Han S.B., Hong J.T., Son D.J. (2020). FlexPro MD^®^, a combination of krill oil, astaxanthin and hyaluronic acid, reduces pain behavior and inhibits inflammatory response in monosodium iodoacetate-induced osteoarthritis in rats. Nutrients.

[252] Han H., Lim J.W., Kim H. (2020). Astaxanthin inhibits *Helicobacter pylori*-induced inflammatory and oncogenic responses in gastric mucosal tissues of mice. J. Cancer Prev..

[253] Chen Y., Zhao S., Jiao D., Yao B., Yang S., Li P., Long M. (2021). Astaxanthin alleviates ochratoxin a-induced cecum injury and inflammation in mice by regulating the diversity of cecal microbiota and TLR4/MyD88/NF-κB signaling pathway. Oxid. Med. Cell. Longev..

[254] Yasui Y., Hosokawa M., Mikami N., Miyashita K., Tanaka T. (2011). Dietary astaxanthin inhibits colitis and colitis-associated colon carcinogenesis in mice via modulation of the inflammatory cytokines. Chem. Biol. Interact..

[255] Kochi T., Shimizu M., Sumi T., Kubota M., Shirakami Y., Tanaka T. (2014). Inhibitory effects of astaxanthin on azoxymethane- induced colonic preneoplastic lesions in C57/BL/KsJ- db/db mice. BMC Gastroenterol..

[256] Zhang H., Yang W., Li Y., Hu L., Dai Y., Chen J., Xu S. (2018). Astaxanthin ameliorates cerulein-induced acute pancreatitis in mice. Int. Immunopharmacol..

[257] Hwang Y., Hong S., Mun S., Kim S.-J., Lee S., Kim J., Kang K., Yee S. (2017). The protective effects of astaxanthin on the OVA-induced asthma mice model. Molecules.

[258] Bi J., Cui R., Li Z., Liu C., Zhang J. (2017). Astaxanthin alleviated acute lung injury by inhibiting oxidative/nitrative stress and the inflammatory response in mice. Biomed. Pharmacother..

[259] Xu W., Wang M., Cui G., Li L., Jiao D., Yao B., Xu K., Chen Y., Long M., Yang S. (2019). Astaxanthin protects OTA-induced lung injury in mice through the Nrf2/NF-κB pathway. Toxins.

[260] Park J.H., Yeo I.J., Han J.H., Suh J.W., Lee H.P., Hong J.T. (2018). Anti-inflammatory effect of astaxanthin in phthalic anhydride-induced atopic dermatitis animal model. Exp. Dermatol..

[261] Park J.H., Yeo I.J., Jang J.S., Kim K.C., Park M.H., Lee H.P., Han S., Hong J.T. (2019). Combination effect of titrated extract of *Centella asiatica* and astaxanthin in a mouse model of phthalic anhydride-induced atopic dermatitis. Allergy Asthma Immunol. Res..

[262] Lee Y.S., Jeon S.H., Ham H.J., Lee H.P., Song M.J., Hong J.T. (2020). Improved anti-inflammatory effects of liposomal astaxanthin on a phthalic anhydride-induced atopic dermatitis model. Front. Immunol..

[263] Yoshihisa Y., Andoh T., Matsunaga K., Ur Rehman M., Maoka T., Shimizu T. (2016). Efficacy of astaxanthin for the treatment of atopic dermatitis in a murine model. PLoS ONE.

[264] Harada F., Morikawa T., Lennikov A., Mukwaya A., Schaupper M., Uehara O., Takai R., Yoshida K., Sato J., Horie Y. (2017). Protective effects of oral astaxanthin nanopowder against ultraviolet-induced photokeratitis in mice. Oxid. Med. Cell. Longev..

[265] Fang Q., Guo S., Zhou H., Han R., Wu P., Han C. (2017). Astaxanthin protects against early burn-wound progression in rats by attenuating oxidative stress-induced inflammation and mitochondria-related apoptosis. Sci. Rep..

[266] Tominaga K., Hongo N., Fujishita M., Takahashi Y., Adachi Y. (2017). Protective effects of astaxanthin on skin deterioration. J. Clin. Biochem. Nutr..

[267] Ito N., Seki S., Ueda F. (2018). The protective role of astaxanthin for UV-induced skin deterioration in healthy people—A randomized, double-blind, placebo-controlled trial. Nutrients.

[268] Ito N., Saito H., Seki S., Ueda F., Asada T. (2018). Effects of composite supplement containing astaxanthin and sesamin on cognitive functions in people with mild cognitive impairment: A randomized, double-blind, placebo-controlled trial. J. Alzheimer’s Dis..

[269] Imai A., Oda Y., Ito N., Seki S., Nakagawa K., Miyazawa T., Ueda F. (2018). Effects of dietary supplementation of astaxanthin and sesamin on daily fatigue: A randomized, double-blind, placebo-controlled, two-way crossover study. Nutrients.

[270] Mashhadi N.S., Zakerkish M., Mohammadiasl J., Zarei M., Mohammadshahi M., Haghighizadeh M.H. (2018). Astaxanthin improves glucose metabolism and reduces blood pressure in patients with type 2 diabetes mellitus. Asia Pac. J. Clin. Nutr..

[271] Shokri-mashhadi N., Tahmasebi M., Mohammadiasl J., Zakerkish M., Mohammadshahi M. (2021). The antioxidant and anti-inflammatory effects of astaxanthin supplementation on the expression of miR-146a and miR-126 in patients with type 2 diabetes mellitus: A randomised, double-blind, placebo-controlled clinical trial. Int. J. Clin. Pract..

[272] Heo S., Yoon W., Kim K., Oh C., Choi Y., Yoon K., Kang D., Qian Z., Choi I., Jung W. (2012). Anti-inflammatory effect of fucoxanthin derivatives isolated from *Sargassum siliquastrum* in lipopolysaccharide-stimulated RAW 264.7 macrophage. Food Chem. Toxicol..

[273] Kim K., Heo S., Yoon W., Kang S., Ahn G., Yi T., Jeon Y. (2010). Fucoxanthin inhibits the inflammatory response by suppressing the activation of NF-κB and MAPKs in lipopolysaccharide-induced RAW 264.7 macrophages. Eur. J. Pharmacol..

[274] Li S., Ren X., Wang Y., Hu J., Wu H. (2020). Fucoxanthin alleviates palmitate-induced inflammation in RAW 264.7 cells through improving lipid metabolism and attenuating mitochondrial dysfunction. Food Funct..

[275] Rodríguez-Luna A., Ávila-Román J., Oliveira H., Motilva V., Talero E. (2018). Fucoxanthin-containing cream prevents epidermal hyperplasia and UVB-induced skin erythema in mice. Mar. Drugs.

[276] Pangestuti R., Vo T., Ngo D., Kim S. (2013). Fucoxanthin ameliorates inflammation and oxidative reponses in microglia. J. Agric. Food Chem..

[277] Zhao D., Hwan S., Yoon K., Chun S., Yao M. (2017). Anti-neuroinflammatory effects of fucoxanthin via inhibition of Akt/NF-κB and MAPKs/AP-1 pathways and activation of PKA/CREB pathway in lipopolysaccharide-activated BV-2 microglial cells. Neurochem. Res..

[278] Young S., Sun W., Lee D., Choi G., Yim M., Min J., Jung W., Gwang S., Seo S., Jae S. (2017). Fucoxanthin inhibits pro fibrotic protein expression *in vitro* and attenuates bleomycin-induced lung fibrosis in vivo. Eur. J. Pharmacol..

[279] Rodríguez-Luna A., Ávila-Román J., Oliveira H., Motilva V., Talero E. (2019). Fucoxanthin and rosmarinic acid combination has anti-inflammatory effects through regulation of NLRP3 inflammasome in UVB-exposed HaCaT keratinocytes. Mar. Drugs.

[280] Tavares R.S.N., Kawakami C.M., de Castro Pereira K., do Amaral G.T., Benevenuto C.G., Maria-Engler S.S., Colepicolo P., Debonsi H.M., Gaspar L.R. (2020). Fucoxanthin for topical administration, a phototoxic vs. photoprotective potential in a tiered strategy assessed by in vitro methods. Antioxidants.

[281] Tavares R.S.N., Maria-Engler S.S., Colepicolo P., Debonsi H.M., Schäfer-Korting M., Marx U., Gaspar L.R., Zoschke C. (2020). Skin irritation testing beyond tissue viability: Fucoxanthin effects on inflammation, homeostasis, and metabolism. Pharmaceutics.

[282] Chiang Y.F., Chen H.Y., Chang Y.J., Shih Y.H., Shieh T.M., Wang K.L., Hsia S.M. (2020). Protective effects of fucoxanthin on high glucose and 4-hydroxynonenal (4-HNE)-induced injury in human retinal pigment epithelial cells. Antioxidants.

[283] Hwang P.A., Phan N.N., Lu W.J., Ngoc Hieu B.T., Lin Y.C. (2016). Low-molecular-weight fucoidan and high-stability fucoxanthin from brown seaweed exert prebiotics and anti-inflammatory activities in Caco-2 cells. Food Nutr. Res..

[284] Maeda H., Kanno S., Kodate M., Hosokawa M., Miyashita K. (2015). Fucoxanthinol, metabolite of fucoxanthin, improves obesity-induced inflammation in adipocyte cells. Mar. Drugs.

[285] Seo M., Seo Y., Pan C., Lee O., Kim K., Lee B. (2016). Fucoxanthin suppresses lipid accumulation and ROS production during differentiation in 3T3-L1 adipocytes. Phytother. Res..

[286] Hosokawa M., Miyashita T., Nishikawa S., Emi S., Tsukui T., Beppu F., Okada T., Miyashita K. (2010). Fucoxanthin regulates adipocytokine mRNA expression in white adipose tissue of diabetic/obese KK-Ay mice. Arch. Biochem. Biophys..

[287] Chang Y., Chen Y., Huang W., Liou C. (2018). Fucoxanthin attenuates fatty acid-induced lipid accumulation in FL83B hepatocytes through regulated Sirt1/AMPK signaling pathway. Biochem. Biophys. Res. Commun.

[288] Natsume C., Aoki N., Aoyama T., Senda K., Matsui M., Ikegami A., Tanaka K., Azuma Y.T., Fujita T. (2020). Fucoxanthin ameliorates atopic dermatitis symptoms by regulating keratinocytes and regulatory innate lymphoid cells. Int. J. Mol. Sci..

[289] Choi J., Kim N., Kim S., Lee H., Kim S. (2016). Fucoxanthin inhibits the inflammation response in paw edema model through suppressing MAPKs, Akt, and NF-κB. J. Biochem. Mol. Toxicol..

[290] Yang Y., Tong Q., Zheng S., Zhou M., Zeng Y.-M., Zhou T.-T. (2020). Anti-inflammatory effect of fucoxanthin on dextran sulfate sodium-induced colitis in mice. Nat. Prod. Res..

[291] Gong D., Chu W., Jiang L., Geng C., Li J., Ishikawa N. (2014). Effect of fucoxanthin alone and in combination with d-glucosamine hydrochloride on carrageenan/kaolin-induced experimental arthritis in rats. Phytother. Res..

[292] Jiang X., Wang G., Lin Q., Tang Z., Yan Q., Yu X. (2019). Fucoxanthin prevents lipopolysaccharide-induced depressive-like behavior in mice via AMPK-NF-κB pathway. Metab. Brain Dis..

[293] Li X., Huang R., Liu K., Li M., Luo H., Cui L., Huang L., Luo L. (2020). Fucoxanthin attenuates LPS-induced acute lung injury via inhibition of the TLR4/MyD88 signaling axis. Aging (Albany NY)..

[294] Yang X., Guo G., Dang M., Yan L., Kang X., Jia K., Ren H. (2019). Assessment of the therapeutic effects of fucoxanthin by attenuating inflammation in ovalbumin-induced asthma in an experimental animal model. J. Environ. Pathol. Toxicol. Oncol..

[295] Wu S.-J., Liou C.-J., Chen Y.-L., Cheng S.-C., Huang W.-C. (2021). Fucoxanthin ameliorates oxidative stress and airway inflammation in tracheal epithelial cells and asthmatic mice. Cells.

[296] Tan C., Hou Y. (2014). First evidence for the anti-inflammatory activity of fucoxanthin in high-fat-diet-induced obesity in mice and the antioxidant functions in PC12 cells. Inflammation.

[297] Sun X., Zhao H., Liu Z., Sun X., Zhang D., Wang S., Xu Y., Zhang G., Wang D. (2020). Modulation of gut microbiota by fucoxanthin during alleviation of obesity in high-fat diet-fed mice. J. Agric. Food Chem..

[298] Grasa-López A., Miliar-García Á., Quevedo-Corona L., Paniagua-Castro N., Escalona-Cardoso G., Reyes-Maldonado E. (2016). *Undaria pinnatifida* and fucoxanthin ameliorate lipogenesis and markers of both inflammation and cardiovascular dysfunction in an animal model of diet-induced obesity. Mar. Drugs.

[299] Ha A.W., Na S.J., Kim W.K. (2013). Antioxidant effects of fucoxanthin rich powder in rats fed with high fat diet. Nutr. Res. Pr..

[300] Kong Z., Sudirman S., Hsu Y., Su C., Kuo H. (2019). Fucoxanthin-rich brown algae extract improves male reproductive function on streptozotocin-nicotinamide-induced diabetic rat model. Int. J. Mol. Sci..

[301] Takatani N., Kono Y., Beppu F., Okamatsu-ogura Y. (2020). Fucoxanthin inhibits hepatic oxidative stress, inflammation, and fibrosis in diet-induced nonalcoholic steatohepatitis model mice. Biochem. Biophys. Res. Commun..

[302] Zheng J., Tian X., Zhang W., Zheng P., Huang F. (2019). Protective effects of fucoxanthin against alcoholic liver injury by activation of Nrf2-mediated antioxidant defense and inhibition of TLR4-mediated inflammation. Mar. Drugs.

[303] Shih P.H., Shiue S.J., Chen C.N., Cheng S.W., Lin H.Y., Wu L.W., Wu M.S. (2021). Fucoidan and fucoxanthin attenuate hepatic steatosis and inflammation of NAFLD through modulation of leptin/adiponectin axis. Mar. Drugs.

[304] Orhan C., Tuzcu M., Gencoglu H., Sahin E., Sahin N., Ozercan I.H., Namjoshi T., Srivastava V., Morde A., Rai D. (2021). Different doses of β -cryptoxanthin may secure the retina from photooxidative injury resulted from common LED sources. Oxid. Med. Cell. Longev..

[305] Sahin K., Orhan C., Akdemir F., Tuzcu M., Sahin N., Yılmaz I., Juturu V. (2017). β-Cryptoxanthin ameliorates metabolic risk factors by regulating NF-κB and Nrf2 pathways in insulin resistance induced by high-fat diet in rodents. Food Chem. Toxicol..

[306] Ni Y., Nagashimada M., Zhan L., Nagata N., Kobori M., Sugiura M., Ogawa K., Kaneko S., Ota T. (2015). Prevention and reversal of lipotoxicity-induced hepatic insulin resistance and steatohepatitis in mice by an antioxidant carotenoid, β-cryptoxanthin. Endocrinology.

[307] Zhang F., Shi D., Wang X., Zhang Y., Duan W., Li Y. (2020). β-cryptoxanthin alleviates myocardial ischaemia/reperfusion injury by inhibiting NF-κB-mediated inflammatory signalling in rats. Arch. Physiol. Biochem..

[308] Park G., Horie T., Fukasawa K., Ozaki K., Onishi Y., Kanayama T., Iezaki T., Kaneda K., Sugiura M., Hinoi E. (2017). Amelioration of the development of osteoarthritis by daily intake of β-cryptoxanthin. Biol. Pharm. Bull..

[309] Liu C., Bronson R.T., Russell R.M., Wang X.D. (2011). β-Cryptoxanthin supplementation prevents cigarette smoke-induced lung inflammation, oxidative damage, and squamous metaplasia in ferrets. Cancer Prev. Res..

[310] Haidari F., Hojhabrimanesh A., Helli B., Seyedian S.S., Ahmadi-Angali K. (2020). An energy-restricted high-protein diet supplemented with β-cryptoxanthin alleviated oxidative stress and inflammation in nonalcoholic fatty liver disease: A randomized controlled trial. Nutr. Res..

[311] Lee N.Y., Kim Y., Kim Y.S., Shin J.H., Rubin L.P., Kim Y. (2020). β-Carotene exerts anti-colon cancer effects by regulating M2 macrophages and activated fibroblasts. J. Nutr. Biochem..

[312] Lu M.S., Fang Y.J., Chen Y.M., Luo W.P., Pan Z.Z., Zhong X., Zhang C.X. (2015). Higher intake of carotenoid is associated with a lower risk of colorectal cancer in Chinese adults: A case–control study. Eur. J. Nutr..

[313] Cui B., Liu S., Wang Q., Lin X. (2012). Effect of β-carotene on immunity function and tumour growth in hepatocellular carcinoma rats. Molecules.

[314] Zhang D.M., Luo Y., Yishake D., Liu Z.Y., He T.T., Luo Y., Zhang Y.J., Fang A.P., Zhu H.L. (2020). Prediagnostic dietary intakes of vitamin A and β-carotene are associated with hepatocellular-carcinoma survival. Food Funct..

[315] Lu L., Chen J., Li M., Tang L., Wu R., Jin L., Liang Z. (2018). β-carotene reverses tobacco smoke-induced gastric EMT via Notch pathway *in vivo*. Oncol. Rep..

[316] Kim J.H., Lee J., Choi I.J., Kim Y.-I., Kwon O., Kim H., Kim J. (2018). Dietary carotenoids intake and the risk of gastric cancer: A case—control study in Korea. Nutrients.

[317] Zhang Y., Zhu X., Huang T., Chen L., Liu Y., Li Q., Song J., Ma S., Zhang K., Yang B. (2016). β-Carotene synergistically enhances the anti-tumor effect of 5-fluorouracil on esophageal squamous cell carcinoma *in vivo* and *in vitro*. Toxicol. Lett..

[318] Ge X.X., Xing M.Y., Yu L.F., Shen P. (2013). Carotenoid intake and esophageal cancer risk: A meta-analysis. Asian Pac. J. Cancer Prev..

[319] Yang C.M., Yen Y.T., Huang C.S., Hu M.L. (2011). Growth inhibitory efficacy of lycopene and β-carotene against androgen-independent prostate tumor cells xenografted in nude mice. Mol. Nutr. Food Res..

[320] Lim J.Y., Kim Y.S., Kim K.M., Min S.J., Kim Y. (2014). β-Carotene inhibits neuroblastoma tumorigenesis by regulating cell differentiation and cancer cell stemness. Biochem. Biophys. Res. Commun..

[321] Jain A., Sharma G., Kushwah V., Garg N.K., Kesharwani P., Ghoshal G., Singh B., Shivhare U.S., Jain S., Katare O.P. (2017). Methotrexate and beta-carotene loaded-lipid polymer hybrid nanoparticles: A preclinical study for breast cancer. Nanomedicine.

[322] Jain A., Sharma G., Kushwah V., Ghoshal G., Jain A., Singh B., Shivhare U.S., Jain S., Katare O.P. (2018). Beta carotene-loaded zein nanoparticles to improve the biopharmaceutical attributes and to abolish the toxicity of methotrexate: A preclinical study for breast cancer. Artif. Cells Nanomed. Biotechnol..

[323] He J., Gu Y., Zhang S. (2018). Vitamin A and breast cancer survival: A systematic review and meta-analysis. Clin. Breast Cancer.

[324] Lai G.Y., Weinstein S.J., Albanes D., Taylor P.R., Virtamo J., McGlynn K.A., Freedman N.D. (2014). Association of serum α-tocopherol, β-carotene, and retinol with liver cancer incidence and chronic liver disease mortality. Br. J. Cancer.

[325] Chen F., Hu J., Liu P., Li J., Wei Z., Liu P. (2017). Carotenoid intake and risk of non-Hodgkin lymphoma: A systematic review and dose-response meta-analysis of observational studies. Ann. Hematol..

[326] Reynoso-Camacho R., González-Jasso E., Ferriz-Martínez R., Villalón-Corona B., Loarca-Pina G.F., Salgado L.M., Ramos-Gomez M. (2011). Dietary supplementation of lutein reduces colon carcinogenesis in DMH-treated rats by modulating K-ras, PKB, and β-catenin proteins. Nutr. Cancer.

[327] Kim J., Lee J., Oh J.H., Chang H.J., Sohn D.K., Kwon O., Shin A., Kim J. (2019). Dietary lutein plus zeaxanthin intake and DICER1 rs3742330 A > G polymorphism relative to colorectal cancer risk. Sci. Rep..

[328] Sindhu E.R., Firdous A.P., Ramnath V., Kuttan R. (2013). Effect of carotenoid lutein on N-nitrosodiethylamine-induced hepatocellular carcinoma and its mechanism of action. Eur. J. Cancer Prev..

[329] Baraya Y.S.A., Yankuzo H.M., Wong K.K., Yaacob N.S. (2021). Strobilanthes crispus bioactive subfraction inhibits tumor progression and improves hematological and morphological parameters in mouse mammary carcinoma model. J. Ethnopharmacol..

[330] Yan B., Lu M.S., Wang L., Mo X.F., Luo W.P., Du Y.F., Zhang C.X. (2016). Specific serum carotenoids are inversely associated with breast cancer risk among Chinese women: A case-control study. Br. J. Nutr..

[331] Wu S., Liu Y., Michalek J.E., Mesa R.A., Parma D.L., Rodriguez R., Mansour A.M., Svatek R., Tucker T.C., Ramirez A.G. (2020). Carotenoid intake and circulating carotenoids are inversely associated with the risk of bladder cancer: A dose-response meta-analysis. Adv. Nutr..

[332] Bock C.H., Ruterbusch J.J., Holowatyj A.N., Steck S.E., Van Dyke A.L., Ho W.J., Cote M.L., Hofmann J.N., Davis F., Graubard B.I. (2018). Renal cell carcinoma risk associated with lower intake of micronutrients. Cancer Med..

[333] Leoncini E., Edefonti V., Hashibe M., Parpinel M., Cadoni G., Ferraroni M., Serraino D., Matsuo K., Olshan A.F., Zevallos J.P. (2016). Carotenoid intake and head and neck cancer: A pooled analysis in the International Head and Neck Cancer Epidemiology Consortium. Eur. J. Epidemiol..

[334] Bravi F., Bosetti C., Filomeno M., Levi F., Garavello W., Galimberti S., Negri E., La Vecchia C. (2013). Foods, nutrients and the risk of oral and pharyngeal cancer. Br. J. Cancer.

[335] Jansen R.J., Robinson D.P., Stolzenberg-Solomon R.Z., William R., De Andrade M., Oberg A.L., Rabe K.G., Anderson K.E., Janet E., Sinha R. (2013). Nutrients from fruit and vegetable consumption reduce the risk of pancreatic cancer. J. Gastrointest. Cancer.

[336] Arunkumar R., Gorusupudi A., Li B., Blount J.D., Nwagbo U., Kim H.J., Sparrow J.R., Bernstein P.S. (2021). Lutein and zeaxanthin reduce A2E and iso-A2E levels and improve visual performance in Abca4-/-/Bco2-/- double knockout mice. Exp. Eye Res..

[337] Xu X.L., Hu D.N., Iacob C., Jordan A., Gandhi S., Gierhart D.L., Rosen R. (2015). Effects of zeaxanthin on growth and invasion of human uveal melanoma in nude mouse model. J. Ophthalmol..

[338] Jeurnink S.M., Ros M.M., Leenders M., Van Duijnhoven F.J.B., Siersema P.D., Jansen E.H.J.M., Van Gils C.H., Bakker M.F., Overvad K., Roswall N. (2015). Plasma carotenoids, vitamin C, retinol and tocopherols levels and pancreatic cancer risk within the European Prospective Investigation into Cancer and Nutrition: A nested case-control study: Plasma micronutrients and pancreatic cancer risk. Int. J. Cancer.

[339] Terlikowska K.M., Dobrzycka B., Kinalski M., Terlikowski S.J. (2021). Serum concentrations of carotenoids and fat-soluble vitamins in relation to nutritional status of patients with ovarian cancer. Nutr. Cancer.

[340] Kim D., Kim Y., Kim Y. (2019). Effects of β-carotene on expression of selected MicroRNAs, histone acetylation, and DNA methylation in colon cancer stem cells. J. Cancer Prev..

[341] Ke Y., Bu S., Ma H., Gao L., Cai Y., Zhang Y., Zhou W. (2020). Preventive and therapeutic effects of astaxanthin on depressive-like behaviors in high-fat diet and streptozotocin-treated rats. Front. Pharmacol..

[342] Huang L.J., Chen W.P. (2015). Astaxanthin ameliorates cartilage damage in experimental osteoarthritis. Mod. Rheumatol..

[343] Akduman H., Tayman C., Çakir U., Çakir E., Dilli D., Türkmenoğlu T.T., Gönel A. (2020). Astaxanthin prevents lung injury due to hyperoxia and inflammation. Comb. Chem. High Throughput Screen..

[344] Kim H., Ahn Y.T., Lee G.S., Cho S.I., Kim J.M., Lee C., Lim B.K., Ju S.A., An W.G. (2015). Effects of astaxanthin on dinitrofluorobenzene-induced contact dermatitis in mice. Mol. Med. Rep..

[345] Li J., Guo C., Wu J. (2020). Astaxanthin in liver health and disease: A potential therapeutic agent. Drug Des. Devel. Ther..

[346] Prabhu P.N., Ashokkumar P., Sudhandiran G. (2009). Antioxidative and antiproliferative effects of astaxanthin during the initiation stages of 1,2-dimethyl hydrazine-induced experimental colon carcinogenesis. Fundam. Clin. Pharmacol..

[347] Ohno T., Shimizu M., Shirakami Y., Miyazaki T., Ideta T., Kochi T., Kubota M., Sakai H., Tanaka T., Moriwaki H. (2016). Preventive effects of astaxanthin on diethylnitrosamine-induced liver tumorigenesis in C57/BL/KsJ-db/db obese mice. Hepatol. Res..

[348] Nakao R., Nelson O.L., Park J.S., Mathison B.D., Thompson P.A., Chew B.P. (2010). Effect of dietary astaxanthin at different stages of mammary tumor initiation in BALB/c mice. Anticancer Res..

[349] Nagendraprabhu P., Sudhandiran G. (2011). Astaxanthin inhibits tumor invasion by decreasing extracellular matrix production and induces apoptosis in experimental rat colon carcinogenesis by modulating the expressions of ERK-2, NF-kB and COX-2. Invest. New Drugs.

[350] Cui L., Xu F., Wang M., Li L., Qiao T., Cui H., Li Z., Sun C. (2019). Dietary natural astaxanthin at an early stage inhibits N-nitrosomethylbenzylamine–induced esophageal cancer oxidative stress and inflammation via downregulation of NFκB and COX2 in F344 rats. Onco Targets Ther..

[351] Kavitha K., Thiyagarajan P., Rathna J., Mishra R., Nagini S. (2013). Chemopreventive effects of diverse dietary phytochemicals against DMBA-induced hamster buccal pouch carcinogenesis via the induction of Nrf2-mediated cytoprotective antioxidant, detoxification, and DNA repair enzymes. Biochimie.

[352] Kowshik J., Baba A.B., Giri H., Reddy G.D., Dixit M., Nagini S. (2014). Astaxanthin inhibits JAK/STAT-3 signaling to abrogate cell proliferation, invasion and angiogenesis in a hamster model of oral cancer. PLoS ONE.

[353] Ni X., Yu H., Wang S., Zhang C., Shen S. (2017). Astaxanthin inhibits PC-3 xenograft prostate tumor growth in nude mice. Mar. Drugs.

[354] Haung H.Y., Wang Y.C., Cheng Y.C., Kang W., Hu S.H., Liu D., Xiao C., Wang H.M.D., Ali D. (2020). A novel oral astaxanthin nanoemulsion from *Haematococcus pluvialis* induces apoptosis in lung metastatic melanoma. Oxid. Med. Cell. Longev..

[355] Terasaki M., Masaka S., Fukada C., Houzaki M., Endo T., Tanaka T., Maeda H., Miyashita K., Mutoh M. (2019). Salivary glycine is a significant predictor for the attenuation of polyp and tumor microenvironment formation by fucoxanthin in AOM/DSS mice. In Vivo.

[356] Terasaki M., Kimura R., Kubota A., Kojima H., Tanaka T., Maeda H., Miyashita K., Mutoh M. (2020). Continuity of tumor microenvironmental suppression in AOM/DSS mice by fucoxanthin may be able to track with salivary glycine. In Vivo.

[357] Terasaki M., Uehara O., Ogasa S., Sano T., Kubota A., Kojima H., Tanaka T., Maeda H., Miyashita K., Mutoh M. (2021). Alteration of fecal microbiota by fucoxanthin results in prevention of colorectal cancer in AOM/DSS mice. Carcinogenesis.

[358] Terasaki M., Ikuta M., Kojima H., Tanaka T., Maeda H., Miyashita K., Mutoh M. (2019). Dietary fucoxanthin induces anoikis in colorectal adenocarcinoma by suppressing integrin signaling in a murine colorectal cancer model. J. Clin. Med..

[359] Terasaki M., Hamoya T., Kubota A., Kojima H., Tanaka T., Maeda H., Miyashita K., Mutoh M. (2021). Fucoxanthin prevents colorectal cancer development in dextran sodium sulfate-treated ApcMin/+ mice. Anticancer Res..

[360] Chen W., Zhang H., Liu Y. (2019). Anti-inflammatory and apoptotic signaling effect of fucoxanthin on benzo(A)pyrene-induced lung cancer in mice. J. Environ. Pathol. Toxicol. Oncol..

[361] Mei C.H., Zhou S.C., Zhu L., Ming J.X., Zeng F.D., Xu R. (2017). Antitumor effects of Laminaria extract fucoxanthin on lung cancer. Mar. Drugs.

[362] Ming J.X., Wang Z.C., Huang Y., Ohishi H., Wu R.J., Shao Y., Wang H., Qin M.Y., Wu Z.L., Li Y.Y. (2021). Fucoxanthin extracted from *Laminaria japonica* inhibits metastasis and enhances the sensitivity of lung cancer to gefitinib. J. Ethnopharmacol..

[363] Jin X., Zhao T., Shi D., Ye M.B., Yi Q. (2019). Protective role of fucoxanthin in diethylnitrosamine-induced hepatocarcinogenesis in experimental adult rats. Drug Dev. Res..

[364] Liu Y., Zheng J., Zhang Y., Wang Z., Yang Y., Bai M., Dai Y. (2016). Fucoxanthin activates apoptosis via inhibition of PI3K/Akt/mTOR pathway and suppresses invasion and migration by restriction of p38-MMP-2/9 pathway in human glioblastoma cells. Neurochem. Res..

[365] Ye G., Lu Q., Zhao W., Du D., Jin L., Liu Y. (2014). Fucoxanthin induces apoptosis in human cervical cancer cell line HeLa via PI3K/Akt pathway. Tumor Biol..

[366] Kim K.N., Ahn G., Heo S.J., Kang S.M., Kang M.C., Yang H.M., Kim D., Roh S.W., Kim S.K., Jeon B.T. (2013). Inhibition of tumor growth *in vitro* and *in vivo* by fucoxanthin against melanoma B16F10 cells. Environ. Toxicol. Pharmacol..

[367] Wang J., Chen S., Xu S., Yu X., Ma D., Hu X., Cao X. (2012). *In vivo* induction of apoptosis by fucoxanthin, a marine carotenoid, associated with down-regulating STAT3/EGFR signaling in sarcoma 180 (S180) xenografts-bearing mice. Mar. Drugs.

[368] Wu C., Han L., Riaz H., Wang S., Cai K., Yang L. (2013). The chemopreventive effect of β-cryptoxanthin from mandarin on human stomach cells (BGC-823). Food Chem..

[369] Gao M., Dang F., Deng C. (2019). β-Cryptoxanthin induced anti-proliferation and apoptosis by G0/G1 arrest and AMPK signal inactivation in gastric cancer. Eur. J. Pharmacol..

[370] Lim J.Y., Liu C., Hu K.Q., Smith D.E., Wu D., Lamon-Fava S., Ausman L.M., Wang X.D. (2020). Xanthophyll β-cryptoxanthin inhibits highly refined carbohydrate diet–promoted hepatocellular carcinoma progression in mice. Mol. Nutr. Food Res..

[371] Iskandar A.R., Liu C., Smith D.E., Hu K.Q., Choi S.W., Ausman L.M., Wang X.D. (2013). β-cryptoxanthin restores nicotine-reduced lung SIRT1 to normal levels and inhibits nicotine-promoted lung tumorigenesis and emphysema in A/J mice. Cancer Prev. Res..

[372] Iskandar A.R., Miao B., Li X., Hu K.Q., Liu C., Wang X.D. (2016). β-Cryptoxanthin reduced lung tumor multiplicity and inhibited lung cancer cell motility by downregulating nicotinic acetylcholine receptor A7 signaling. Cancer Prev. Res..

[373] Min K.B., Min J.Y. (2014). Serum carotenoid levels and risk of lung cancer death in US adults. Cancer Sci..

[374] Li S., Zhu X., Zhu L., Hu X., Wen S. (2020). Associations between serum carotenoid levels and the risk of non-Hodgkin lymphoma: A case-control study. Br. J. Nutr..

[375] Huang J., Lu M.S., Fang Y.J., Xu M., Huang W.Q., Pan Z.Z., Chen Y.M., Zhang C.X. (2017). Serum carotenoids and colorectal cancer risk: A case-control study in Guangdong, China. Mol. Nutr. Food Res..

[376] Wang L., Li B., Pan M.X., Mo X.F., Chen Y.M., Zhang C.X. (2014). Specific carotenoid intake is inversely associated with the risk of breast cancer among Chinese women. Br. J. Nutr..

[377] Brock K.E., Ke L., Gridley G., Chiu B.C.H., Ershow A.G., Lynch C.F., Graubard B.I., Cantor K.P. (2012). Fruit, vegetables, fibre and micronutrients and risk of US renal cell carcinoma. Br. J. Nutr..

[378] Jinendiran S., Dahms H.U., Dileep Kumar B.S., Kumar Ponnusamy V., Sivakumar N. (2020). Diapolycopenedioic-acid-diglucosyl ester and keto-myxocoxanthin glucoside ester: Novel carotenoids derived from Exiguobacterium acetylicum S01 and evaluation of their anticancer and anti-inflammatory activities. Bioorg. Chem..

[379] Storniolo C.E., Sacanella I., Lamuela-Raventos R.M., Moreno J.J. (2020). Bioactive compounds of Mediterranean cooked tomato sauce (sofrito) modulate intestinal epithelial cancer cell growth through oxidative stress/arachidonic acid cascade regulation. ACS Omega.

[380] Park Y., Choi J., Lim J.W., Kim H. (2015). β-Carotene-induced apoptosis is mediated with loss of Ku proteins in gastric cancer AGS cells. Genes Nutr..

[381] Park Y., Lee H., Lim J.W., Kim H. (2019). Inhibitory effect of β-carotene on *Helicobacter pylori*-induced TRAF expression and hyper-proliferation in gastric epithelial cells. Antioxidants.

[382] Kim D., Lim J.W., Kim H. (2019). β-carotene Inhibits Expression of c-Myc and Cyclin E in *Helicobacter pylori*-infected Gastric Epithelial Cells. J. Cancer Prev..

[383] Zhu X., Zhang Y., Li Q., Yang L., Zhang N., Ma S., Zhang K., Song J., Guan F. (2016). β-Carotene induces apoptosis in human esophageal squamous cell carcinoma cell lines via the Cav-1/AKT/NF-κB signaling pathway. J. Biochem. Mol. Toxicol..

[384] Ngoc N.B., Lv P., Zhao W.E. (2018). Suppressive effects of lycopene and β-carotene on the viability of the human esophageal squamous carcinoma cell line EC109. Oncol. Lett..

[385] Dutta S., Surapaneni B.K., Bansal A. (2018). Marked inhibition of cellular proliferation in the normal human esophageal epithelial cells and human esophageal squamous cancer cells in culture by carotenoids: Role for prevention and early treatment of esophageal cancer. Asian Pac. J. Cancer Prev..

[386] Huei-Yan C., Chih-Min Y., Jen-Yin C., Te-Cheng Y., Miao-Lin H. (2015). Multicarotenoids at physiological levels inhibit metastasis in human hepatocarcinoma SK-Hep-1 cells. Nutr. Cancer.

[387] Yurtcu E., Iseri O.D., Sahin F.I. (2011). Effects of ascorbic acid and β-carotene on HepG2 human hepatocellular carcinoma cell line. Mol. Biol. Rep..

[388] Kunjiappan S., Panneerselvam T., Somasundaram B., Sankaranarayanan M., Parasuraman P., Joshi S.D., Arunachalam S., Murugan I. (2018). Design graph theoretical analysis and *in silico* modeling of *Dunaliella bardawil* biomass encapsulated N-succinyl chitosan nanoparticles for enhanced anticancer activity. Anticancer. Agents Med. Chem..

[389] Naz H., Khan P., Tarique M., Rahman S., Meena A., Ahamad S., Luqman S., Islam A., Ahmad F., Hassan M.I. (2017). Binding studies and biological evaluation of β-carotene as a potential inhibitor of human calcium/calmodulin-dependent protein kinase IV. Int. J. Biol. Macromol..

[390] Haddad N.F., Teodoro A.J., Leite de Oliveira F., Soares N., de Mattos R.M., Hecht F., Dezonne R.S., Vairo L., dos Santos Goldenberg R.C., Gomes F.C.A. (2013). Lycopene and Beta-carotene induce growth inhibition and proapoptotic effects on ACTH-secreting pituitary adenoma cells. PLoS ONE.

[391] Zhang X., Zhao W.E., Hu L., Zhao L., Huang J. (2011). Carotenoids inhibit proliferation and regulate expression of peroxisome proliferators-activated receptor gamma (PPARγ) in K562 cancer cells. Arch. Biochem. Biophys..

[392] Shiau R.J., Chen K.Y., Der Wen Y., Chuang C.H., Yeh S.L. (2010). Genistein and β-carotene enhance the growth-inhibitory effect of trichostatin A in A549 cells. Eur. J. Nutr..

[393] Sowmya Shree G., Yogendra Prasad K., Arpitha H.S., Deepika U.R., Nawneet Kumar K., Mondal P., Ganesan P. (2017). β-carotene at physiologically attainable concentration induces apoptosis and down-regulates cell survival and antioxidant markers in human breast cancer (MCF-7) cells. Mol. Cell. Biochem..

[394] Gloria N.F., Soares N., Brand C., Oliveira F.L., Borojevic R., Teodoro A.J. (2014). Lycopene and Beta-carotene induce cell-cycle arrest and apoptosis in human breast cancer cell lines. Anticancer Res..

[395] Vijay K., Sowmya P.R.R., Arathi B.P., Shilpa S., Shwetha H.J., Raju M., Baskaran V., Lakshminarayana R. (2018). Low-dose doxorubicin with carotenoids selectively alters redox status and upregulates oxidative stress-mediated apoptosis in breast cancer cells. Food Chem. Toxicol..

[396] Jain A., Sharma G., Thakur K., Raza K., Shivhare U.S., Ghoshal G., Katare O.P. (2019). Beta-carotene-encapsulated solid lipid nanoparticles (BC-SLNs) as promising vehicle for cancer: An investigative assessment. AAPS PharmSciTech..

[397] Lim J.Y., Kim Y.-S., Kim Y. (2013). β-carotene regulates the murine liver microenvironment of a metastatic neuroblastoma. J. Cancer Prev..

[398] Chan M.Y., Lee B.J., Chang P.S., Hsiao H.Y., Hsu L.P., Chang C.H., Lin P.T. (2020). The risks of ubiquinone and β-carotene deficiency and metabolic disorders in patients with oral cancer. BMC Cancer.

[399] Rosa C., Franca C., Vieira S.L., Carvalho A., Penna A., Nogueira C., Lessa S., Ramalho A. (2019). Reduction of serum concentrations and synergy between retinol, β-carotene, and zinc according to cancer staging and different treatment modalities prior to radiation therapy in women with breast cancer. Nutrients.

[400] Nordström T., Van Blarigan E.L., Ngo V., Roy R., Weinberg V., Song X., Simko J., Carroll P.R., Chan J.M., Paris P.L. (2016). Associations between circulating carotenoids, genomic instability and the risk of high-grade prostate cancer. Prostate.

[401] Emri S., Kilickap S., Kadilar C., Halil M.G., Akay H., Besler T. (2012). Serum levels of alpha-tocopherol, vitamin C, beta-carotene, and retinol in malignant pleural mesothelioma. Asian Pac. J. Cancer Prev..

[402] Huang J., Weinstein S.J., Yu K., Männistö S., Albanes D. (2018). Serum Beta-carotene and overall and cause-specific mortality: A prospective cohort study. Circ. Res..

[403] Pantavos A., Ruiter R., Feskens E.F., De Keyser C.E., Hofman A., Stricker B.H., Franco O.H., Kiefte-De Jong J.C. (2015). Total dietary antioxidant capacity, individual antioxidant intake and breast cancer risk: The Rotterdam study. Int. J. Cancer.

[404] Yu N., Su X., Wang Z., Dai B., Kang J. (2015). Association of dietary vitamin A and β-carotene intake with the risk of lung cancer: A meta-analysis of 19 publications. Nutrients.

[405] Tayyem R.F., Mahmoud R.I., Shareef M.H., Marei L.S. (2019). Nutrient intake patterns and breast cancer risk among Jordanian women: A case-control study. Epidemiol. Health.

[406] Middha P., Weinstein S.J., Männistö S., Albanes D., Mondul A.M. (2019). β-Carotene supplementation and lung cancer incidence in the Alpha-tocopherol, Beta-carotene cancer prevention study: The role of tar and nicotine. Nicotine Tob. Res..

[407] Kavalappa Y.P., Gopal S.S., Ponesakki G. (2021). Lutein inhibits breast cancer cell growth by suppressing antioxidant and cell survival signals and induces apoptosis. J. Cell. Physiol..

[408] Muhammad S.N.H., Yaacob N.S., Safuwan N.A.M., Fauzi A.N. (2021). Antiglycolytic activities of *Strobilanthes crispus* active fraction and its bioactive components on triple-negative breast cancer cells *in vitro*. Anticancer Agents Med. Chem..

[409] Gong X., Smith J.R., Swanson H.M., Rubin L.P. (2018). Carotenoid lutein selectively inhibits breast cancer cell growth and potentiates the effect of chemotherapeutic agents through ROS-mediated mechanisms. Molecules.

[410] Li Y., Zhang Y., Liu X., Wang M., Wang P., Yang J., Zhang S. (2018). Lutein inhibits proliferation, invasion and migration of hypoxic breast cancer cells via downregulation of HES1. Int. J. Oncol..

[411] Behbahani M. (2014). Evaluation of *in vitro* anticancer activity of *Ocimum basilicum*, *Alhagi maurorum*, *Calendula officinalis* and their parasite *Cuscuta campestris*. PLoS ONE.

[412] Xu Y., Ma X.Y., Gong W., Li X., Huang H.B., Zhu X.M. (2020). Nanoparticles based on carboxymethylcellulose-modified rice protein for efficient delivery of lutein. Food Funct..

[413] Luan R.L., Wang P.C., Yan M.X., Chen J. (2021). Effect of lutein and doxorubicin combinatorial therapy on S180 cell proliferation and tumor growth. Eur. Rev. Med. Pharmacol. Sci..

[414] Grudzinski W., Piet M., Luchowski R., Reszczynska E., Welc R., Paduch R., Gruszecki W.I. (2018). Different molecular organization of two carotenoids, lutein and zeaxanthin, in human colon epithelial cells and colon adenocarcinoma cells. Spectrochim. Acta Part A Mol. Biomol. Spectrosc..

[415] Rafi M.M., Kanakasabai S., Gokarn S.V., Krueger E.G., Bright J.J. (2015). Dietary lutein modulates growth and survival genes in prostate cancer cells. J. Med. Food.

[416] Zhang W.L., Zhao Y.N., Shi Z.Z., Cong D., Bai Y.S. (2018). Lutein inhibits cell growth and activates apoptosis via the PI3K/AKT/mTOR signaling pathway in A549 human non-small-cell lung cancer cells. J. Environ. Pathol. Toxicol. Oncol..

[417] Zaini R.G., Brandt K., Clench M.R., Le Maitre C.L. (2012). Effects of bioactive compounds from carrots (*Daucus carota* L.), polyacetylenes, Beta-carotene and lutein on human lymphoid leukaemia cells. Anticancer Agents Med. Chem..

[418] Sheng Y.N., Luo Y.H., Liu S.B., Xu W.T., Zhang Y., Zhang T., Xue H., Zuo W.B., Li Y.N., Wang C.Y. (2020). Zeaxanthin induces apoptosis via ROS-regulated MAPK and Akt signaling pathway in human gastric cancer cells. Onco Targets. Ther..

[419] Baudelet P.H., Gagez A.L., Bérard J.B., Juin C., Bridiau N., Kaas R., Thiéry V., Cadoret J.P., Picot L. (2013). Antiproliferative activity of *Cyanophora paradoxa* pigments in melanoma, breast and lung cancer cells. Mar. Drugs.

[420] Bi M.C., Rosen R., Zha R.Y., McCormick S.A., Song E., Hu D.N. (2013). Zeaxanthin induces apoptosis in human uveal melanoma cells through Bcl-2 family proteins and intrinsic apoptosis pathway. Evid. Based Complement. Altern. Med..

[421] Wu N.L., Chiang Y.C., Huang C.C., Fang J.Y., Chen D.F., Hung C.F. (2010). Zeaxanthin inhibits PDGF-BB-induced migration in human dermal fibroblasts. Exp. Dermatol..

[422] Ahn Y.T., Kim M.S., Kim Y.S., An W.G. (2020). Astaxanthin reduces stemness markers in BT20 and T47D breast cancer stem cells by inhibiting expression of pontin and mutant p53. Mar. Drugs.

[423] Badak B., Aykanat N.E.B., Kacar S., Sahinturk V., Arik D., Canaz F. (2021). Effects of astaxanthin on metastasis suppressors in ductal carcinoma. A preliminary study. Ann. Ital. Chir..

[424] Su X.-Z., Chen R., Wang C.-B., Ouyang X.-L., Jiang Y., Zhu M.-Y. (2019). Astaxanthin combine with human serum albumin to abrogate cell proliferation, migration, and drug-resistant in human ovarian carcinoma SKOV3 cells. Anticancer Agents Med. Chem..

[425] Zhao H., Gu H., Zhang H., Li J.H., Zhao W.E. (2014). PPARγ-dependent pathway in the growth-inhibitory effects of K562 cells by carotenoids in combination with rosiglitazone. Biochim. Biophys. Acta Gen. Subj..

[426] Tsuji S., Nakamura S., Maoka T., Yamada T., Imai T., Ohba T., Yako T., Hayashi M., Endo K., Saio M. (2020). Antitumour effects of astaxanthin and adonixanthin on glioblastoma. Mar. Drugs.

[427] Faraone I., Sinisgalli C., Ostuni A., Armentano M.F., Carmosino M., Milella L., Russo D., Labanca F., Khan H. (2020). Astaxanthin anticancer effects are mediated through multiple molecular mechanisms: A systematic review. Pharmacol. Res..

[428] Ferdous U.T., Yusof Z.N.B. (2021). Medicinal prospects of antioxidants from algal sources in cancer therapy. Front. Pharmacol..

[429] Shao Y., Ni Y., Yang J., Lin X., Li J., Zhang L. (2016). Astaxanthin inhibits proliferation and induces apoptosis and cell cycle arrest of mice H22 hepatoma cells. Med. Sci. Monit..

[430] Kim J.H., Park J.J., Lee B.J., Joo M.K., Chun H.J., Lee S.W., Bak Y.T. (2016). Astaxanthin inhibits proliferation of human gastric cancer cell lines by interrupting cell cycle progression. Gut Liver.

[431] Song X., Zhang J., Wang M., Liu W., Gu X., Lv C.-J. (2011). Astaxanthin induces mitochondria-mediated apoptosis in rat hepatocellular carcinoma CBRH-7919 cells. Biol. Pharm. Bull..

[432] Lee H., Lim J.W., Kim H. (2020). Effect of astaxanthin on activation of autophagy and inhibition of apoptosis in *Helicobacter pylori*- infected gastric epithelial cell line AGS. Nutrients.

[433] Hormozi M., Ghoreishi S., Baharvand P. (2019). Astaxanthin induces apoptosis and increases activity of antioxidant enzymes in LS-180 cells. Artif. Cells Nanomed. Biotechnol..

[434] Palozza P., Torelli C., Boninsegna A., Simone R., Catalano A., Mele M.C., Picci N. (2009). Growth-inhibitory effects of the astaxanthin-rich alga *Haematococcus pluvialis* in human colon cancer cells. Cancer Lett..

[435] Sowmya P.R.R., Arathi B.P., Vijay K., Baskaran V., Lakshminarayana R. (2017). Astaxanthin from shrimp efficiently modulates oxidative stress and allied cell death progression in MCF-7 cells treated synergistically with β-carotene and lutein from greens. Food Chem. Toxicol..

[436] Liu X., Song M., Gao Z., Cai X., Dixon W., Chen X., Cao Y., Xiao H. (2016). Stereoisomers of astaxanthin inhibit human colon cancer cell growth by inducing G2/M cell cycle arrest and apoptosis. J. Agric. Food Chem..

[437] Catanzaro E., Bishayee A., Fimognari C. (2020). On a beam of light: Photoprotective activities of the marine carotenoids astaxanthin and fucoxanthin in suppression of inflammation and cancer. Mar. Drugs.

[438] Rao A.R., Sindhuja H.N., Dharmesh S.M., Sankar K.U., Sarada R., Ravishankar G.A. (2013). Effective inhibition of skin cancer, tyrosinase, and antioxidative properties by astaxanthin and astaxanthin esters from the green alga *Haematococcus pluvialis*. J. Agric. Food Chem..

[439] Maoka T., Tokuda H., Suzuki N., Kato H., Etoh H. (2012). Anti-oxidative, anti-tumor-promoting, and anti-carcinogensis activities of nitroastaxanthin and nitrolutein, the reaction products of astaxanthin and lutein with peroxynitrite. Mar. Drugs.

[440] Turck D., Castenmiller J., de Henauw S., Hirsch-Ernst K.I., Kearney J., Maciuk A., Mangelsdorf I., McArdle H.J., Naska A., Pelaez C. (2020). Safety of astaxanthin for its use as a novel food in food supplements. EFSA J..

[441] Desai S.J., Prickril B., Rasooly A. (2018). Mechanisms of phytonutrient modulation of cyclooxygenase-2 (COX-2) and inflammation related to cancer. Nutr. Cancer.

[442] Park J.S., Chyun J.H., Kim Y.K., Line L.L., Chew B.P. (2010). Astaxanthin decreased oxidative stress and inflammation and enhanced immune response in humans. Nutr. Metab..

[443] Pereira C.P.M., Souza A.C.R., Vasconcelos A.R., Prado P.S., Name J.J. (2021). Antioxidant and anti-inflammatory mechanisms of action of astaxanthin in cardiovascular diseases (Review). Int. J. Mol. Med..

[444] Donoso A., González-Durán J., Muñoz A.A., González P.A., Agurto-Muñoz C. (2021). Therapeutic uses of natural astaxanthin: An evidence-based review focused on human clinical trials. Pharmacol. Res..

[445] Xu S., Chaudhary O., Rodríguez-Morales P., Sun X., Chen D., Zappasodi R., Xu Z., Pinto A.F.M., Williams A., Schulze I. (2021). Uptake of oxidized lipids by the scavenger receptor CD36 promotes lipid peroxidation and dysfunction in CD8+ T cells in tumors. Immunity.

[446] Eggersdorfer M., Wyss A. (2018). Carotenoids in human nutrition and health. Arch. Biochem. Biophys..

[447] Yu R.X., Hu X.M., Xu S.Q., Jiang Z.J., Yang W. (2011). Effects of fucoxanthin on proliferation and apoptosis in human gastric adenocarcinoma MGC-803 cells via JAK/STAT signal pathway. Eur. J. Pharmacol..

[448] Yu R.X., Yu R.T., Liu Z. (2018). Inhibition of two gastric cancer cell lines induced by fucoxanthin involves downregulation of Mcl-1 and STAT3. Hum. Cell.

[449] Zhu Y., Cheng J., Min Z., Yin T., Zhang R., Zhang W., Hu L., Cui Z., Gao C., Xu S. (2018). Effects of fucoxanthin on autophagy and apoptosis in SGC-7901cells and the mechanism. J. Cell Biochem..

[450] Long Y., Cao X., Zhao R., Gong S., Jin L., Feng C. (2020). Fucoxanthin treatment inhibits nasopharyngeal carcinoma cell proliferation through induction of autophagy mechanism. Environ. Toxicol..

[451] Lopes-Costa E., Abreu M., Gargiulo D., Rocha E., Ramos A.A. (2017). Anticancer effects of seaweed compounds fucoxanthin and phloroglucinol, alone and in combination with 5-fluorouracil in colon cells. J. Toxicol. Environ. Heal. A.

[452] Kawee-Ai A., Kim S.M. (2014). Application of microalgal fucoxanthin for the reduction of colon cancer risk: Inhibitory activity of fucoxanthin against beta-glucuronidase and DLD-1 cancer cells. Nat. Prod. Commun..

[453] Tamura S., Narita T., Fujii G., Miyamoto S., Hamoya T., Kurokawa Y., Takahashi M., Miki K., Matsuzawa Y., Komiya M. (2019). Inhibition of NF-kappaB transcriptional activity enhances fucoxanthinol-induced apoptosis in colorectal cancer cells. Genes Environ..

[454] Sui Y., Gu Y., Lu Y., Yu C., Zheng J., Qi H. (2021). Fucoxanthin@polyvinylpyrrolidone nanoparticles promoted oxidative stress-induced cell death in Caco-2 human colon cancer cells. Mar. Drugs.

[455] Ravi H., Kurrey N., Manabe Y., Sugawara T., Baskaran V. (2018). Polymeric chitosan-glycolipid nanocarriers for an effective delivery of marine carotenoid fucoxanthin for induction of apoptosis in human colon cancer cells (Caco-2 cells). Mater. Sci. Eng. C.

[456] Liu C.L., Lim Y.P., Hu M.L. (2013). Fucoxanthin enhances cisplatin-induced cytotoxicity via NF-κB-mediated pathway and downregulates DNA repair gene expression in human hepatoma HepG2 cells. Mar. Drugs.

[457] Foo S.C., Yusoff F.M., Imam M.U., Foo J.B., Ismail N., Azmi N.H., Tor Y.S., Khong N.M.H., Ismail M. (2018). Increased fucoxanthin in *Chaetoceros calcitrans* extract exacerbates apoptosis in liver cancer cells via multiple targeted cellular pathways. Biotechnol. Rep..

[458] Rwigemera A., Mamelona J., Martin L.J. (2014). Inhibitory effects of fucoxanthinol on the viability of human breast cancer cell lines MCF-7 and MDA-MB-231 are correlated with modulation of the NF-kappaB pathway. Cell Biol. Toxicol..

[459] Wang J., Ma Y., Yang J., Jin L., Gao Z., Xue L., Hou L., Sui L., Liu J., Zou X. (2019). Fucoxanthin inhibits tumour-related lymphangiogenesis and growth of breast cancer. J. Cell. Mol. Med..

[460] Hou L., Gao C., Chen L., Hu G., Xie S. (2013). Essential role of autophagy in fucoxanthin-induced cytotoxicity to human epithelial cervical cancer HeLa cells. Acta Pharmacol. Sin..

[461] Ye G., Wang L., Yang K., Wang C. (2020). Fucoxanthin may inhibit cervical cancer cell proliferation via downregulation of HIST1H3D. J. Int. Med. Res..

[462] Jin Y., Qiu S., Shao N., Zheng J. (2018). Fucoxanthin and tumor necrosis factor-related apoptosis-inducing ligand (TRAIL) synergistically promotes apoptosis of human cervical cancer cells by targeting PI3K/Akt/NF-κB signaling pathway. Med. Sci. Monit..

[463] Wang L., Zeng Y., Liu Y., Hu X., Li S., Wang Y., Li L., Lei Z., Zhang Z. (2014). Fucoxanthin induces growth arrest and apoptosis in human bladder cancer T24 cells by up-regulation of p21 and down-regulation of mortalin. Acta Biochim. Biophys. Sin..

[464] Garg S., Afzal S., Elwakeel A., Sharma D., Radhakrishnan N., Dhanjal J.K., Sundar D., Kaul S.C., Wadhwa R. (2019). Marine carotenoid fucoxanthin possesses anti-metastasis activity: Molecular evidence. Mar. Drugs.

[465] Rokkaku T., Kimura R., Ishikawa C., Yasumoto T., Senba M., Kanaya F., Mori N. (2013). Anticancer effects of marine carotenoids, fucoxanthin and its deacetylated product, fucoxanthinol, on osteosarcoma. Int. J. Oncol..

[466] Chung T.W., Choi H.J., Lee J.Y., Jeong H.S., Kim C.H., Joo M., Choi J.Y., Han C.W., Kim S.Y., Choi J.S. (2013). Marine algal fucoxanthin inhibits the metastatic potential of cancer cells. Biochem. Biophys. Reses. Commun..

[467] Yang Y., Yang I., Cao M., Su Z., Wu R., Fang M., Kong A., Drugs B., District C.L., City T. (2018). Fucoxanthin elicits epigenetic modifications, Nrf2 activation and blocking transformation in mouse skin JB6 P+ cells. AAPS J..

[468] Lopes F.G., Oliveira K.A., Lopes R.G., Poluceno G.G., Simioni C., Pescador G.D.S., Bauer C.M., Maraschin M., Derner R.B., Garcez R.C. (2020). Anti-cancer effects of fucoxanthin on human glioblastoma cell line. Anticancer Res..

[469] Pruteanu L.L., Kopanitsa L., Módos D., Kletnieks E., Samarova E., Bender A., Gomez L.D., Bailey D.S. (2020). Transcriptomics predicts compound synergy in drug and natural product treated glioblastoma cells. PLoS ONE.

[470] Wu H.L., Fu X.Y., Cao W.Q., Xiang W.Z., Hou Y.J., Ma J.K., Wang Y., Fan C.D. (2019). Induction of apoptosis in human glioma cells by fucoxanthin via triggering of ROS-mediated oxidative damage and regulation of MAPKs and PI3K-AKT pathways. J. Agric. Food Chem..

[471] Tafuku S., Ishikawa C., Yasumoto T., Mori N. (2012). Anti-neoplastic effects of fucoxanthin and its deacetylated product, fucoxanthinol, on Burkitt’s and Hodgkin’s lymphoma cells. Oncol. Rep..

[472] Yamamoto K., Ishikawa C., Katano H., Yasumoto T., Mori N. (2011). Fucoxanthin and its deacetylated product, fucoxanthinol, induce apoptosis of primary effusion lymphomas. Cancer Lett..

[473] Kim K.N., Heo S.J., Kang S.M., Ahn G., Jeon Y.J. (2010). Fucoxanthin induces apoptosis in human leukemia HL-60 cells through a ROS-mediated Bcl-xL pathway. Toxicol. Vitr..

[474] Almeida T.P., Ferreira J., Vettorazzi A., Azqueta A., Rocha E., Ramos A.A. (2018). Cytotoxic activity of fucoxanthin, alone and in combination with the cancer drugs imatinib and doxorubicin, in CML cell lines. Environmen. Toxicol. Pharmacol..

[475] Millán C.S., Soldevilla B., Martín P., Gil-Calderón B., Compte M., Pérez-Sacristán B., Donoso E., Peña C., Romero J., Granado-Lorencio F. (2015). β-Cryptoxanthin synergistically enhances the antitumoral activity of oxaliplatin through ΔNP73 negative regulation in colon cancer. Clin. Cancer Res..

